# Pyridine-3-Carboxamide–1,3,4-Thiadiazole Hybrids as Antimicrobial Agents: Synthesis, Biological Evaluation, In Vivo Toxicity, and Computational Studies

**DOI:** 10.3390/molecules31203700

**Published:** 2026-10-15

**Authors:** Piotr Świątek, Małgorzata Strzelecka, Łukasz Szczukowski, Aneta Jezierska, Jarosław J. Panek, Adam Junka, Aleksandra Woytoń, Roman Lesyk, Teresa Glomb

**Affiliations:** 1 Department of Medicinal Chemistry, Faculty of Pharmacy, Wroclaw Medical University, Borowska 211, 50-556 Wroclaw, Poland; malgorzata.strzelecka@umw.edu.pl (M.S.); lukasz.szczukowski@umw.edu.pl (Ł.S.); teresa.glomb@umw.edu.pl (T.G.); 2 Faculty of Chemistry, University of Wroclaw, F. Joliot-Curie 14, 50-383 Wroclaw, Poland; aneta.jezierska@uwr.edu.pl (A.J.); jaroslaw.panek@uwr.edu.pl (J.J.P.); 3 Platform for Unique Models Application, Department of Translational Technologies, Faculty of Pharmacy, Wroclaw Medical University, Borowska 211, 50-556 Wroclaw, Poland; adam.junka@umw.edu.pl (A.J.); aleksandra.woyton@student.umw.edu.pl (A.W.); 4 College of Life Sciences and Medicine, Zhejiang Sci-Tech University, Hangzhou 310018, China; 5 Department of Pharmaceutical, Organic and Bioorganic Chemistry, Danylo Halytsky Lviv National Medical University, Pekarska 69, 79010 Lviv, Ukraine; dr_r_lesyk@org.lviv.net; 6 Molecular Design Center, Danylo Halytsky Lviv National Medical University, Pekarska 69, 79010 Lviv, Ukraine; 7 Department of Biotechnology and Cell Biology, Medical College, University of Information Technology and Management in Rzeszow, Sucharskiego 2, 35-225 Rzeszow, Poland

**Keywords:** pyridine-3-carboxamide, 1,3,4-thiadiazole, *N*-Mannich base, antimicrobial activity, DFT, QTAIM, molecular docking

## Abstract

The rapid emergence of antimicrobial resistance necessitates the development of novel antimicrobial agents with improved efficacy and favorable safety profiles. In this study, a new series of pyridine-3-carboxamide–1,3,4-thiadiazole hybrids and their corresponding *N*-Mannich bases bearing structurally diverse piperazine substituents were designed, synthesized, and characterized by FT-IR, ^1^H/^13^C NMR, and HR-MS. Their antimicrobial activity was evaluated against reference and clinical bacterial and fungal strains using broth microdilution and agar diffusion assays. The compounds exhibited selective activity against Gram-positive bacteria, with minimum inhibitory concentrations of 0.034–0.069 mg/mL against *Staphylococcus aureus* ATCC 33591 and bactericidal effects reflected by MBC values comparable to MICs. No activity was observed against *Pseudomonas aeruginosa* or *Candida albicans*, and the compounds were ineffective in eradicating preformed staphylococcal biofilms. In vivo toxicity studies using the *Galleria mellonella* model indicated no-to-low apparent acute toxicity under the applied experimental conditions for most derivatives. DFT and QTAIM analyses revealed substituent-dependent electronic and conformational differences, while molecular docking identified DNA gyrase as the most likely molecular target, with compounds **6** and **7** showing the strongest predicted binding. These results identify pyridine-3-carboxamide–1,3,4-thiadiazole Mannich bases as promising leads for developing new antibacterial agents against Gram-positive pathogens.

## 1. Introduction

Antimicrobial resistance (AMR) is a major global public health threat and an increasing challenge for modern medicine. The inappropriate use of antimicrobial agents, including unnecessary prescribing, incorrect dosing, and poor patient adherence, has contributed to the emergence and spread of drug-resistant microorganisms. AMR occurs when microorganisms develop or acquire mechanisms that enable them to survive exposure to antimicrobial agents that would otherwise inhibit their growth or eliminate them. These mechanisms may include enzymatic drug inactivation, modification of antimicrobial targets, reduced membrane permeability, active efflux, and biofilm formation. Infections caused by resistant microorganisms are associated with limited therapeutic options, prolonged hospitalization, increased healthcare costs, and higher morbidity and mortality. They also compromise the safety of surgical procedures, organ transplantation, cancer chemotherapy, and other medical interventions that depend on effective antimicrobial prophylaxis and treatment [1,2,3,4,5,6,7,8,9].

One strategy for addressing antimicrobial resistance is the development of new antimicrobial agents with activity against drug-resistant microorganisms. Researchers worldwide are therefore investigating novel compounds, particularly those with new molecular targets or mechanisms of action that may reduce cross-resistance with currently used drugs.

**Figure 1:**
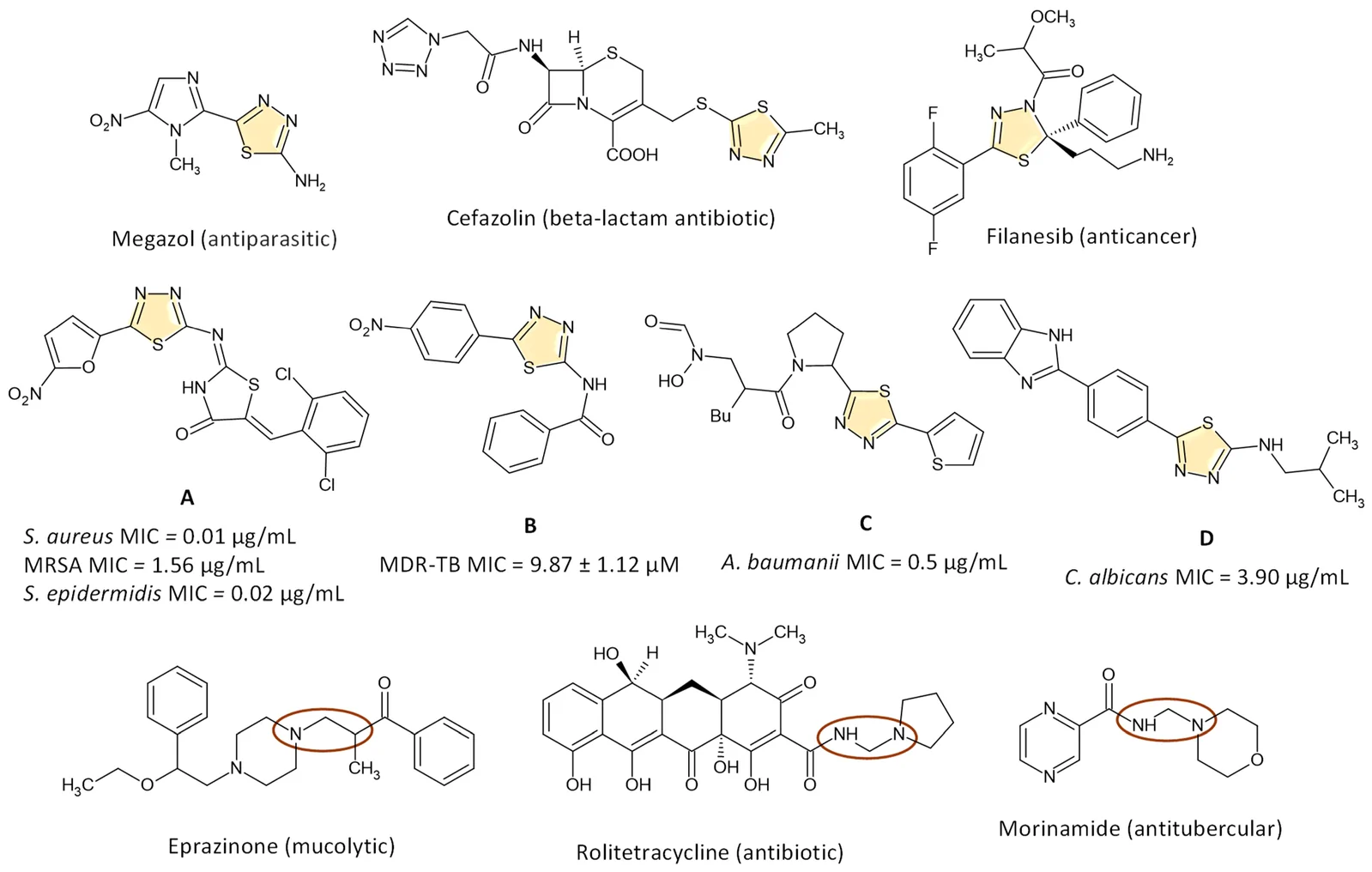
General chemical structures of representative drugs and antimicrobial compounds characterized by the presence of the 1,3,4-thiadiazole ring and structures of representative Mannich bases used as pharmaceuticals.

Heterocyclic compounds constitute important classes of organic molecules, characterized by cyclic structures containing one or more heteroatoms. Among them, sulfur-containing heterocycles have attracted considerable attention owing to their broad pharmacological potential, particularly their antimicrobial and anticancer activities [10,11,12]. One of the most extensively investigated scaffolds is the thiadiazole ring. The presence of nitrogen and sulfur heteroatoms provides favorable electronic properties that enable interactions with a wide range of biological targets [13,14]. Among the four possible thiadiazole isomers, the 1,3,4-thiadiazole ring is the most thermodynamically stable and has therefore emerged as a privileged scaffold in medicinal chemistry [14]. Numerous studies have demonstrated that 1,3,4-thiadiazole derivatives exhibit a broad spectrum of biological activities, including antibacterial [15,16,17], antifungal [18,19], antituberculosis [20,21], anticancer [22], antioxidant [23], analgesic and anti-inflammatory [24,25]. The pharmaceutical relevance of the 1,3,4-thiadiazole scaffold is reflected by its presence in several clinically used drugs, including megazole, cefazolin, and filanesib (Figure 1). In addition to medicinal applications, 1,3,4-thiadiazole derivatives have also found widespread use in agriculture as fungicides, herbicides, insecticides, bactericides, and pesticides, highlighting the versatility of this heterocyclic scaffold [26].

Among the various biological activities reported for 1,3,4-thiadiazole derivatives, their antimicrobial properties have attracted considerable interest in the search for compounds that are active against drug-resistant microorganisms. The antimicrobial activity of these derivatives varies depending on their substitution pattern, physicochemical properties, and the susceptibility of the tested microorganism. Several mechanisms have been proposed for individual thiadiazole-containing compounds; however, these mechanisms cannot necessarily be generalized to the entire chemical class. For example, compound **A** showed promising activity against the tested Gram-positive bacteria, including methicillin-resistant *Staphylococcus aureus* (MRSA), with an MIC value of 1.56 µg/mL [16]. Another thiadiazole derivative, **B**, showed significant anti-mycobacterial activity against *M. tuberculosis* H37Rv and the resistant phenotypic MDR-TB strains [20]. Notably, thiadiazole-based InhA inhibitors exhibited nanomolar inhibitory activity against the enoyl-acyl carrier protein reductase (InhA), which translated into excellent antimycobacterial potency [21]. Recently, incorporation of the 1,3,4-thiadiazole ring into peptide deformylase inhibitors afforded broad-spectrum antibacterial agents, with compound **C** showing remarkable protective efficacy against drug-resistant *A.baumannii* in a mouse infection model [17]. Furthermore, benzimidazole–thiadiazole hybrid **D** has been reported to exhibit potent antifungal activity against *C. albicans* (MIC = 3.90 μg/mL), which has been attributed to its inhibition of sterol 14α-demethylase [18]. The structures of selected biologically active 1,3,4-thiadiazole derivatives are shown in Figure 1.

On the other hand, an attractive strategy for enhancing the biological activity of heterocyclic compounds involves the introduction of aminomethyl substituents via the Mannich reaction. *N*-Mannich bases derived from diverse heterocyclic systems have been extensively investigated because of their broad spectrum of biological activities, particularly their antimicrobial potential [27,28]. Incorporation of a Mannich base often improves physicochemical properties, modulates interactions with multiple biological targets, and enhances pharmacological activity, making these compounds valuable candidates for drug discovery [29]. The therapeutic importance of this approach is underscored by several FDA-approved heterocyclic drugs containing Mannich base motifs, representative examples of which are presented in Figure 1.

**Figure 2:**
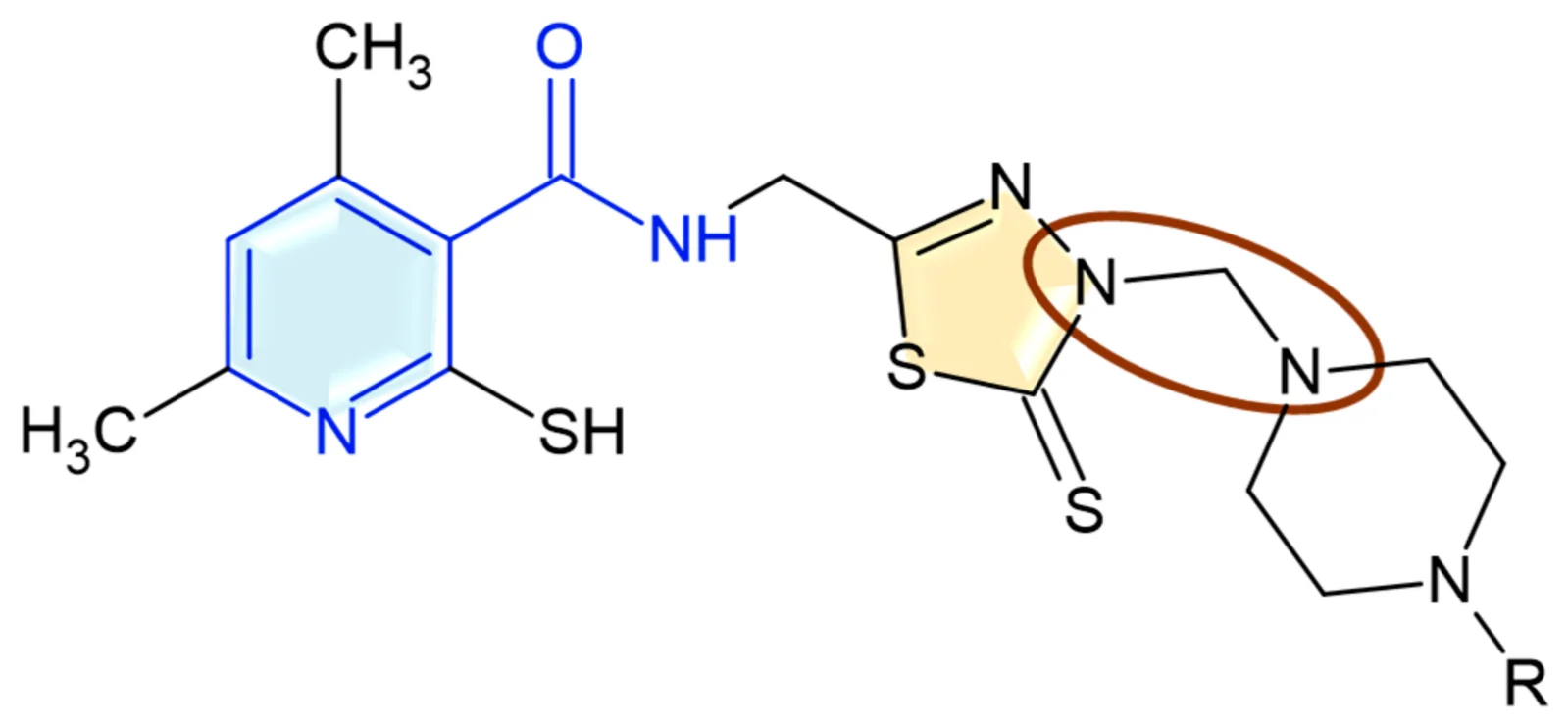
General structure of the target compounds.

Based on our previous studies demonstrating the promising biological potential of pyridine-3-carboxamide derivatives [30,31,32] and our continued interest in the synthesis and biological evaluation of novel heterocyclic molecules, we designed a new series of hybrid compounds combining a pyridine-3-carboxamide scaffold with a 1,3,4-thiadiazole ring. The design of these compounds was also inspired by our earlier studies on isothiazolopyridine derivatives, which were investigated for their antibacterial activity [33,34]. The newly synthesized pyridine-3-carboxamide derivatives may therefore be regarded as ring-opened analogs of these isothiazolopyridine compounds, enabling assessment of how this structural modification influences antibacterial activity. To further expand the structural diversity of this series, *N*-Mannich bases bearing various substituted piperazine moieties were synthesized via a three-component Mannich reaction. The general structure of the target compounds is presented in Figure 2.

The synthesized derivatives were subsequently evaluated for their antimicrobial activity. Their in vivo toxicity was assessed using the *Galleria mellonella* larval model. The third part of the study consists of in silico experiments, which could be divided into four parts: (a) Density Functional Theory (DFT)-based structure determination; (b) electron density analysis using Quantum Theory of Atoms in Molecules (QTAIM); (c) computation of selected physicochemical descriptors, Absorption, Distribution, Metabolism, Excretion (ADME) parameters prediction, pharmacokinetic properties, druglikeness and medicinal chemistry friendliness; and (d) molecular docking. The combination of various experimental and theoretical approaches provides a more comprehensive explanation of molecular features and possible mechanisms that are responsible for biological activity in the set of the investigated molecules.

## 2. Results and Discussion

### 2.1. Chemistry

**Scheme 1:**
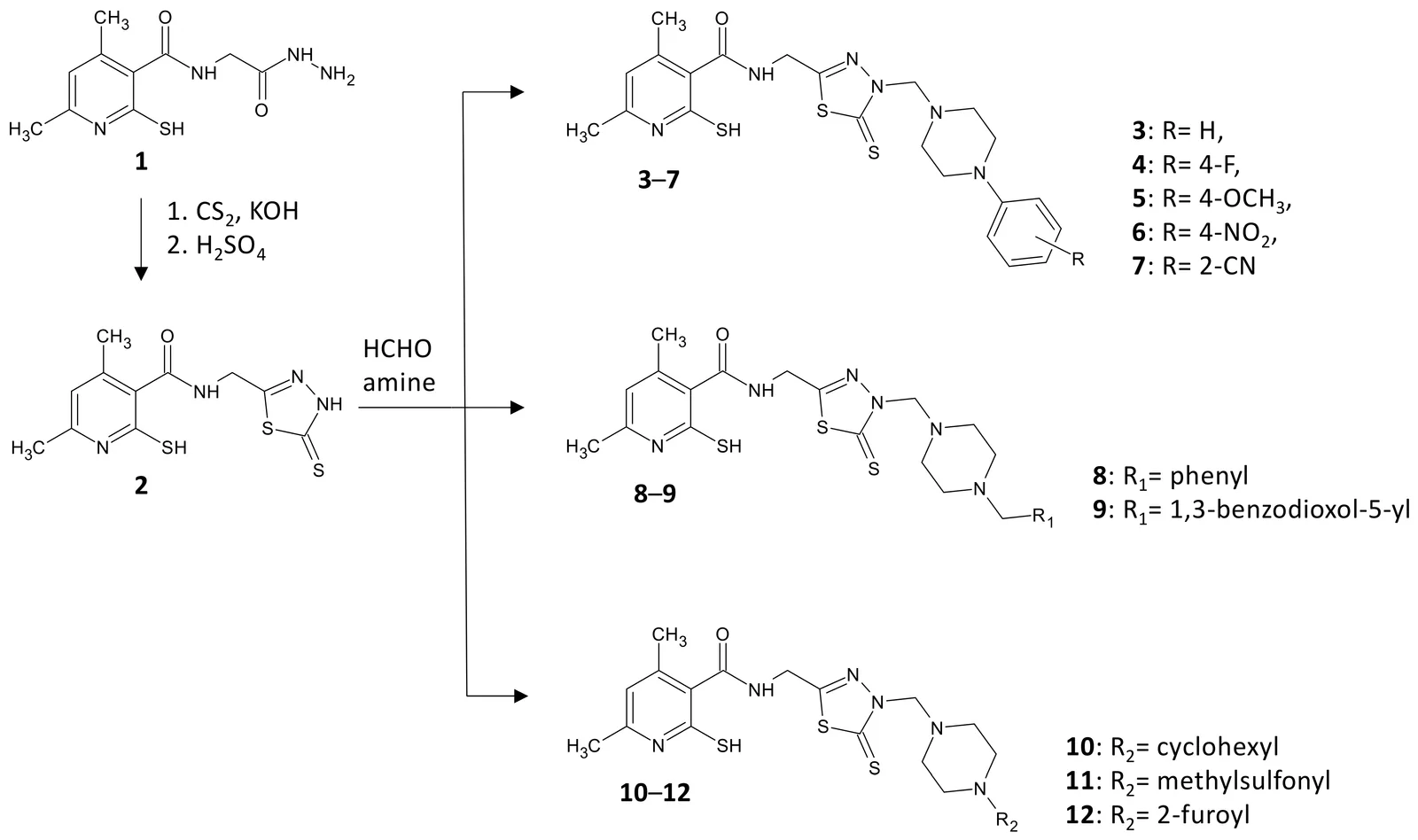
General procedure for preparation of target *N*-Mannich bases of pyridine-3-carboxamide–1,3,4-thiadiazole hybrids.

All the new compounds were prepared according to the synthetic pathways outlined in Scheme 1. The structures of compounds **2**–**12** were confirmed by FT-IR, ^1^H NMR, ^13^C NMR, and HR-MS, and all the analytical data are summarized in the experimental section and the Supplementary Materials. The starting material—*N*-(2-hydrazinyl-2-oxoethyl)-4,6-dimethyl-2-sulfanylpyridine-3-carboxamide **1**—was prepared according to the protocol published previously [35]. In the first step, acid hydrazide **1** was stirred with carbon disulfide in an ethanolic solution of potassium hydroxide at room temperature. The resulting potassium dithiocarbazate salt was subsequently cyclized by treatment with concentrated sulfuric acid at ice-bath temperature, affording the key intermediate—1,3,4-thiadiazole derivative **2**.

The FT-IR spectrum of compound **2** demonstrated broad bands at approximately 3463 cm^−1^ and 3252 cm^−1^ corresponding to NH groups, and other bands in the range of 1646–1583 cm^−1^ indicating the presence of C=O, C=N, and C=C fragments. In the ^1^H NMR spectrum, the disappearance of the characteristic NH and NH_2_ proton signals of the hydrazide moiety confirmed cyclization to the 1,3,4-thiadiazole ring. A triplet at δ 8.94 ppm integrating for one proton was assigned to the NH proton of the amide fragment, while singlets at δ 13.34 and 14.43 ppm corresponded to the SH proton of the pyridine ring and the NH proton of the thiadiazole ring, respectively. The ^13^C NMR spectrum confirmed the proposed structure. In particular, the resonances at δ 167.84 ppm and 189.39 ppm were attributed to the carbonyl carbon (C=O) and thiocarbonyl carbon (C=S) groups, respectively.

The final compounds **3**–**12** were obtained via a classical three-component Mannich reaction of compound **2**, containing an active hydrogen atom, formaldehyde, and the appropriate piperazine derivative in ethanol at room temperature.

The FT-IR spectra of the target *N*-Mannich bases showed the disappearance of the NH stretching band at approximately 3463 cm^−1^, confirming the substitution of the active NH proton of the thiadiazole ring during the Mannich reaction. The characteristic absorption bands attributed to the C=O, C=N, and C=C groups (1654–1583 cm^−1^) were preserved. In the ^1^H NMR spectra, the appearance of a two-proton singlet at δ 5.16–5.24 ppm, assigned to the methylene linker, confirmed the occurrence of the Mannich reaction and the attachment of the piperazine substituent to the thiadiazole ring. This was further supported by the disappearance of the NH proton signal of the thiadiazole ring. Moreover, signals corresponding to the piperazine ring protons were observed.

### 2.2. Biological Evaluation

In the first stage of biological evaluation, all tested compounds demonstrated measurable antimicrobial activity against the Gram-positive reference strain S. aureus ATCC 33591, with MIC values in a narrow range (0.034–0.069 mg/mL). Importantly, the MBC values were identical or very close to the corresponding MICs, indicating a bactericidal rather than merely bacteriostatic mode of action under planktonic conditions. In contrast, no detectable activity was observed against the Gram-negative strain *P. aeruginosa* ATCC 15542 or the yeast-like fungus C. albicans ATCC 10231 within the tested concentration range. This selective activity profile is notable, given that all strains used in this stage were pharmacopoeial reference strains (Table 1).

Despite the clear bactericidal effect observed against planktonic *S. aureus*, none of the tested compounds exhibited any measurable biofilm eradication activity. Biofilm reduction remained at 0% across all conditions, indicating a complete lack of efficacy against structured bacterial communities, even in the case of Gram-positive organisms.

For comparison, the assay usability control performed with an octenidine-based antiseptic showed MBC/MBEC values of 0.000244/0.003906 mg/mL for *S. aureus* ATCC 33591, 0.000488/0.015625 mg/mL for *P. aeruginosa* ATCC 15442, and 0.003906/0.03125 mg/mL for *C. albicans* ATCC 10231, respectively. Thus, the established octenidine-based antiseptic demonstrated substantially higher antimicrobial and antibiofilm activity than the newly synthesized compounds under the applied experimental conditions. Nevertheless, considering the continuing need to identify novel antimicrobial scaffolds and the possibility of further structure-based optimization, the selective activity of the investigated derivatives against Gram-positive bacteria remains relevant as a starting point for further development.

**Figure 3:**
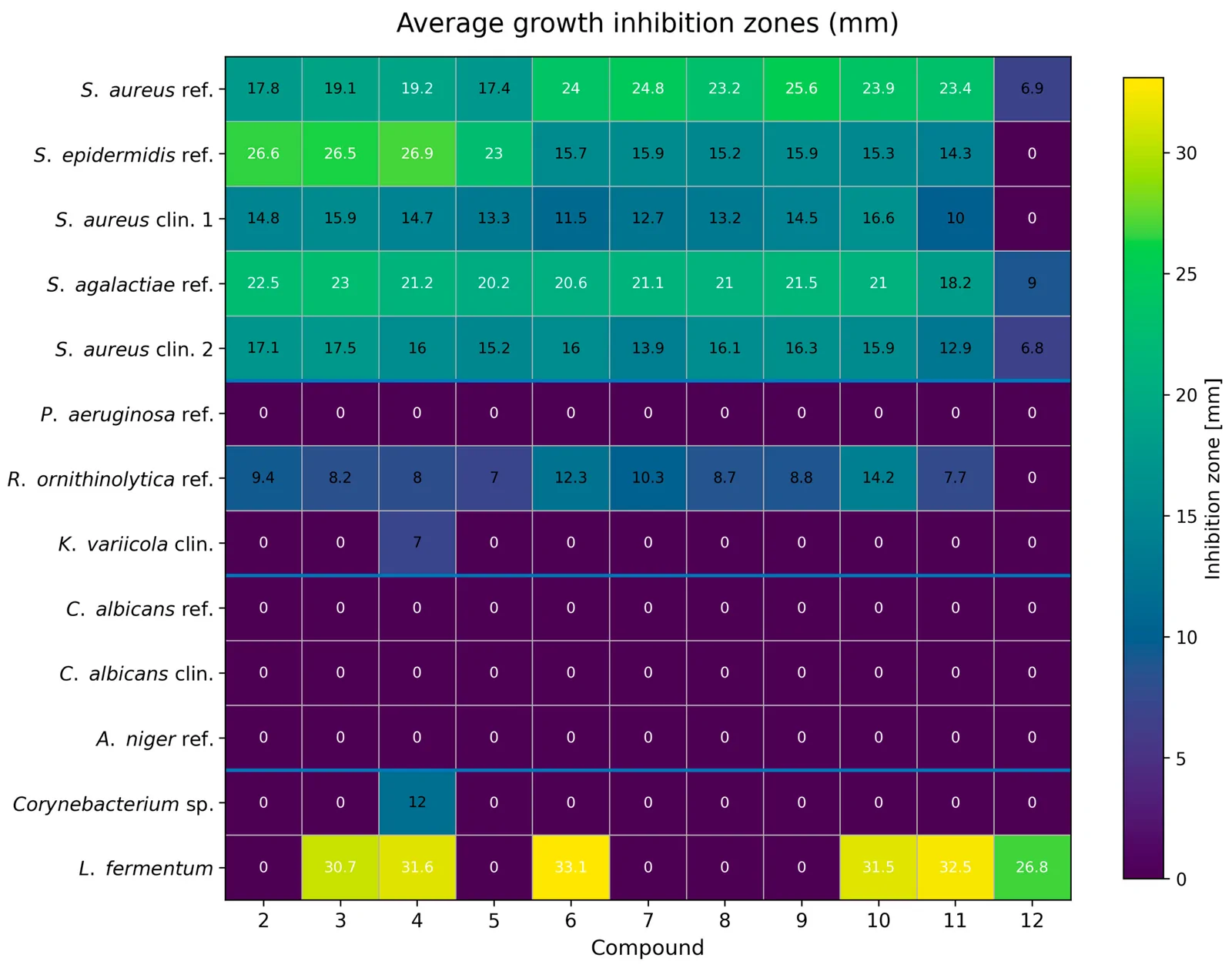
Heatmap showing the average growth inhibition zones (mm) of the tested microorganisms after exposure to the compounds investigated. The color scale represents the diameter of the growth inhibition zone, with the corresponding numerical value shown in each cell.

Considering the well-established intra- and inter-species variability in antimicrobial susceptibility, the experimental panel was expanded to include a broader range of reference and clinical strains representing Gram-positive and Gram-negative bacteria, fungi, and commensal Gram-positive microorganisms. At the same time, given the limited activity observed in the microtiter-based assays, particularly in terms of biofilm eradication, the analytical approach was adjusted to increase the sensitivity of detection. To this end, a modified agar diffusion (disk diffusion) method was employed, allowing for the identification of even moderate or localized antimicrobial effects that may not be captured under liquid-phase conditions. This complementary approach enabled a more comprehensive evaluation of the biological activity profile of the tested compounds across diverse microbial targets (Figure 3).

The activity of the tested compounds differed between microbial groups. For Gram-positive microorganisms, a measurable inhibitory effect was observed, with variability depending on both the compound and the species. *Staphylococcus* spp. showed moderate inhibition zones across most compounds, with noticeable variation among the tested derivatives. The strongest activity within this group was observed for compounds **7** and **9**. In contrast, *Streptococcus agalactiae* (strain **D**) exhibited a flat response profile across all compounds. Gram-positive *L. fermentum* displayed a distinct, highly selective pattern, with pronounced peaks of inhibition limited to the specific compounds **6**, **7**, **9**, **11**, and **12**. *Corynebacterium* sp. showed minimal susceptibility, with low and nearly unchanged inhibition zones across all tested compounds.

Among Gram-negative bacteria, a heterogeneous pattern was observed. *P.aeruginosa* remained unaffected, while *R. ornithinolytica* and *K. variicola* demonstrated moderate susceptibility, with measurable but limited inhibition that did not reach the levels observed for *Staphylococcus* spp.

Fungal strains, including *Candida albicans* (reference and clinical) and *Aspergillus niger*, showed no response to any compounds applied. The observed differences in susceptibility may reflect species-specific differences in cellular architecture and compound accessibility; however, the present experiments were not designed to determine the mechanisms underlying these patterns.

Together, these findings indicate that even within Gram-positive bacteria, structural and species-related differences can substantially influence the observed activity of the tested compounds. In parallel, the complete lack of activity observed for all fungal strains may be related to the fundamental differences in fungal cell wall composition, which is based on chitin and β-glucans rather than peptidoglycan, as well as the presence of ergosterol-containing membranes.

In contrast to Gram-positive microorganisms, Gram-negative bacteria exhibited a more heterogeneous response. *P. aeruginosa* remained largely unaffected across all tested compounds, showing consistently low inhibition. In comparison, *R. ornithinolytica* and *K. variicola* demonstrated moderate susceptibility, although the observed effects were clearly weaker than those recorded for *Staphylococcus* spp. This divergence within Gram-negative bacteria suggests that susceptibility is not uniform within this group and may depend on species-specific properties influencing compound access or activity.

Taken together, the present biological screening primarily defines the potential direction for further development of this new chemical scaffold. At this stage, the investigated derivatives should not be considered broad-spectrum stand-alone antimicrobial agents, particularly in view of their limited activity against biofilms and the substantially stronger activity of the reference antiseptic. Nevertheless, the observed antibacterial effects provide a rationale for further structure–activity optimization and mechanistic studies. An especially relevant future direction may be the evaluation of these compounds as potential adjuncts to established antibiotics or antiseptics, where even selective or moderate intrinsic activity could become valuable in combination-based antimicrobial strategies. Such an application, however, will require dedicated interaction and combination studies.

**Figure 4:**
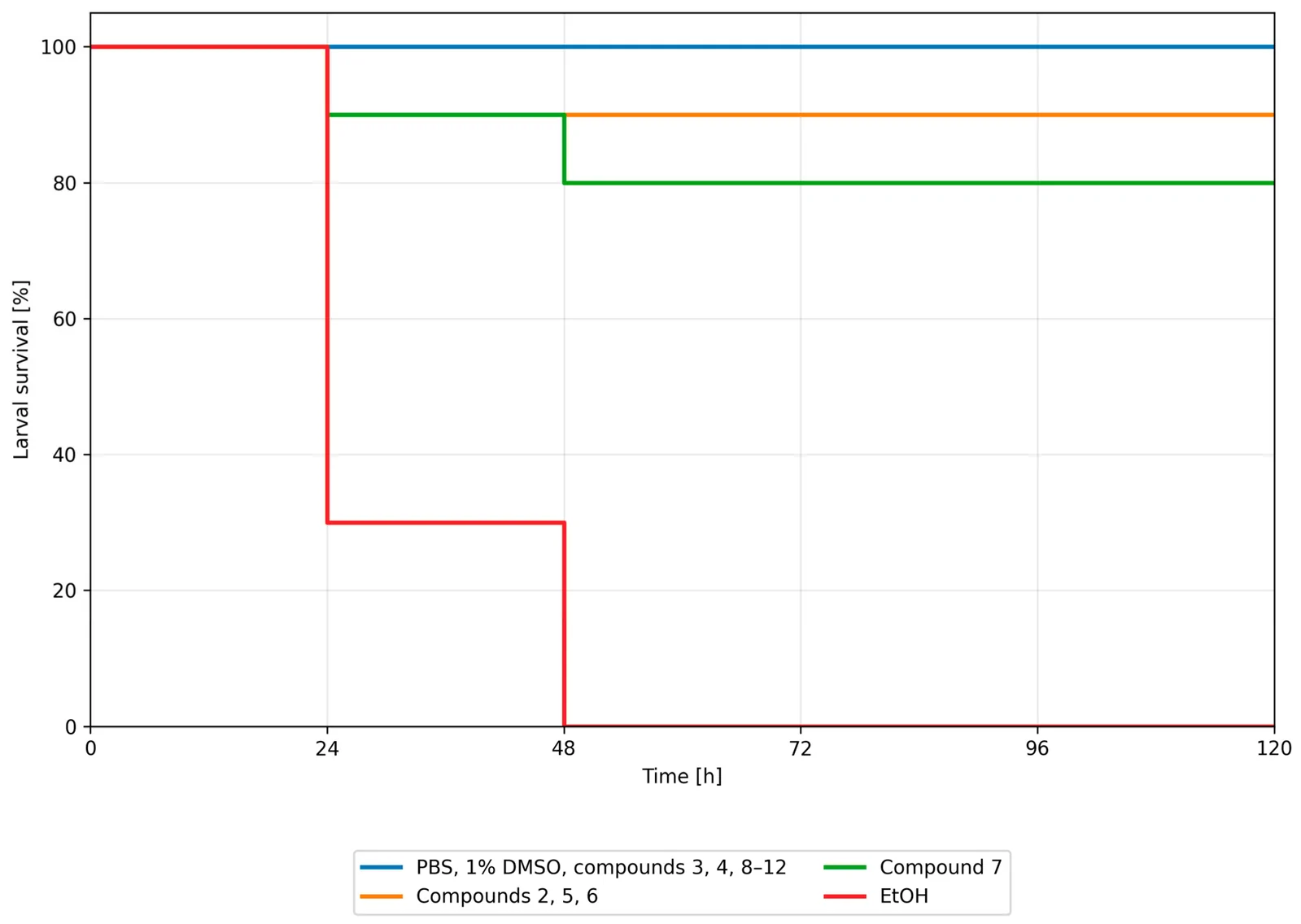
In vivo toxicity of compounds **2**–**12** measured by survival of larvae in *Galleria mellonella* model. Larval survival (n = 10; 10 = 100%) was monitored over 120 h.

Following the assessment of antimicrobial activity, the in vivo toxicity of the tested compounds was evaluated using the *Galleria mellonella* larval model over 120 h (5 days), where 10 larvae corresponded to 100% survival (Figure 4).

In the control group (Panel A), PBS and 1% DMSO showed no toxicity, with full survival (10/10) maintained throughout the experiment. In contrast, ethanol exhibited pronounced toxicity, causing a rapid decline in survival from 10/10 at 0 h to 3/10 at 24 h, and complete mortality (0/10) by 48 h, which persisted until the end of the observation period. Among the tested compounds (Panels B and C), the majority demonstrated no-to-low apparent acute toxicity under the applied experimental conditions. Compounds **3**, **4**, **8**, **9**, **10**, **11**, and **12** maintained full survival (10/10) across all time points, indicating no detectable adverse effects in vivo. Compounds **2**, **5**, and **6** showed a slight reduction in survival to 9/10 (90%), which remained stable over time. A more noticeable, yet still limited, effect was observed for **7**, where survival decreased to 8/10 (80%) within 48 h and remained unchanged thereafter. Although compound **7** is structurally distinct within the closely related **3**–**7** series due to the presence of an ortho-cyano substituent, the present single-dose experiment does not allow this structural feature to be causally associated with the observed mortality.

Overall, the tested compounds exhibited no-to-low in vivo toxicity, with survival profiles comparable to non-toxic controls and markedly distinct from the toxic ethanol condition.

### 2.3. Computational Chemistry and Molecular Modeling Study

**Figure 5:**
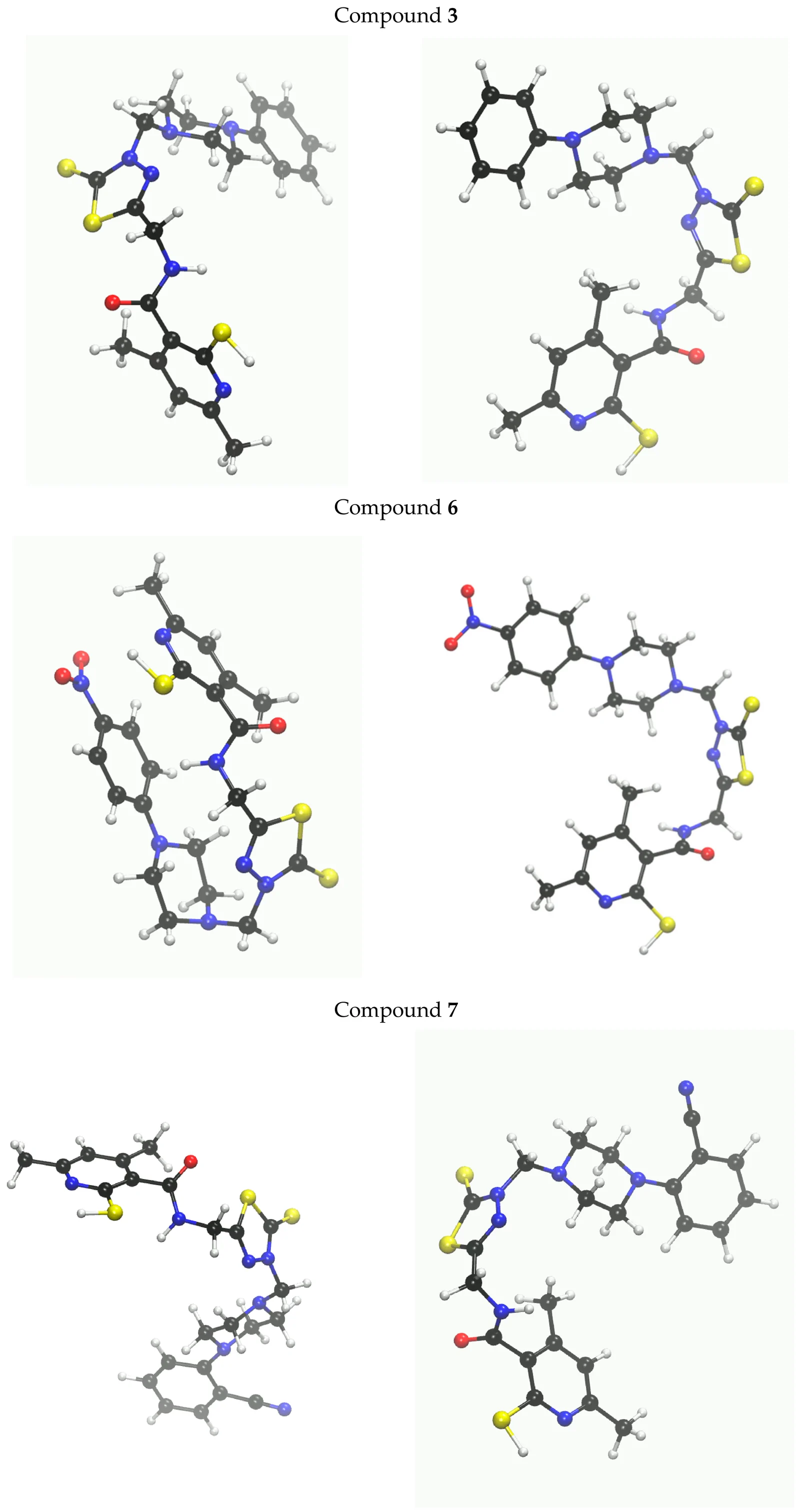
Optimized structures of the selected *N*-Mannich bases obtained as a result of DFT/ωB97XD/6-311+G(d,p) simulations in the gas phase and with solvent reaction field. Color coding: carbon—black, oxygen—red, nitrogen—blue, sulfur—yellow, hydrogen—white. The complete set of compounds is presented in the Supplementary Materials.

The DFT-based quantum-chemical simulations were performed in the gas phase and using the continuum solvation model, Integral Equation Formalism—Polarizable Continuum Model (IEF-PCM), to reproduce the polar environment’s influence on the spatial conformation of the studied set of compounds. The obtained structures, corresponding to the local minima located at the DFT/ωB97XD/6-311+G(d,p) level, are presented in Figure 5 and in Figure S1 in the Supplementary Materials. It was noticed that the introduction of the polar environment affected the spatial arrangement of atoms in the molecules, showing the ability of the optimization procedure to explore the potential energy surface of the studied compounds. It is particularly visible in compound **6**. The energy minimization in the gas phase provided us with a compact structure, which was sandwich-like; however, the presence of the polar environment prohibited some intramolecular interactions, and the obtained conformation is more “linear” (see Figure 5 and Figure S1). Concerning other structural changes, it is visible that the amide bond and the remaining part of the molecules rotated in the polar environment. More details are presented in Table S4, where the selected metric parameters (for the core part of the molecules; Scheme 1) are presented for compounds denoted as **3**, **6**, and **7**, because of their promising biological activity as well as molecular docking results (see text related to the discussion of ligand–host interactions). In addition, the optimized coordinates of the target compounds are presented in the Supplementary Materials to support our findings and conclusions.

Next, the partial atomic charge distribution obtained from the QTAIM analysis was investigated. The core fragment of the studied compounds is presented in Scheme S1 and was taken into consideration during the analysis, whereas the corresponding data for the three selected structures are summarized in Table S5.

**Figure 6:**
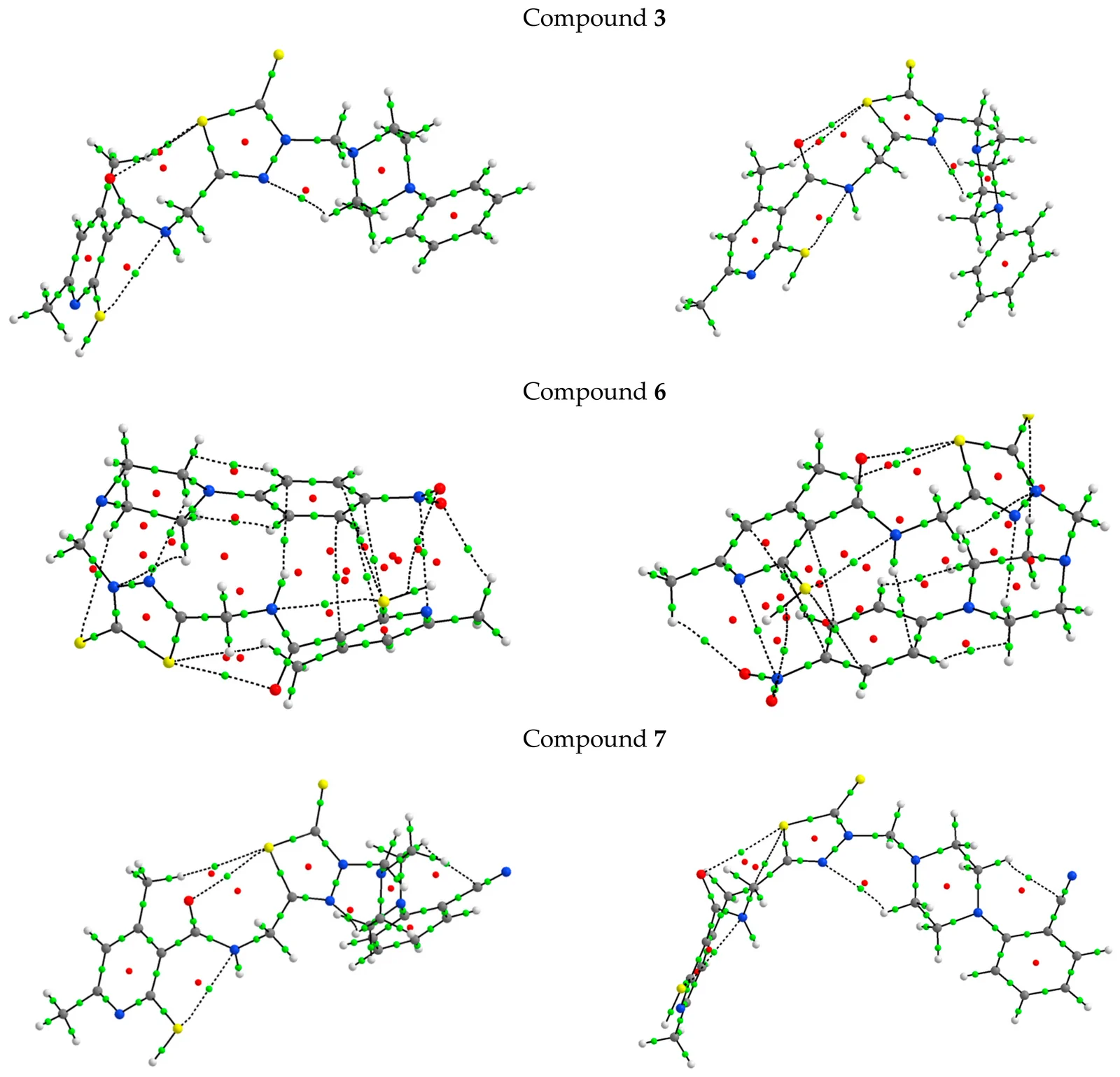
Selected QTAIM molecular graphs for compounds **3**, **6**, and **7** obtained based on results from the DFT/ωB97XD/def2-TZVP level of theory in the gas phase. The dotted line indicates the presence of a noncovalent interaction. The green and red spheres are Bond and Ring Critical Points (BCPs and RCPs) respectively. The complete set of the graphs is presented in Supplementary Materials.

Such an analysis may be useful for the future design of compounds with the desired biological activity, as it qualitatively demonstrates that the electron density distribution within the molecular core is influenced by the nature of the substituent. Quantitative changes in the electron density are also evident, and depending on the substituent, slight increases or decreases in the partial atomic charges were observed for the considered local minima. Besides the substituent effect, the introduction of the polar environment influenced the distribution of the electron density. It is especially visible for the partial atomic charges qC6, qC7, qC11, and qS3. In the core part of the molecules, the carbon atoms denoted as C6 and C7 are involved in the methyl groups, which serve as substituents in the pyridine ring. They are directly exposed to the polar environment influence. The carbon atom C11 is “shielded” because it is involved in the five-membered ring formation; however, the introduction of the polar environment increased the value of the partial atomic charge in all discussed compounds. The next significant influence on the environment on the partial atomic charge was noted for the sulfur atom S3. It is noted that the value of the partial atomic charge decreased compared to the gas-phase results. The sulfur atom (S3) acts as a substituent in the five-membered ring and is also affected by the polar environment, which is simulated using the IEF-PCM model and water as the solvent. The study is further supported by the electron density values at selected bond/ring critical points (B/RCPs) (see Table S6). As shown, the electron density values at the analyzed BCPs and RCPs are very similar for the investigated compounds, with only minor differences observed. Furthermore, molecular graphs derived from the QTAIM analysis are presented to visualize the intramolecular noncovalent interactions identified in the analyzed compounds (Figure 6 and Figure S2 and S3). In each structure, BCPs were identified between atoms connected by covalent bonds, while additional bond paths (represented by dotted lines) indicate the presence of intramolecular noncovalent interactions. This network of noncovalent interactions contributes to the structural stability of the molecules and results, among other features, in the formation of quasi-rings detected by the QTAIM analysis (see the corresponding figures).

For compound **6**, the network of intramolecular noncovalent interactions stabilizes the sandwich-like conformation in the gas phase that is visible in the obtained molecular graph based on QTAIM theory. The introduction of the polar environment did not preserve the above-mentioned structure—the model obtained after the geometry optimization with the solvent reaction field is more linear with a lower number of identified noncovalent interactions. However, this conformation is not preserved during molecular docking with the first molecular target (see Figure 6). A different situation is observed for the second guest–host complex, where the compound does not bind to the macromolecule in a fully extended (“linear”) conformation. These observations underline the structural flexibility of compound **6** (with 8 rotatable bonds). Its sandwich-like conformation stems from the presence of rings and nitro groups increasing the energetic preference for the stacking. However, introduction of the polar environment—either as water reproduced with the PCM model or as the presence of amino acid residues in the docking methodology—provides alternative contacts, which, considering the flexibility of the compound tuned by the presence of the environment, lead to increased linearity of the compound. We thus propose three structures which can play a role in the biological behavior of compound **6** depending on the nature of the environment.

To complete the description of molecular properties that are relevant in drug design, various molecular descriptors were computed with special attention paid to ADME. The obtained results are presented in Tables S8–S10 and in Figure S4. In Table S8, general physicochemical descriptors are presented, giving an idea of the chemical constitution, with, e.g., the number of heavy atoms, hydrogen bond donors/acceptors, rotatable bonds or molar refractivity. In Table S9, the lipophilicity and water solubility results are listed, whereas in Table S10, pharmacokinetics, druglikeness, and medicinal chemistry descriptors are presented. Concerning the solubility, the studied compounds were classified as mostly moderately or poorly soluble. The druglikeness was estimated based on various criteria, as implemented in the cheminformatics tool. As can be seen, some of the studied derivatives could be promising for further optimization. In Figure S4, the bioavailability radars are presented for each of the investigated derivatives. The bioavailability radar allows for a preliminary assessment of how similar a molecule is to a drug. Six physicochemical features are taken into consideration: lipophilicity, size, polarity, solubility, flexibility, and saturation. It is worth explaining that the pink area represents the optimal range for each of the analyzed properties. Concerning the set of the discussed compounds, according to the bioavailability radar, all of them are too polar, which may limit their potential for oral administration. In addition, some of the investigated compounds are not a suitable size.

Importantly, the relevance of this limitation depends on the intended route of administration. According to the antibacterial activity assay, compounds **2**–**12** inhibited the growth of *S. aureus*, with MIC values of 34–69 µg/mL, indicating measurable but moderate antibacterial activity. Their activity was substantially lower than that of the octenidine-based reference control used in the present study, indicating that these derivatives should be regarded as preliminary antimicrobial scaffolds rather than highly potent antibacterial agents. The mentioned coccus frequently colonizes the ear canals, nasopharynx, or skin. Consequently, the title compounds will likely find an application in treating such infections and would be administered topically in the form of ointments or drops. Therefore, their potential oral bioavailability and TPSA value may not be critical to their efficacy. However, if we want to obtain antibacterial agents that are effective after oral administration, we will probably have to optimize and modify the structures of derivatives **2**–**12** in order to reduce their TPSA values.

**Figure 7:**
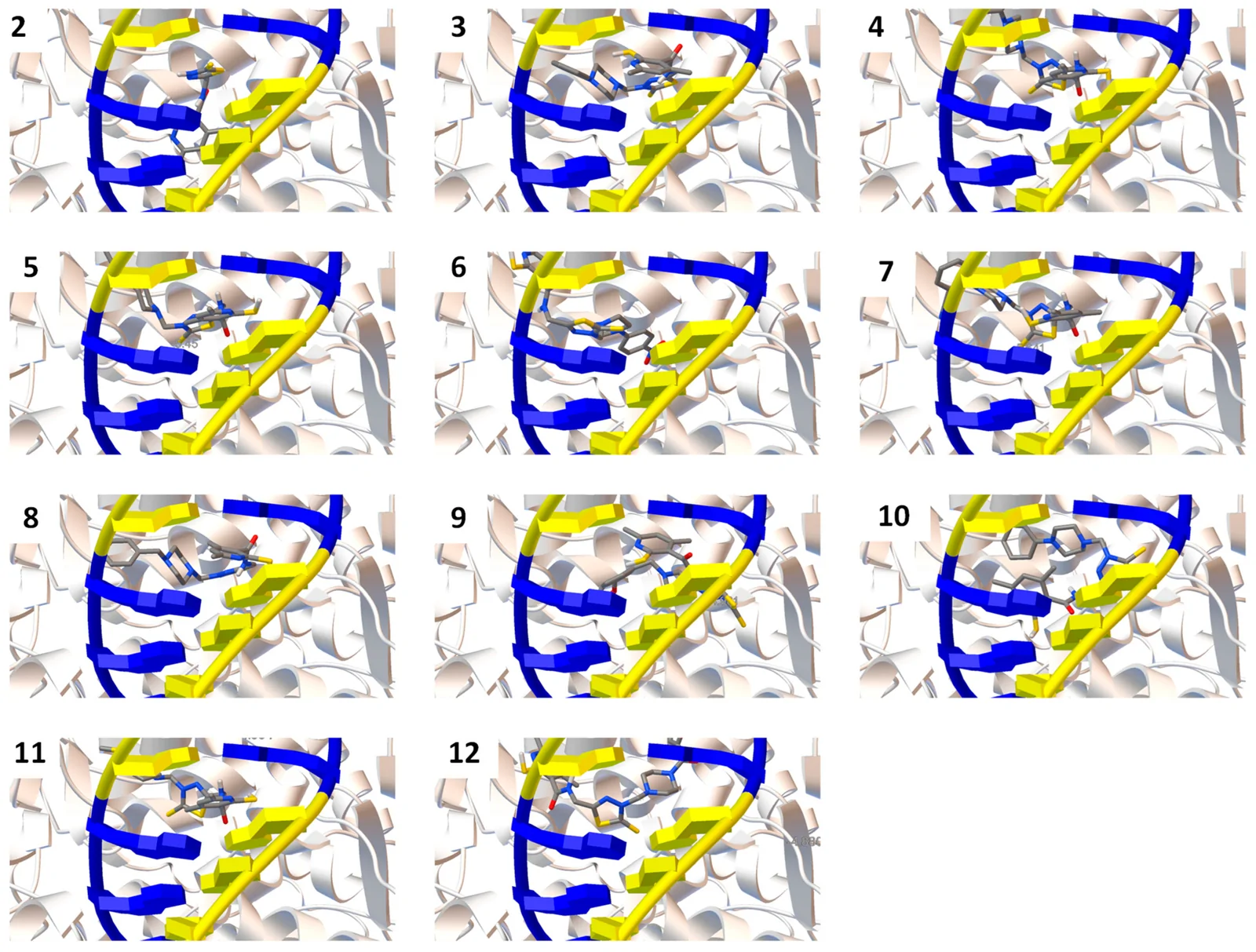
Results of molecular docking of compounds **2**–**12** to the *S. aureus* DNA gyrase. The poses overlapping the experimental position of the reference ligand present in the PDB 9N26 deposit, enabling the DNA intercalation, are shown. The protein and DNA are visualized as ribbons, while the ligands are in licorice representation.

**Figure 8:**
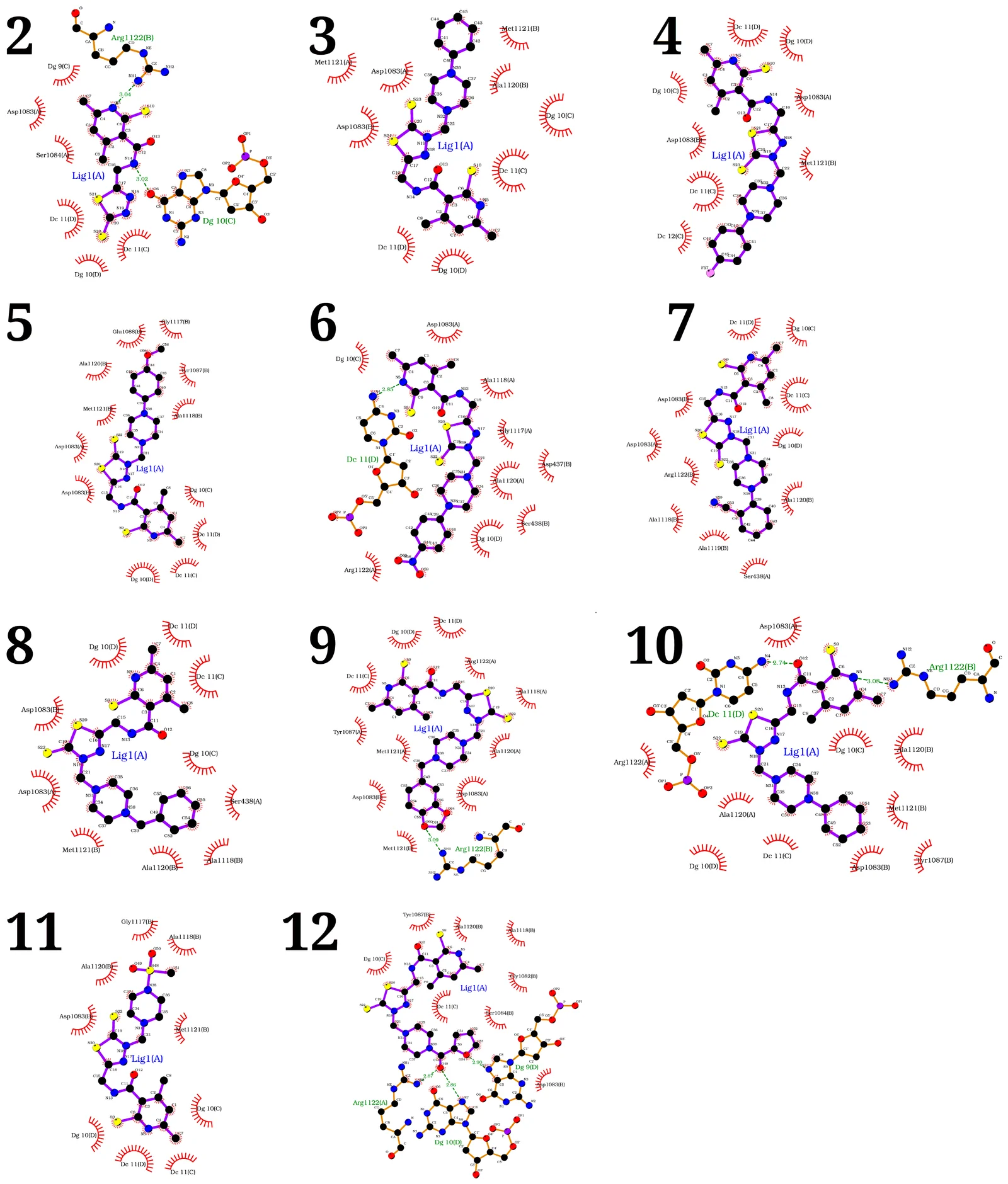
Host–ligand interaction maps resulting from the molecular docking of the **2**–**12** compounds to the *S. aureus* DNA gyrase. The poses overlapping the experimental position of the reference ligand present in the PDB 9N26 deposit, enabling the DNA intercalation, are shown.

Thiadiazole derivatives are an active object of investigation as antimicrobial targets with possible mechanism of action involving DNA topoisomerases [36,37]. The line of compounds under our study is similar to the one reported in the cited papers indicating tentative likeness of mechanism of action. Moreover, our idea was to propose possible molecular targets without excluding other possibilities. In an attempt to assign the tentative molecular targets of the studied compounds, molecular docking was carried out using two protein–DNA hosts: a *S. aureus* DNA gyrase (PDB code 9N26 [38]), and *E. coli* type Ia topoisomerase III (PDB code 2O54 [39]). The first PDB deposit contains a ligand intercalating into the double DNA helix, showing a possible mode of action. The second deposit contains a single DNA strand only, bound to the enzyme. Redocking of the original ligand into the *S. aureus* DNA gyrase provided the estimated binding affinity of −6.588 kcal/mol, with the ligand being placed in the original site of intercalation (see Figure S5 and its discussion in the Supplementary Materials). This value is an indicator of possible expected predictions for the investigated compounds. Docking results to the DNA gyrase are summarized in Table 2, Figure 7 and Figure 8.

Table 2 indicates the predicted affinity for the best pose as well as the pose overlapping the intercalation site (the latter is shown in Figure 7). The reporting of two poses is necessary because for most compounds, the best pose is in close contact with DNA present in the host, but it might not be located in the intercalation region. The estimated free energies of binding are ca. 1 kcal/mol worse than for the original ligand, but they are relatively consistent along the series. The compounds for which the best pose is indeed the same as for the reference ligand are: **5**, **7**, and **9**. The best compounds, selected on the basis of their affinity and docked geometry, are **6** and **7**, while the whole subset of **3**–**7** can be considered as promising. They feature somewhat smaller end groups than the remaining **8**–**12** compounds, indicating possible steric hindrances impairing the interactions with the DNA within the protein–DNA binding region.

While Figure 7 presents the three-dimensional location of the docked ligands, detailed mapping of the host–ligand interactions is presented in Figure 8 for the *S. aureus* DNA gyrase as the host. Highly consistent positioning of the ligands is visible in the fact that in all cases, the docked ligand forms hydrophobic interactions with the guanine and cytosine bases at the strand positions 10 and 11. Compounds **2**, **6**, **10**, and **12** also form hydrogen bonds with the bases, which does not increase the binding affinity significantly. This might seem surprising, but it is a result of desolvation—the experimental structure of the DNA gyrase, PDB ID 9N26, contains a large number of water molecules in contact with the DNA strand. Intercalation of the ligand requires removal of these water molecules with an energy penalty that is apparently similar to the energy gain due to the newly formed hydrogen bonds. Another important contact, present in all cases, is hydrophobic interaction with the Asp1083 residue—in most cases, this interaction covers two residues of the two chains, A and B, of the DNA gyrase. The twin residues are part of the system positioning the DNA strand, and their persistence in the interaction maps suggests the introduction of polar or charged groups into the skeleton of the ligands as one of the further directions of investigation. Finally, similar persistence of interactions is seen for the Arg1122 residues, which are frequently—but not always—present in the interaction maps, but in the case of **2**, **9**, **10** and **12**, this residue also forms hydrogen bonds with diverse parts of the ligands. This fact also supports the idea of introducing charged groups into the skeleton, which might affect the experimental properties (bioavailability).

**Figure 9:**
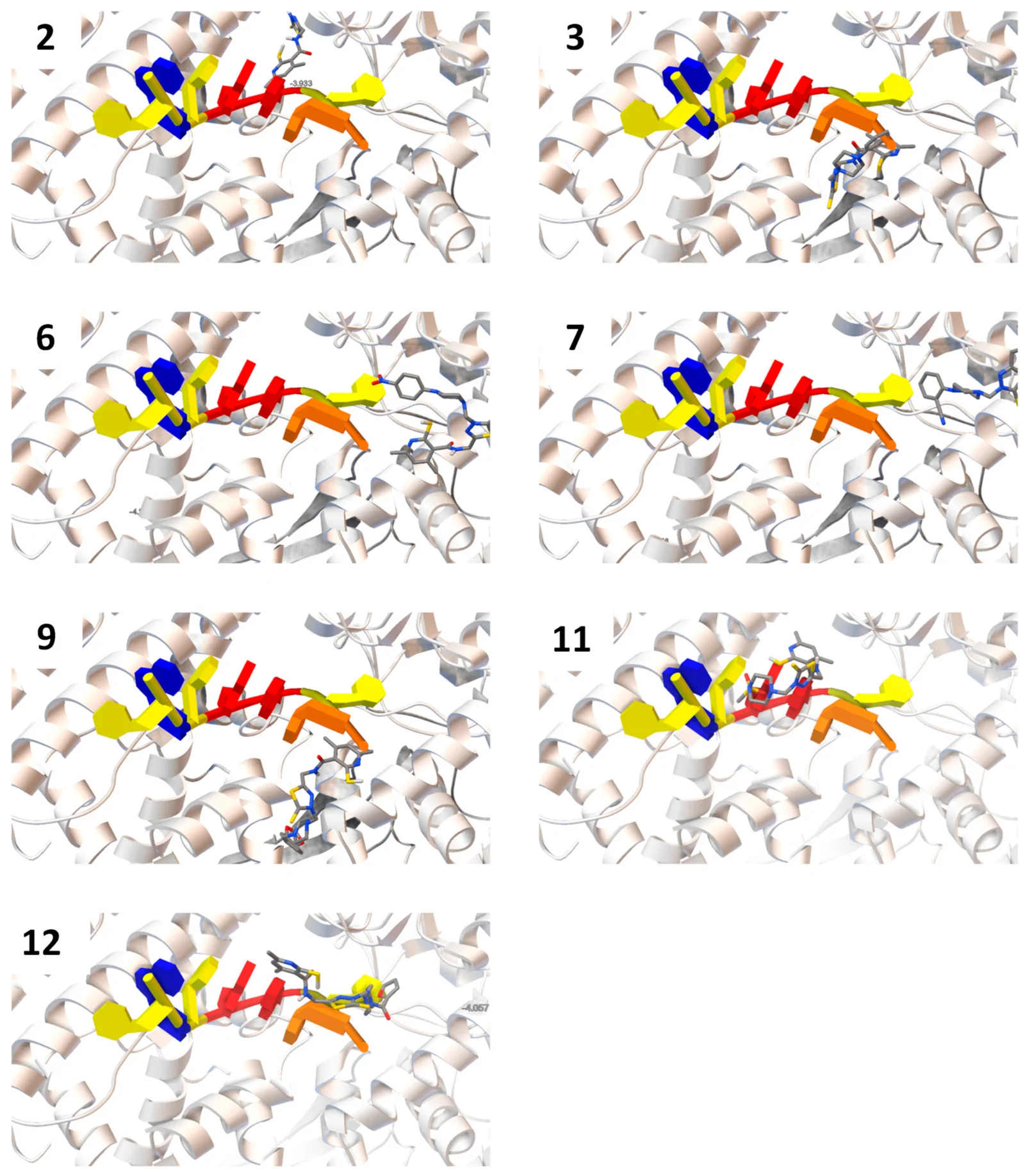
Results of molecular docking of the **2**–**12** compounds to the *E. coli* topoisomerase III type IA. Only the compounds and the poses in contact with the DNA strand present in the PDB 2O54 deposit are shown. The protein and DNA are visualized as ribbons, while the ligands are in licorice representation.

**Figure 10:**
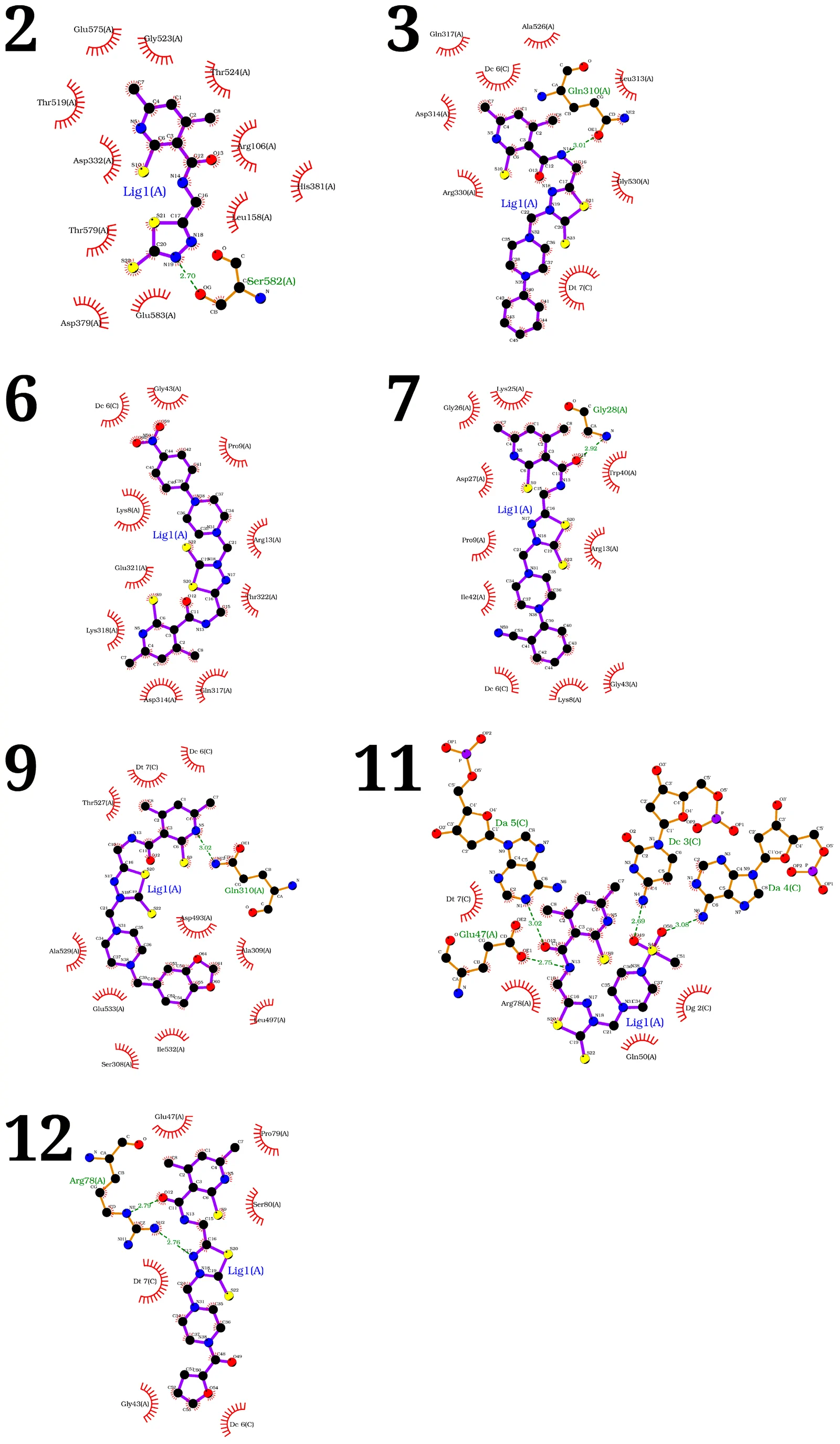
Host–ligand interaction maps resulting from the molecular docking of the **2**–**12** compounds to the *E. coli* topoisomerase III type IA. Only the compounds and the poses in contact with the DNA strand present in the PDB 2O54 deposit are shown.

Dockings to the second tentative molecular target, the *E. coli* type Ia topoisomerase III, are summarized in Table 3, Figure 9 and Figure 10.

This enzyme operates on a single strand, and the experimental deposit does not contain a ligand (inhibitor). The predicted affinities are significantly worse than for the first target, especially for the **3**–**7** subset. Moreover, the best poses are not in contact with DNA, with the exception of **9**. Even more significantly, for several compounds, none of the predicted binding poses are in contact with DNA. This suggests that the type Ia topoisomerase is a less promising molecular target than the DNA gyrase. The compounds worth further inquiry are **3**, **6**, and **7**. Importantly, this is a subset of the compounds suggested as the best ligands for the DNA gyrase, making them particularly worthy of detailed attention. However, Figure 9 shows that—in the absence of the second DNA strand—compounds **11** and **12** are also capable of close contact with the DNA octamer.

Figure 10 supports the initial observation that the topoisomerase III type IA is a less plausible molecular target than the DNA gyrase. The location of contacts with the DNA strand is not preserved within the series of ligands. In one case (compound **11**), as many as three hydrogen bonds are formed between the DNA bases and the ligand, which does not translate into an improved binding affinity. Ligands **2**, **3**, **7**, **9**, **11**, and **12** form hydrogen bonds with the protein, but the interactions are not consistent (only Gln310 appears twice in Figure 10). Forming any definite conclusions is therefore unlikely for this protein, especially when the approximate nature of the docking approach is considered.

Molecular docking supports DNA gyrase as the most plausible molecular target among the two enzymes investigated. In comparison with type IA topoisomerase III, the predicted binding affinities toward the DNA gyrase–DNA complex were consistently more favorable, and several compounds adopted binding modes closely resembling that of the co-crystallized ligand. Particularly noteworthy are compounds **6** and **7**, which combined favorable predicted binding energies with geometrically appropriate orientations within the DNA intercalation region. The agreement between docking predictions and the experimental antibacterial activity strengthens the hypothesis that inhibition of bacterial DNA gyrase may contribute to the selective activity against Gram-positive bacteria. It should be stressed at this point that docking protocols are very approximate in their nature. The scoring functions, including the Vinardo approach employed in this study, are very generic regarding atom typing, internal energy penalty caused by rotation around the rotatable bonds, and detection of hydrogen bonding. Small differences among the predicted binding affinities also call for caution in selecting the best ligands for each target. For this reason, we do not claim that the optimal molecular target was found; rather, we would like to point out the DNA gyrases as decidedly worthy of further investigations.

The docking results also provide preliminary structure–activity relationship information. The derivatives **3**–**7**, possessing relatively compact aromatic substituents attached to the piperazine ring, generally displayed more favorable docking scores than compounds **8**–**12**, which contain bulkier or more conformationally demanding substituents. This observation suggests that steric complementarity within the DNA-binding region of DNA gyrase plays an important role in ligand recognition. Larger substituents may experience steric clashes that reduce their ability to intercalate efficiently within the protein–DNA complex, despite retaining similar electronic properties.

## 3. Materials and Methods

### 3.1. Chemistry

#### 3.1.1. General Comments

All solvents and chemical reagents were purchased from commercial suppliers (Chemat, Gdańsk, Poland; Alchem, Wrocław, Poland) and were used without further purification. The progress of the reactions was monitored by thin-layer chromatography (TLC) on silica gel-coated aluminum sheets (Silica Gel 60 F254). The TLC plates were visualized under UV light at 254 and 365 nm. The melting points of all the new compounds were determined using the Electrothermal Mel-Temp 1101D apparatus (Cole-Parmer, Vernon Hills, IL, USA) with the open capillary method and were uncorrected. The ^1^H NMR (600 MHz) and ^13^C NMR (151 MHz) spectra were recorded on a Bruker NMR AVANCE III™ 600 MHz spectrometer (Bruker Analytische Messtechnik GmbH, Rheinstetten, Germany). The samples were dissolved in DMSO-*d*_6_, and tetramethylsilane (TMS) was used as an internal reference. Chemical shifts (δ) are reported in ppm, and multiplicities of NMR signals are designated as follows: s (singlet), d (doublet), t (triplet), q (quartet), and m (multiplet, for unresolved lines). To record and read spectra, TopSpin 5 (Bruker Analytische Messtechnik GmbH, Rheinstetten, Germany) was used. The infrared (IR) spectra were determined on the Nicolet iS50 FT-IR Spectrometer (Thermo Fisher Scientific, Waltham, MA, USA). The samples were applied as solids, and the frequencies were reported in cm^−1^. Spectra were read by OMNIC Spectra 2.0 (Thermo Fisher Scientific, Waltham, MA, USA). Mass spectra (MS) were recorded using the Bruker Daltonics Compact ESI-Mass Spectrometer (Bruker Daltonik, GmbH, Bremen, Germany), operating in the positive ion mode. The analyzed compounds were dissolved in a methanol/chloroform mixture. Theoretical monoisotopic masses of ions were calculated (calcd.) using Bruker Compass Data Analysis 4.2 software (Bruker Daltonik GmbH, Bremen, Germany).

#### 3.1.2. Preparation and Experimental Properties of Compounds **2**–**12**

##### Procedure for Preparation of Compound **2**

A mixture of 2 mmol of hydrazide **1** and 3 mmol of potassium hydroxide in 20 mL of ethanol was stirred for a few minutes. After dissolving the compounds, 2.6 mmol of carbon disulfide was added, and the mixture was stirred at room temperature for 5 h. The precipitate was filtered off and dried. To the resulting potassium dithiocarbazate salt, concentrated sulfuric acid was added dropwise until dissolution occurred; the mixture was kept in an ice bath and stirred for one hour. Afterward, ice was added to the flask, and the precipitated solid was filtered off and dried.

**2** 4,6-dimethyl-*N*-[(5-thioxo-4,5-dihydro-1,3,4-thiadiazol-2-yl)methyl]2-sulfanylpyridine-3-carboxamide

White solid, yield: 26%, m.p. 250–254 °C. ^1^H NMR (600 MHz, DMSO-*d*_6_) δ ppm: 14.43 (s, 1H, NH_thiadiazole_), 13.34 (s, 1H, SH), 8.94 (t, 1H, NH, *J* = 6 Hz), 6.52 (s, 1H, CH_pyridine_), 4.47 (d, 2H, CH_2_, *J* = 6 Hz), 2.29 (s, 3H, CH_3_), 2.07 (s, 3H, CH_3_). ^13^C NMR (151 MHz, DMSO-*d*_6_) δ ppm: 189.39, 174.41, 167.84, 162.36, 148.63, 146.58, 136.53, 115.28, 38.99, 19.52, 18.73. FT-IR (selected lines, γ_max_, cm^−1^): 3463, 3252 (N-H), 1646, 1624, 1583 (C=O, C=C, C=N). HR-MS (ESI-MS) (*m*/*z*): calcd. for C_11_H_12_N_4_OS_3_ [M+H]^+^: 313.0246; found: 313.0256.

##### General Procedure for Preparation of Compounds **3**–**12**

A total of 0.6 mmol of 36% formaldehyde was added to a solution of 0.5 mmol of 1,3,4-thiadiazole derivative **2** in 20 mL of ethanol. The obtained mixture was stirred at room temperature for several minutes. Then, 0.6 mmol of the appropriate piperazine derivative was added to the flask. The resulting mixture was stirred for 5 h at room temperature and then left overnight. The obtained precipitate was filtered off and crystallized from ethanol.

**3** 4,6-dimethyl-*N*-[(4-{[4-phenylpiperazin-1-yl]methyl}-5-thioxo-4,5-dihydro-1,3,4-thiadiazol-2-yl)methyl]-2-sulfanylpyridine-3-carboxamide

White solid, yield: 37%, m.p. 202–205 °C, decomp. (EtOH). ^1^H NMR (600 MHz, DMSO-*d*_6_) δ ppm: 13.34 (s, 1H, SH), 8.97 (t, 1H, NH, *J* = 6 Hz), 7.21–7.19 (m, 2H, ArH), 6.93–6.91 (m, 2H, ArH), 6.79–6.76 (m, 1H, ArH), 6.51 (s, 1H, CH_pyridine_), 5.25 (s, 2H, CH_2_), 4.53 (d, 2H, CH_2_, *J* = 6 Hz), 3.13–3.11 (m, 4H, CH_2 piperazine_), 2.89–2.88 (m, 4H, CH_2 piperazine_), 2.28 (s, 3H, CH_3_), 2.08 (s, 3H, CH_3_). ^13^C NMR (151 MHz, DMSO-*d*_6_) δ ppm: 188.65, 174.40, 167.91, 158.93, 151.51, 148.66, 146.61, 136.48, 129.37, 119.52, 116.15, 115.28, 70.32, 50.37, 48.81, 38.94, 19.55, 18.72. FT-IR (selected lines, γ_max_, cm^−1^): 3197 (NH), 1644, 1625, 1600 (C=O, C=C, C=N). HR-MS (ESI-MS) (*m*/*z*): calcd. for C_22_H_26_N_6_OS_3_ [M+H]^+^: 487.1403; found: 487.1412.

**4** 4,6-dimethyl-*N*-[(4-{[4-(4-fluorophenyl)piperazin-1-yl]methyl}-5-thioxo-4,5-dihydro-1,3,4-thiadiazol-2-yl)methyl]-2-sulfanylpyridine-3-carboxamide

White solid, yield: 34%, m.p. 195–197 °C decomp. (EtOH) ^1^H NMR (600 MHz, DMSO-*d*_6_) δ ppm: 13.34 (s, 1H, SH), 8.97 (t, 1H, NH, *J* = 6 Hz), 7.05–7.02 (m, 2H, ArH), 6.95–6.92 (m, 2H, ArH), 6.51 (s, 1H, CH_pyridine_), 5.24 (s, 2H, CH_2_), 4.53 (d, 2H, CH_2_, *J* = 6 Hz), 3.07–3.06 (m, 4H, CH_2 piperazine_), 2.89–2.87 (m, 4H, CH_2 piperazine_), 2.29 (s, 3H, CH_3_), 2.08 (s, 3H, CH_3_). ^13^C NMR (151 MHz, DMSO-*d*_6_) δ ppm: 188.64, 174.41, 167.92, 158.95, 157.36, 155.80, 148.67, 148.42, 146.61, 136.48, 117.96, 117.91, 115.78, 115.64, 115.28, 70.28, 50.35, 49.60, 38.95, 19.55, 18.72. FT-IR (selected lines, γ_max_, cm^−1^): 3162 (NH), 1643, 1625, 1586 (C=O, C=C, C=N). HR-MS (ESI-MS) (*m*/*z*): calcd. for C_22_H_25_FN_6_OS_3_ [M+H]^+^: 505.1309; found: 505.1325.

**5** 4,6-dimethyl-*N*-[(4-{[4-(4-metoxyphenyl)piperazin-1-yl]methyl}-5-thioxo-4,5-dihydro-1,3,4-thiadiazol-2-yl)methyl]-2-sulfanylpyridine-3-carboxamide

White solid, yield: 30%, m.p. 200–203 °C decomp. (EtOH) ^1^H NMR (600 MHz, DMSO-*d*_6_) δ ppm: 13.35 (s, 1H, SH), 8.98 (t, 1H, NH, *J* = 6 Hz), 6.95–6.91 (m, 2H, ArH), 6.87–6.86 (m, 2H, ArH), 6.52 (s, 1H, CH_pyridine_), 5.24 (s, 2H, CH_2_), 4.55 (d, 2H, CH_2_, *J* = 6 Hz), 3.75 (s, 3H, OCH_3_), 2.95–2.94 (m, 4H, CH_2 piperazine_), 2.89–2.87 (m, 4H, CH_2 piperazine_), 2.29 (s, 3H, CH_3_), 2.09 (s, 3H, CH_3_). ^13^C NMR (151 MHz, DMSO-*d*_6_) δ ppm: 188.65, 174.42, 167.91, 158.98, 152.41, 148.66, 146.62, 141.52, 136.51, 123.03, 121.20, 118.51, 115.28, 112.14, 70.54, 55.69, 50.63, 50.52, 38.96, 19.57, 18.73. FT-IR (selected lines, γ_max_, cm^−1^): 3186 (NH), 1644, 1625, 1585 (C=O, C=C, C=N). HR-MS (ESI-MS) (*m*/*z*): calcd. for C_23_H_28_N_6_O_2_S_3_ [M+H]^+^: 517.1509; found: 517.1498.

**6** 4,6-dimethyl-*N*-[(4-{[4-(4-nitrophenyl)piperazin-1-yl]methyl}-5-thioxo-4,5-dihydro-1,3,4-thiadiazol-2-yl)methyl]-2-sulfanylpyridine-3-carboxamide

White solid, yield: 27%, m.p. 228–230 °C decomp. (EtOH) ^1^H NMR (600 MHz, DMSO-*d*_6_) δ ppm: 13.33 (s, 1H, SH), 8.94 (t, 1H, NH, *J* = 6 Hz), 8.04 (d, 2H, ArH, *J* = 6 Hz), 7.03 (d, 2H, ArH, *J* = 12 Hz), 6.49 (s, 1H, CH_pyridine_), 5.26 (s, 2H, CH_2_), 4.50 (d, 2H, CH_2_, *J* = 6 Hz), 3.49–3.47 (m, 4H, CH_2 piperazine_), 2.87–2.86 (m, 4H, CH_2 piperazine_), 2.28 (s, 3H, CH_3_), 2.04 (s, 3H, CH_3_). ^13^C NMR (151 MHz, DMSO-*d*_6_) δ ppm: 188.68, 174.38, 167.89, 159.02, 155.07, 148.67, 146.59, 137.32, 136.46, 126.12, 115.25, 113.89, 113.11, 70.15, 49.96, 46.78, 38.92, 24.20, 19.52, 18.72. FT-IR (selected lines, γ_max_, cm^−1^): 3199 (NH), 1654, 1626, 1588 (C=O, C=C, C=N). HR-MS (ESI-MS) (*m*/*z*): calcd. for C_22_H_25_N_7_O_3_S_3_ [M+H]^+^: 532.1254; found: 532.1248.

**7** 4,6-dimethyl-*N*-[(4-{[4-(2-cyanophenyl)piperazin-1-yl]methyl}-5-thioxo-4,5-dihydro-1,3,4-thiadiazol-2-yl)methyl]-2-sulfanylpyridine-3-carboxamide

White solid, yield: 31%, m.p. 158–160 °C decomp. (EtOH) ^1^H NMR (600 MHz, DMSO-*d*_6_) δ ppm: 13.34 (s, 1H, SH), 8.99 (t, 1H, NH, *J* = 6 Hz), 7.70 (d, 1H, ArH, *J* = 6 Hz), 7.61–7.59 (m, 1H, ArH), 7.18 (d, 1H, ArH, *J* = 12 Hz), 7.12–7.09 (m, 1H, ArH), 6.51 (s, 1H, CH_pyridine_), 5.25 (s, 2H, CH_2_), 4.54 (d, 2H, CH_2_, *J* = 6 Hz), 3.15–3.14 (m, 4H, CH_2 piperazine_), 2.94–2.93 (m, 4H, CH_2 piperazine_), 2.29 (s, 3H, CH_3_), 2.08 (s, 3H, CH_3_). ^13^C NMR (151 MHz, DMSO-*d*_6_) δ ppm: 188.72, 174.39, 167.92, 159.14, 155.65, 148.66, 146.63, 136.49, 134.72, 122.66, 119.68, 118.68, 115.29, 105.27, 70.38, 51.65, 50.46, 38.95, 19.56, 18.72. FT-IR (selected lines, γ_max_, cm^−1^): 3187 (NH), 1645, 1626, 1584 (C=O, C=C, C=N). HR-MS (ESI-MS) (*m*/*z*): calcd. for C_23_H_25_N_7_OS_3_ [M+H]^+^: 512.1355; found: 512.1366.

**8** 4,6-dimethyl-*N*-[(4-{[4-benzylpiperazin-1-yl]methyl}-5-thioxo-4,5-dihydro-1,3,4-thiadiazol-2-yl)methyl]-2-sulfanylpyridine-3-carboxamide

White solid, yield: 32%, m.p. 177–181 °C decomp. (EtOH) ^1^H NMR (600 MHz, DMSO-*d*_6_) δ ppm: 13.34 (s, 1H, SH), 8.96 (t, 1H, NH, *J* = 6 Hz), 7.32–7.24 (m, 5H, ArH), 6.52 (s, 1H, CH_pyridine_), 5.16 (s, 2H, CH_2_), 4.52 (d, 2H, CH_2_, *J* = 12 Hz), 3.45 (s, 2H, CH_2 benzyl_), 2.75–2.73 (m, 4H, CH_2 piperazine_), 2.39–2.37 (m, 4H, CH_2 piperazine_), 2.29 (s, 3H, CH_3_), 2.08 (s, 3H, CH_3_). ^13^C NMR (151 MHz, DMSO-*d*_6_) δ ppm: 188.57, 174.41, 167.90, 158.85, 148.66, 146.60, 138.37, 136.51, 129.32, 128.63, 127.43, 115.28, 70.43, 62.40, 52.86, 50.28, 38.92, 19.55, 18.73. FT-IR (selected lines, γ_max_, cm^−1^): 3193 (NH), 1650, 1622, 1584 (C=O, C=C, C=N). HR-MS (ESI-MS) (*m*/*z*): calcd. for C_23_H_28_N_6_OS_3_ [M+H]^+^: 501.1559; found: 501.1551.

**9** 4,6-dimethyl-*N*-[(4-{[4-(1,3-benzodioxol-4-ylmethyl)piperazin-1-yl]methyl}-5-thioxo-4,5-dihydro-1,3,4-thiadiazol-2-yl)methyl]-2-sulfanylpyridine-3-carboxamide

White solid, yield: 26%, m.p. 180–186 °C decomp. (EtOH) ^1^H NMR (600 MHz, DMSO-*d*_6_) δ ppm: 13.35 (s, 1H, SH), 8.96 (t, 1H, NH, *J* = 6 Hz), 6.84–6.82 (m, 2H, ArH), 6.73 (d, 1H, ArH, *J* = 6 Hz), 6.52 (s, 1H, CH_pyridine_), 5.98 (s, 2H, CH_2 dioxole_), 5.16 (s, 2H, CH_2_), 4.52 (d, 2H, CH_2_, *J* = 6 Hz), 2.74–2.73 (m, 4H, CH_2 piperazine_), 2.37–2.35 (m, 4H, CH_2 piperazine_), 2.29 (s, 3H, CH_3_), 2.08 (s, 3H, CH_3_). ^13^C NMR (151 MHz, DMSO-*d*_6_) δ ppm: 188.57, 174.41, 167.88, 158.83, 148.66, 147.64, 146.59, 136.51, 132.09, 122.50, 115.27, 109.56, 108.29, 101.24, 70.42, 62.03, 52.68, 50.25, 38.91, 19.56, 18.73. FT-IR (selected lines, γ_max_, cm^−1^): 3177 (NH), 1650, 1624, 1585 (C=O, C=C, C=N). HR-MS (ESI-MS) (*m*/*z*): calcd. for C_24_H_28_N_6_O_3_S_3_ [M+H]^+^: 545.1458; found: 545.1450.

**10** 4,6-dimethyl-*N*-[(4-{[4-cyclohexylpiperazin-1-yl]methyl}-5-thioxo-4,5-dihydro-1,3,4-thiadiazol-2-yl)methyl]-2-sulfanylpyridine-3-carboxamide

White solid, yield: 38%, m.p. 225–232 °C decomp. (EtOH) ^1^H NMR (600 MHz, DMSO-*d*_6_) δ ppm: 13.34 (s, 1H, SH), 8.96 (t, 1H, NH, *J* = 6 Hz), 6.52 (s, 1H, CH_pyridine_), 5.16 (s, 2H, CH_2_), 4.52 (d, 2H, CH_2_, *J* = 6 Hz), 2.74–2.73 (m, 4H, CH_2 piperazine_), 2.54–2.52 (m, 4H, CH_2 piperazine_), 2.29 (s, 3H, CH_3_), 2.25 (s, 1H, CH_cyclohexyl_), 2.08 (s, 3H, CH_3_), 1.75–1.71 (m, 4H, CH_2 cyclohexyl_), 1.57–1.55 (m, 1H, CH_2 cyclohexyl_), 1.19–1.14 (m, 4H, CH_2 cyclohexyl),_ 1.07–1.06 (m, 1H, CH_2 cyclohexyl_). ^13^C NMR (151 MHz, DMSO-*d*_6_) δ ppm: 188.55, 174.42, 167.91, 158.89, 148.65, 146.59, 136.51, 115.27, 70.43, 63.19, 50.63, 48.68, 38.93, 28.57, 26.27, 25.70, 19.57, 18.72. FT-IR (selected lines, γ_max_, cm^−1^): 3168 (NH), 1650, 1625, 1585 (C=O, C=C, C=N). HR-MS (ESI-MS) (*m*/*z*): calcd. for C_22_H_32_N_6_OS_3_ [M+H]^+^: 493.1872; found: 493.1873.

**11** 4,6-dimethyl-*N*-[(4-{[4-(methylsulfonyl)piperazin-1-yl]methyl}-5-thioxo-4,5-dihydro-1,3,4-thiadiazol-2-yl)methyl]-2-sulfanylpyridine-3-carboxamide

White solid, yield: 40%, m.p. 222–226 °C decomp. (EtOH) ^1^H NMR (600 MHz, DMSO-*d*_6_) δ ppm: 13.35 (s, 1H, SH), 8.96 (t, 1H, NH, *J* = 6 Hz), 6.52 (s, 1H, CH_pyridine_), 5.22 (s, 2H, CH_2_), 4.52 (d, 2H, CH_2_, *J* = 6 Hz), 3.11–3.10 (m, 4H, CH_2 piperazine_), 2.87 (s, 3H, CH_3_), 2.84–2.83 (m, 4H, CH_2 piperazine_), 2.29 (s, 3H, CH_3_), 2.08 (s, 3H, CH_3_). ^13^C NMR (151 MHz, DMSO-*d*_6_) δ ppm: 188.65, 174.39, 167.88, 159.02, 158.73, 148.67, 147.34, 146.59, 145.14, 136.47, 116.19, 115.27, 111.76, 70.24, 50.61, 49.07, 38.89, 24.58, 19.52, 18.72. FT-IR (selected lines, γ_max_, cm^−1^): 3196 (NH), 1651, 1625, 1583 (C=O, C=C, C=N). HR-MS (ESI-MS) (*m*/*z*): calcd. for C_17_H_24_N_6_O_3_S_4_ [M+H]^+^: 489.0865; found: 489.0877.

**12** 4,6-dimethyl-*N*-[(4-{[4-(2-furoyl)piperazin-1-yl]methyl}-5-thioxo-4,5-dihydro-1,3,4-thiadiazol-2-yl)methyl]-2-sulfanylpyridine-3-carboxamide

White solid, yield: 34%, m.p. 205–211 °C decom. (EtOH) ^1^H NMR (600 MHz, DMSO-*d*_6_) δ ppm: 13.34 (s, 1H, SH), 8.94 (t, 1H, NH, *J* = 6 Hz), 7.83 (s, 1H, ArH), 6.98 (d, 1H, ArH, *J* = 6 Hz), 6.61 (s, 1H, ArH), 6.51 (s, 1H, CH_pyridine_), 5.23 (s, 2H, CH_2_), 4.51 (d, 2H, CH_2_, *J* = 6 Hz), 3.67–3.65 (m, 4H, CH_2 piperazine_), 2.80–2.78 (m, 4H, CH_2 piperazine_), 2.29 (s, 3H, CH_3_), 2.06 (s, 3H, CH_3_). ^13^C NMR (151 MHz, DMSO-*d*_6_) δ ppm: 188.65, 174.39, 167.88, 159.02, 158.73, 148.67, 147.34, 146.59, 145.15, 136.47, 116.19, 115.27, 111.76, 70.24, 50.56, 49.06, 38.89, 19.52, 18.72. FT-IR (selected lines, γ_max_, cm^−1^): 3190 (NH), 1647, 1606, 1586 (C=O, C=C, C=N). HR-MS (ESI-MS) (*m*/*z*): calcd. for C_21_H_24_N_6_O_3_S_3_ [M+H]^+^: 505.1145; found: 505.1144.

### 3.2. Biological Assessment

In the initial assessment, the following microbial strains were obtained from the American Type Culture Collection (ATCC, Manassas, VA, USA). The tested microorganisms included the Gram-positive bacterium *Staphylococcus aureus* MRSA 33591, the Gram-negative bacterium *Pseudomonas aeruginosa* 15442, and the yeast-like fungus *Candida albicans* 10231.

#### 3.2.1. Determination of Minimum Inhibitory Concentration (MIC)

1. Bacteria

The bacterial cultures were established in a Mueller–Hinton liquid medium (M-H). The bacterial suspension was adjusted to a turbidity equivalent to 0.5 McFarland standard and subsequently diluted 1:1000 in the M-H medium. The MIC assay was performed in a 96-well microtiter plate. The first well was left empty, while wells 2–10 in the row contained 100 μL of the liquid medium. Well 12 contained 200 μL of the medium, serving as an additional control. Well 11 was supplemented with 100 μL of antimicrobial solution. The tested compound was added to well 1 at a volume of 200 μL. The highest final concentration of the tested compounds in the plate was 0.55 mg/mL. MIC and MBC determinations were performed using serial two-fold dilutions.

The excess 100 μL from well 9 was discarded. Next, 100 μL of bacterial suspension of 10^5^ colony forming units/mL was added to wells 1–11 in the first three rows. A control row for the tested compound was included, where wells 1–9 contained 100 μL of the medium, and wells 10 and 11 contained 100 μL of the bacterial suspension. The highest concentration of compounds in the plate was 0.5 mg/mL. The plate was incubated for 24 h at 37 °C under shaking conditions. Subsequently, 20 μL of 1% triphenyltetrazolium chloride (TTC) was added to each well, followed by an additional 2 h incubation at 37 °C with shaking. The lowest concentration of the tested compound that prevented color change was considered the MIC. Optical density was measured at 490 nm using a spectrophotometer.

2. Yeast-like fungus

An analogical protocol was followed for *C. albicans*, except that the initial cell density was adjusted to 2.0 McFarland standard before 1:1000 dilution in the liquid medium. The serial dilution setup mirrored that used for bacteria, and the final viability indicator was 0.01% resazurin, incubated for 3 h at 37 °C. The MIC was determined as the lowest concentration that prevented resazurin reduction, with spectrophotometric readings recorded at 570 nm and 600 nm.

#### 3.2.2. Determination of Minimum Bactericidal Concentration (MBC)

Following MIC determination, the MBC was assessed for both bacteria and fungi. Wells corresponding to MIC, two preceding wells, and two subsequent wells were selected for further analysis. Each selected well (200 μL) was transferred to a fresh set of wells containing 2.3 mL of fresh medium (except for the last well, which contained 2.5 mL). The plates were incubated for 24 h at 37 °C, and bacterial growth was visually assessed to determine the MBC.

#### 3.2.3. Determination of Minimum Biofilm Eradication Concentration (MBEC)

1. Bacteria

Fresh bacterial cultures were prepared and adjusted to a turbidity of 0.5 McFarland before 1:1000 dilution. Wells 1–10 of a 96-well plate were inoculated with 200 μL of bacterial suspension. Well 11 contained 100 μL of the selected antiseptic and 100 μL of bacterial suspension, while well 12 contained 200 μL of the medium. Plates were incubated at 37 °C for 24 h to allow biofilm formation. After incubation, the medium was carefully removed from wells 1–9 without disturbing the biofilm. A separate microtiter plate was prepared for serial dilutions of the tested compound. The highest concentration of compounds in plate was 1mg/mL. The first well remained empty, and wells 2–9 contained 100 μL of the selected medium. The tested compound was added to well 1 (200 μL), and serial dilutions were performed identically to the MIC assay. The diluted solutions were then carefully transferred onto the preformed biofilm-containing plate, followed by a 24 h incubation at 37 °C. After incubation, the medium was removed without disturbing the biofilm. Biofilm viability was assessed by adding 200 μL of 0.1% TTC to all wells, incubating for 2 h at 37 °C. The stained biofilms were subsequently treated with 200 μL of a methanol-acetic acid (9:1) solution, shaken for 0.5–2 h, and the contents were transferred to a clean plate. The absorbance was measured at 490 nm. An untreated biofilm control was included in each experiment. The absorbance value obtained for this control was considered to represent 100% biofilm viability, and the relative viability of bio-films exposed to the tested compounds was calculated with reference to this value.

2. Yeast-like fungus

The methodology followed for *C. albicans* was identical to that for bacteria, except for initial cell density adjustments (2.0 McFarland standard before 1:1000 dilution) and the use of 0.001% resazurin as a biofilm viability indicator. Resazurin-treated wells were incubated for 3 h at 37 °C, and spectrophotometric measurements were recorded at 570 nm and 600 nm.

For all three reference strains, an assay usability control was performed using the octenidine-based antiseptic Octenisept^®^ (Schülke & Mayr GmbH, Norderstedt, Germany), which was included as a reference antimicrobial agent with established antimicrobial and antibiofilm activity.

#### 3.2.4. Extended Antimicrobial Screening (Agar Diffusion Assay)

Following the initial quantitative assessment performed on pharmacopoeial reference strains (Section 3.2.1, Section 3.2.2 and Section 3.2.3), the antimicrobial spectrum of the tested compounds was further evaluated using an agar diffusion-based screening approach, selected to enable detection of even moderate antimicrobial effects across a broader panel of microorganisms. The analysis included both reference and clinical strains representing Gram-positive and Gram-negative bacteria, fungi, and commensal species. The clinical isolates applied are part of The Strain Collection of Department of Translative Technologies of Wroclaw Medical University. Gram-positive bacteria comprised *S. aureus* ATCC 25923, *S. epidermidis* (non-biofilm-forming strain), and clinical biofilm-forming *S. aureus* isolates (b2 and b5), as well as *Streptococcus agalactiae* ATCC 13813. Gram-negative bacteria included *P. aeruginosa* ATCC 15442, a clinical *Klebsiella pneumoniae* isolate, and *Raoultella ornithinolytica* DSM 7464. Fungal strains included *C. albicans* ATCC 10231 and a clinical isolate, as well as the mold *Aspergillus niger* 16404. Additionally, a non-pathogenic Gram-positive environmental *Lactobacillus fermentum* strain and Gram-positive *Corynebacterium* spp., both clinical isolates, were included. Microbial suspensions were adjusted to 0.5 McFarland and inoculated onto Mueller–Hinton agar (M173, Himedia) or Sabouraud agar, depending on the organism. Sterile paper disks (Himedia Laboratories Pvt. Limited, Maharashtra, India) were loaded with 20 µL of the tested compounds (10 mg/mL) and placed onto inoculated agar plates. Plates were incubated at 37 °C for 24 h for bacteria and yeasts, and up to 14 days for *A. niger*. Zones of growth inhibition were measured in millimeters. In the case of *A. niger*, additional qualitative assessment of conidia maturation was performed. All biofilm-forming strains included in this stage had been previously characterized as pox-positive. For *Corynebacterium*, a modified protocol was applied. Briefly, 30 µL of the compound (10 mg/mL) was applied onto 5.5 mm diameter disks, allowed to dry (DMSO solvent evaporation), and placed onto nutrient agar (Biolife Italiana S.r.l., Monza, Italy) supplemented with 3% erythromass. Plates were inoculated with a 0.5 McFarland suspension and incubated at 37 °C for 24–48 h. Pure DMSO served as a control. Experiments were performed in duplicate, with a standard deviation of ±0.7 mm.

#### 3.2.5. Determination of Toxicity In Vivo Using Galleria mellonella Larval Model

All experiments were performed using *Galleria mellonella* larvae obtained from an in-house breeding maintained at the Department of Translative Technologies of Wroclaw Medical University. Larvae at the final instar stage, weighing 200–300 mg, and showing no signs of melanization or physical damage were selected for the study. Each larva was injected with 10 µL of the tested solution (2 mg/mL) using a calibrated microsyringe (Hamilton Company, Reno, NV, USA) fitted with a fine-gauge needle, via the last left proleg. For each compound, a group of 10 larvae was used. Control groups included larvae injected with 10 µL of 90% ethanol (positive control for cytotoxicity) and 10 µL of sterile phosphate-buffered saline (PBS; negative control). Following injection, larvae were incubated under controlled conditions (in the dark, at 30 °C) and monitored over a period of 5 days. Larval survival was recorded daily. In addition, qualitative assessment of phenotypic changes was performed, with particular emphasis on alterations in body coloration (melanization), which served as an indicator of physiological response to the tested compounds.

#### 3.2.6. Data Analysis

The biological experiments were performed in three independent experimental runs. MIC, MBC, and MBEC values were determined independently in each run using serial two-fold dilutions. The Galleria mellonella survival experiment was likewise performed in three independent runs, with 3, 3, and 4 larvae per treatment group in the respective experiments, giving a total of n = 10 larvae per treatment condition. Larval survival data are presented as the pooled survival of these 10 larvae over the 120 h observation period.

### 3.3. Computational Methodology

A database containing 11 compounds was constructed using the graphical module of Avogadro 1.2.0 version [40]. Subsequently, the obtained models were subjected to an initial energy minimization based on Molecular Mechanics using the MMFF94 force field [41], implemented in the same software. Next, Density Functional Theory (DFT) [42,43] was employed for the final geometry optimization and harmonic frequency calculations. No additional conformational search was performed; therefore, the structures presented in the results and discussion should be regarded as local minima. The energy minimum was obtained based on the Berny geometry optimization algorithm [44] implemented in the Gaussian 16 Rev. C.0.2. package [45]. The simulations were performed in the gas phase as well as with the solvent reaction field reproduced by the application of the continuum solvation model (IEF-PCM) [46,47] and water as a solvent. No imaginary frequencies were noticed, confirming that the structures correspond to energy minima on the Potential Energy Surface (PES). The ωB97XD functional [48] and Pople’s type 6-311+G(d,p) basis set [49,50,51] were used in this part of the simulations. The XYZ coordinates are provided in the Supplementary Materials file. The choice of the level of theory was dictated by the fact that the investigated compounds exhibit high flexibility, which may result in the formation of intramolecular noncovalent interactions that stabilize the molecular conformation. Therefore, the functional with dispersive forces as well as the basis set with polarization and diffuse functions were applied to accurately describe the set of compounds under study. This level of theory has already been applied in theoretical studies of potentially biological relevant compounds, e.g., [52,53]. In addition, the DFT part with proposed electronic structure analysis is in line with studies where biologically active compounds were analyzed [54,55,56].

In the next step, wave functions were generated for further electron density analyses based on the same level of theory. The Gaussian 16 Rev. C.0.2. suite of programs [45] was employed for the calculations. The Quantum Theory of Atoms in Molecules (QTAIM) [57] analysis was performed to detect changes in the electronic structure resulting from the introduction of various structural fragments into a core part of the studied compounds. The partial atomic charges, as well as the values of the electron density (ρ) at bond and ring critical points (BCPs and RCPs) were taken into consideration to provide a quantitative description of the structure modification’s influence on the molecular features. In addition, the theory was used to detect noncovalent interactions present in the set of the studied compounds. This part of the analysis was done using the AIMAll program [58]. In order to develop significant descriptors from the drug design point of view, the SwissADME server was used [59]. Finally, the molecular docking [60] was conducted to estimate the strength and type of interactions between the guest and the host molecules, as well as the ligand conformation. As molecular targets for the studied compounds, the DNA gyrase of *S. aureus* (PDB code: 9N26) and type IA topoisomerase III of *E. coli* (PDB code: 2O54) were considered [38,39] and their structures were extracted from the Protein Data Bank (PDB) [61]. The macromolecules and small molecules were prepared for the molecular docking according to the procedure implemented in the AutoDockTools 1.5.7 [62]. The semi-rigid docking procedure was applied for the cases studied. In the procedure, the macromolecule geometry is preserved, while the ligand molecule exhibits flexibility. Concerning the macromolecules, water molecules and other co-factors were removed, while the protein and DNA strand were retained. The Kollman atomic charges [63] and polar hydrogen atoms were added to the considered receptors. The parts of the macromolecules which could possibly interact with the set of the studied small molecules were inferred from the knowledge of the center of co-crystallized ligands or DNA strands. Therefore, the grid was set to the dimensions of 60 × 60 × 60 Å for both molecular targets. The ligands for the molecular docking examination were prepared based on the DFT-geometry. The Gasteiger atomic charges [64] were assigned on each atom of the molecules as well as polar hydrogens. The AutoDockVina 1.2.7 and Vinardo scoring function [65,66] were used for the procedure as well as to detect the ligand–receptor binding affinity. The interactions in the binding site were visualized using the AutoDockTools package [62]. Additionally, the graphical presentation of the results was prepared with the assistance of the VMD [67] and LigPlot+ 2.2.9 [68] programs.

## 4. Conclusions

In the present study, a new series of *N*-Mannich bases of pyridine-3-carboxamide–1,3,4-thiadiazole hybrids was designed, synthesized, and comprehensively characterized using FT-IR, ^1^H/^13^C NMR, and HR-MS techniques. The synthetic approach enabled the efficient preparation of the target compounds and provided a structurally diverse set of derivatives for biological and computational evaluation. The synthesized compounds exhibited selective antimicrobial activity against the Gram-positive reference strain *Staphylococcus aureus* ATCC 33591, with MIC values ranging from 0.034 to 0.069 mg/mL and MBC values comparable to the corresponding MICs, indicating a bactericidal effect under planktonic conditions. In contrast, no significant activity was observed against *Pseudomonas aeruginosa* ATCC 15542 or *Candida albicans* ATCC 10231, and none of the compounds showed measurable activity against preformed *S. aureus* biofilms. The complementary agar diffusion experiments further supported the selective antimicrobial potential of the investigated derivatives and provided additional information on their activity against a broader panel of microorganisms.

The *Galleria mellonella* model indicated low acute toxicity of the majority of the tested compounds. Most derivatives did not affect larval survival under the applied experimental conditions, supporting their further investigation as potential antimicrobial scaffolds. Nevertheless, these preliminary toxicity data should be regarded as an initial safety assessment and require confirmation using additional toxicological models.

Computational studies provided insights into the structural and electronic properties of the investigated compounds. DFT and QTAIM analyses demonstrated that both the nature of the substituent and the environment can influence molecular conformation and electron-density distribution. In particular, the local minimum obtained at the DFT level for compound **6** exhibited a distinct sandwich-like conformation in the gas phase, stabilized by a network of intramolecular noncovalent interactions. The introduction of a polar environment resulted in a switch to a new computational model with another local minimum—a more extended conformation and changes in selected atomic charge distributions. Molecular descriptor and ADME analyses indicated that several compounds possess physicochemical properties that may be more compatible with topical than oral administration, although further structural optimization would be required to improve their drug-like properties where appropriate.

Molecular docking identified DNA gyrase as a putative molecular target worthy of further investigation. Several derivatives, particularly **6** and **7**, adopted binding modes compatible with the DNA intercalation region and showed relatively favorable predicted binding affinities. In comparison, docking to *E. coli* type IA topoisomerase III produced less consistent binding modes and generally less favorable predicted affinities. The docking results therefore suggest that DNA gyrase may contribute to the antibacterial activity of the investigated compounds; however, these computational findings do not constitute experimental evidence of target engagement. Further biochemical and biophysical studies will be necessary to confirm DNA gyrase inhibition and to establish the precise molecular mechanism of action.

Overall, the results demonstrate that *N*-Mannich bases of pyridine-3-carboxamide–1,3,4-thiadiazole hybrids represent promising starting points for the development of new antimicrobial scaffolds, particularly against Gram-positive bacteria. Compounds **6** and **7** emerged as the most interesting candidates for further optimization based on the combination of antimicrobial activity, low apparent in vivo toxicity, physicochemical characteristics, and computational analysis. Future studies should focus on systematic structure–activity relationship optimization, confirmation of the proposed molecular targets using experimental assays, evaluation against a broader panel of clinically relevant strains, and further characterization of the pharmacological and safety profiles of the most promising derivatives.

## Figures and Tables

**Table 1:** Antimicrobial and antibiofilm activity of compounds **2**–**12** and the octenidine-based reference control against selected reference microorganisms.

Species	Gram-Positive			Gram-Negative			Yeast-like Fungus		
	*S. aureus* ATCC *6538*			*P. aeruginosa* ATCC *14452*			*C. albicans* ATCC *10231*		
**Compound**	**MIC** ^a^	**MBC** ^a^	**Biofilm Reduction**	**MIC** ^a^	**MBC** ^a^	**Biofilm Reduction**	**MIC** ^a^	**MBC** ^a^	**Biofilm Reduction**
**2**	0.06875	0.06875	0%	n.d. ^b^	n.d. ^b^	0%	n.d. ^b^	n.d. ^b^	0%
**3**	0.06875	0.06875	0%	n.d. ^b^	n.d. ^b^	0%	n.d. ^b^	n.d. ^b^	0%
**4**	0.03438	0.06875	0%	n.d. ^b^	n.d. ^b^	0%	n.d. ^b^	n.d. ^b^	0%
**5**	0.06875	0.06875	0%	n.d. ^b^	n.d. ^b^	0%	n.d. ^b^	n.d. ^b^	0%
**6**	0.06875	0.06875	0%	n.d. ^b^	n.d. ^b^	0%	n.d. ^b^	n.d. ^b^	0%
**7**	0.06875	0.06875	0%	n.d. ^b^	n.d. ^b^	0%	n.d. ^b^	n.d. ^b^	0%
**8**	0.06875	0.06875	0%	n.d. ^b^	n.d. ^b^	0%	n.d. ^b^	n.d. ^b^	0%
**9**	0.06875	0.06875	0%	n.d. ^b^	n.d. ^b^	0%	n.d. ^b^	n.d. ^b^	0%
**10**	0.06875	0.06875	0%	n.d. ^b^	n.d. ^b^	0%	n.d. ^b^	n.d. ^b^	0%
**11**	0.06875	0.06875	0%	n.d. ^b^	n.d. ^b^	0%	n.d. ^b^	n.d. ^b^	0%
**12**	0.03438	0.03438	0%	n.d. ^b^	n.d. ^b^	0%	n.d. ^b^	n.d. ^b^	0%
**octenidine**	-	0.000244	0.003906	-	0.000488	0.015625	-	0.003906	0.03125

^a^ MIC and MBC values are given in mg/mL; ^b^ n.d.—impact not detected within the range of tested concentrations.

**Table 2:** Results of molecular docking of the **2**–**12** compounds to the *S. aureus* DNA gyrase (PDB ID: 9N26; AutoDockVina 1.2.7 program, Vinardo scoring function). In most cases, the best pose is not the same as the experimental position of the reference ligand present in the PDB 9N26 deposit—the energy of the pose in contact with DNA is given in the last column. The re-docked reference ligand has a predicted affinity of -6.588 kcal/mol.

Compound	Best Pose Affinity [kcal/mol]	Does the Best Pose Overlap the Reference Ligand?	Is the Best Pose in Contact with DNA?	DNA–Overlapping Pose Affinity [kcal/mol]
**2**	−5.787	no	yes	−5.271
**3**	−5.907	no	no	−5.223
**4**	−6.465	no	yes	−5.474
**5**	−5.450	yes	yes	−5.450
**6**	−6.846	no	yes	−5.605
**7**	−5.641	yes	yes	−5.641
**8**	−4.932	no	no	−4.803
**9**	−4.981	yes	yes	−4.981
**10**	−4.819	no	no	−4.714
**11**	−4.771	no	yes	−4.554
**12**	−5.160	no	yes	−4.880

**Table 3:** Results of molecular docking of the **2**–**12** compounds to *E. coli* topoisomerase III type IA (PDB ID: 2O54; AutoDockVina 1.2.7 program, Vinardo scoring function). In most cases, the best pose is not in contact with the DNA strand of the PDB 2O54 deposit—the energy of the pose in contact with DNA is given in the last column.

Compound	Best Pose Affinity [kcal/mol]	Is the Best Pose in Contact with DNA?	DNA–Overlapping Pose Affinity [kcal/mol]
**2**	−4.358	no	−3.933
**3**	−5.049	no	−4.381
**4**	−4.888	no	none
**5**	−4.518	no	none
**6**	−5.444	no	−4.265
**7**	−5.644	no	−4.976
**8**	−4.837	no	none
**9**	−3.685	yes	−3.268
**10**	−5.025	no	none
**11**	−4.405	no	−4.124
**12**	−4.463	no	−4.057

## Data Availability

The original contributions presented in this study are included in the article/Supplementary Materials. Further inquiries can be directed to the corresponding author.

## Supplementary Materials

The following supporting information can be downloaded at https://www.mdpi.com/article/10.3390/molecules31203700/s1.

## Abbreviations

The following abbreviations are used in this manuscript:

ADME	Absorption, Distribution, Metabolism, Excretion
AMR	Antimicrobial Resistance
ATCC	American Type Culture Collection
BCP	Bond Critical Point
DFT	Density Functional Theory
DMSO	Dimethyl Sulfoxide
DNA	Deoxyribonucleic Acid
ESI-MS	Electrospray Ionization Mass Spectrometry
FDA	Food and Drug Administration
FT-IR	Fourier-Transform Infrared Spectroscopy
HR-MS	High-Resolution Mass Spectrometry
IEF-PCM	Integral Equation Formalism–Polarizable Continuum Model
InhA	Enoyl-Acyl Carrier Protein Reductase
M-H	Mueller-Hinton
MBC	Minimum Bactericidal Concentration
MBEC	Minimum Biofilm Eradication Concentration
MDR-TB	Multidrug-Resistant Tuberculosis
MIC	Minimum Inhibitory Concentration
MMEF	Molecular Mechanism Force Field
MS	Mass Spectrometry
MRSA	Methicillin-Resistant *Staphylococcus aureus*
NMR	Nuclear Magnetic Resonance
PBS	Phosphate-Buffered Saline
PDB	Protein Data Bank
PES	Potential Energy Surface
QTAIM	Quantum Theory of Atoms in Molecules
RCP	Ring Critical Point
TLC	Thin-Layer Chromatography
TMS	Tetramethylsilane
TTC	Triphenyltetrazolium Chloride
