# Supplementary material for: Pyridine-3-Carboxamide–1,3,4-Thiadiazole Hybrids as Antimicrobial Agents: Synthesis, Biological Evaluation, In Vivo Toxicity, and Computational Studies
Source: Molecules. 2026 Oct 15;31(20):3700. doi: 10.3390/molecules31203700 (PMC13707785; doi:10.3390/molecules31203700)
Supplement: Supplementary file 1 [file molecules-31-03700-s001.zip › molecules-4583898-supplementary.pdf]

Supplementary Materials

Pyridine-3-Carboxamide–1,3,4-Thiadiazole Hybrids as Antimicrobial Agents: Synthesis, Biological Evaluation, In Vivo Toxicity, and Computational Studies

Piotr Świątek [1,*], Małgorzata Strzelecka [1], Łukasz Szczukowski [1], Aneta Jezierska [2], Jarosław J. Panek [2], Adam Junka [3,4], Aleksandra Woytoń [3], Roman Lesyk [5,6,7] and Teresa Glomb [1]

1 Department of Medicinal Chemistry, Faculty of Pharmacy, Wroclaw Medical University, Borowska 211, 50-556 Wroclaw, Poland; malgorzata.strzelecka@umw.edu.pl (M.S.); lukasz.szczukowski@umw.edu.pl (Ł.S.); teresa.glomb@umw.edu.pl (T.G.)
2 Faculty of Chemistry, University of Wroclaw, F. Joliot-Curie 14, 50-383 Wroclaw, Poland; aneta.jezierska@uwr.edu.pl (A.J.); jaroslaw.panek@uwr.edu.pl (J.J.P.)
3 Platform for Unique Models Application, Department of Translational Technologies, Faculty of Pharmacy, Wroclaw Medical University, Borowska 211, 50-556 Wroclaw, Poland; adam.junka@umw.edu.pl (A.J.); aleksandra.woyton@student.umw.edu.pl (A.W.)
4 College of Life Sciences and Medicine, Zhejiang Sci-Tech University, Hangzhou 310018, China
5 Department of Pharmaceutical, Organic and Bioorganic Chemistry, Danylo Halytsky Lviv National Medical University, Pekarska 69, 79010 Lviv, Ukraine; dr_r_lesyk@org.lviv.net
6 Molecular Design Center, Danylo Halytsky Lviv National Medical University, Pekarska 69, 79010 Lviv, Ukraine
7 Department of Biotechnology and Cell Biology, Medical College, University of Information Technology and Management in Rzeszow, Sucharskiego 2, 35-225 Rzeszow, Poland
* Correspondence: piotr.swiatek@umw.edu.pl

Table of contents:

Table S1. Visualizations of Nuclear Magnetic Resonance (NMR) spectra of compounds **2-12** (DMSO-d_6)... 3
Table S2. Visualizations of Fourier-Transform Infrared (FT-IR) spectra of compounds **2-12**.. 14
Table S3. Visualizations of High Resolution Mass Spectrometry (HRMS) spectra of compounds **2-12**... 17
Table S4. Average growth inhibition zones of microorganisms after exposure to compounds investigated 22
Figure S1. Optimized structures of compounds **2 - 12** obtained as a result of DFT/ωB97XD/6-311+G(d,p) simulations in the gas phase and with solvent reaction field 23
Scheme S1. Core part of the studied compounds. Some hydrogen atoms are omitted for clarity. The atom numbering scheme was prepared especially for the study and QTAIM analyses… 34

Table S5. Selected metric parameters (see Scheme S1) obtained at the DFT/ωB97XD/6-311+G(d,p) level of theory in the gas phase and using IEF-PCM solvation model (water as a solvent) of three investigated compounds (denoted as **3**, **6**, and **7**)………………………… 35

Table S6. Partial atomic charges for selected atoms (see Scheme S1) according to the QTAIM theory (the data are given for the gas phase as well as IEF-PCM results)..…………………. 41

Table S7. Electron density at selected Bond and Ring Critical Points (B/RCPs) obtained for compounds denoted as **3**, **6**, and **7** derived from the QTAIM theory. The data are given for the gas phase as well as the solvent reaction field (IEF PCM; water as a solvent). For details, see Scheme S1………………………………………………………………………………… 42

Figure S2. QTAIM molecular graphs of compounds **2** – **12** obtained as a result of the DFT/ωB97XD/6-311+G(d,p) simulations in the gas phase (two presentations to show the detected network of noncovalent interactions). Color coding: carbon – grey, oxygen – red, nitrogen – blue, sulphur – yellow, hydrogen – white, fluorine – green (compound **4**). Dotted lines indicate the network of detected noncovalent interactions. Green and red spheres indicate the presence of Bond and Ring Critical Points (BCPs and RCPs), respectively…………………………………………………………………………..… 43

Figure S3. QTAIM molecular graphs of compounds **2** – **12** obtained as a result of the DFT/ωB97XD/6-311+G(d,p) simulations with solvent reaction field IEF-PCM and water as a solvent (two presentations to show the detected network of noncovalent interactions). Color coding: carbon – grey, oxygen – red, nitrogen – blue, sulphur – yellow, hydrogen – white, fluorine – green (compound **4**). Dotted lines indicate the network of noncovalent interactions. Green and red spheres indicate the presence of Bond and Ring Critical Points (BCPs and RCPs), respectively…………………………………………………………………………... 54

Table S8. Physicochemical properties obtained for the set of the investigated compounds (**2** – **12**) according to SwissADME predictor……………………………………….………….. 65

Table S9. Lipophilicity and water solubility according to SwissADME predictor…………. 66

Table S10. Pharmacokinetics, druglikeness and medicinal chemistry………….…………. 67

Figure S4. Bioavailability radar obtained as a result of SwissADME analysis…………..… 68

Figure S5. Redocking of the original ligand N-{(2r,5r)-2-[2-(6-methoxyquinolin-4-yl)ethyl]-1,3-dioxan-5-yl}-3-oxo-3,4-dihydro-2H-1,4-benzothiazine-6-carboxamide into the DNA gyrase (PDB deposit 9N26). …………………………………………………………...…… 71

Coordinates of compound 2 - 12 for simulations in the gas phase and with solvent reaction field (IEF-PCM) and water as a solvent……………………………………….…………….. 72

Table S1. Visualizations of Nuclear Magnetic Resonance (NMR) spectra of compounds **2-12** (DMSO-*d*$_6$).

Comp.	^{1}H NMR, ^{13}C NMR spectra of **2-12**
**2**	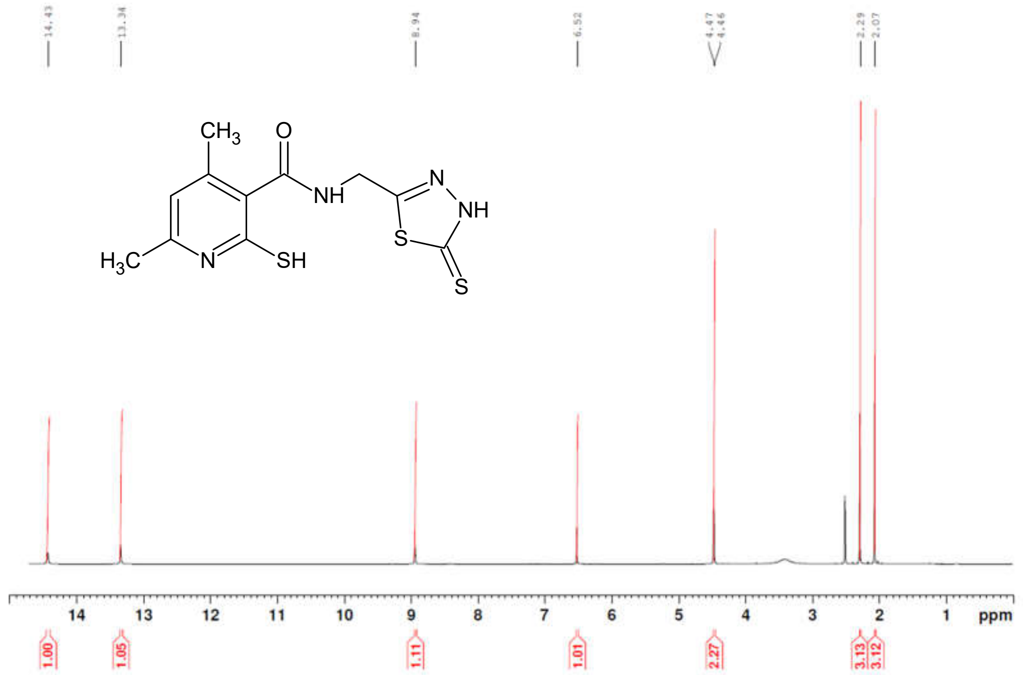 ^{1}H NMR of 2 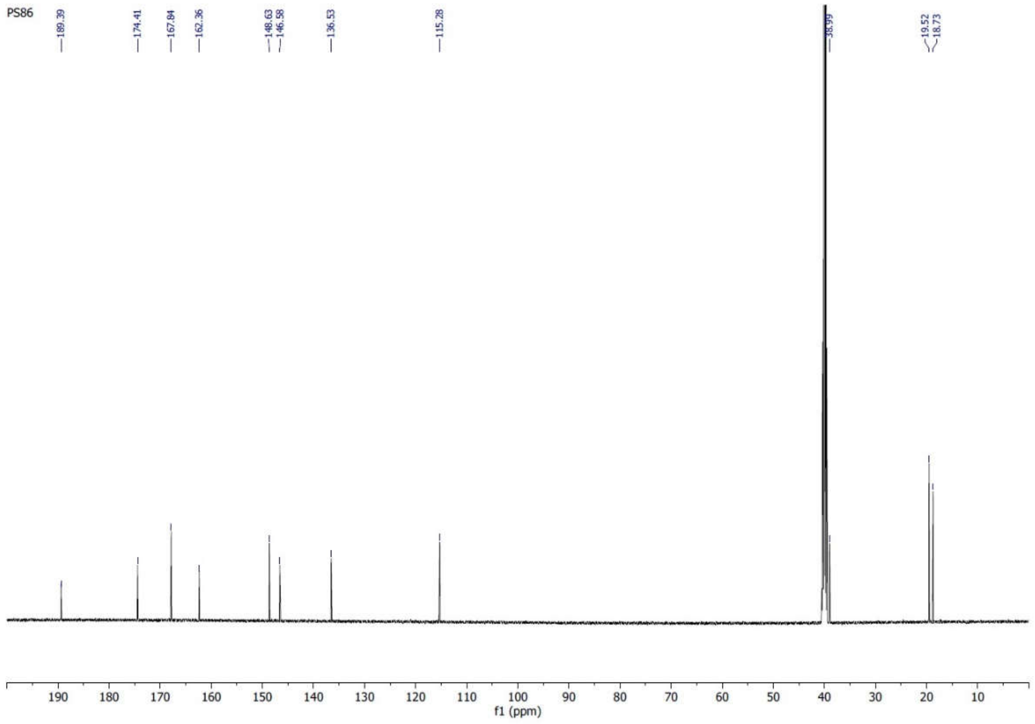 ^{13}C NMR of 2

3

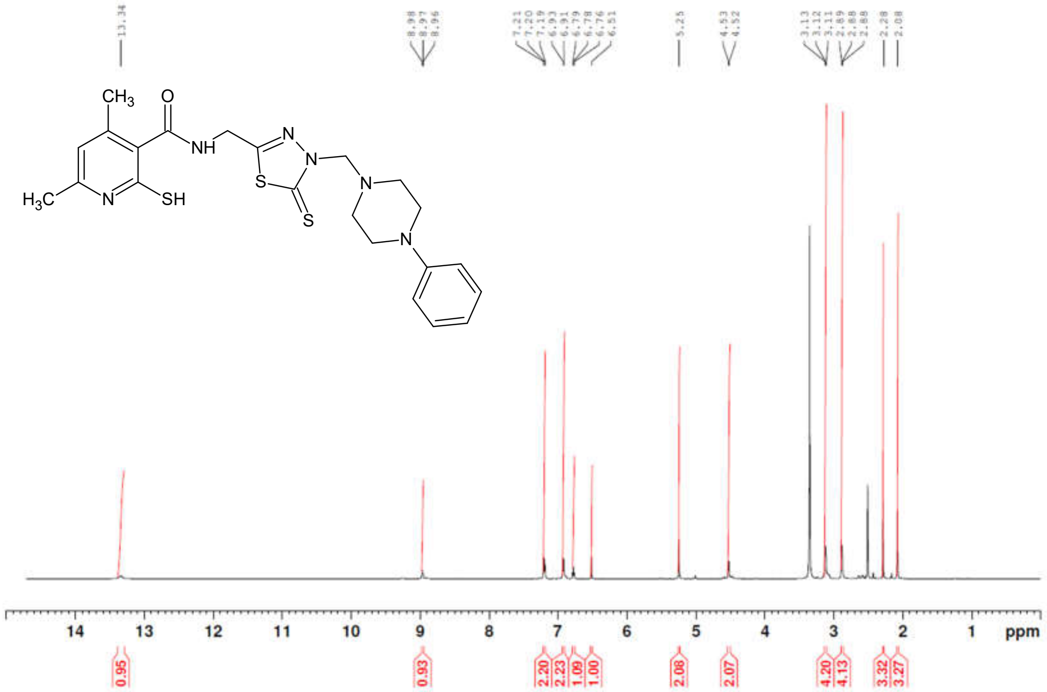

^{1}H NMR of 3

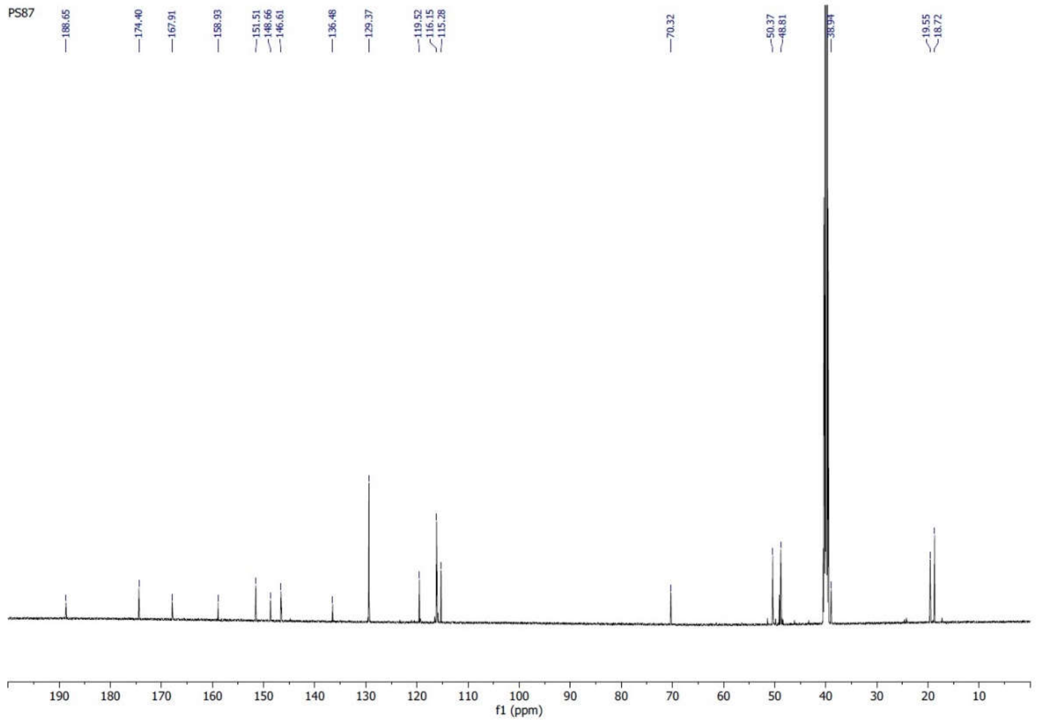

^{13}C NMR of 3

4

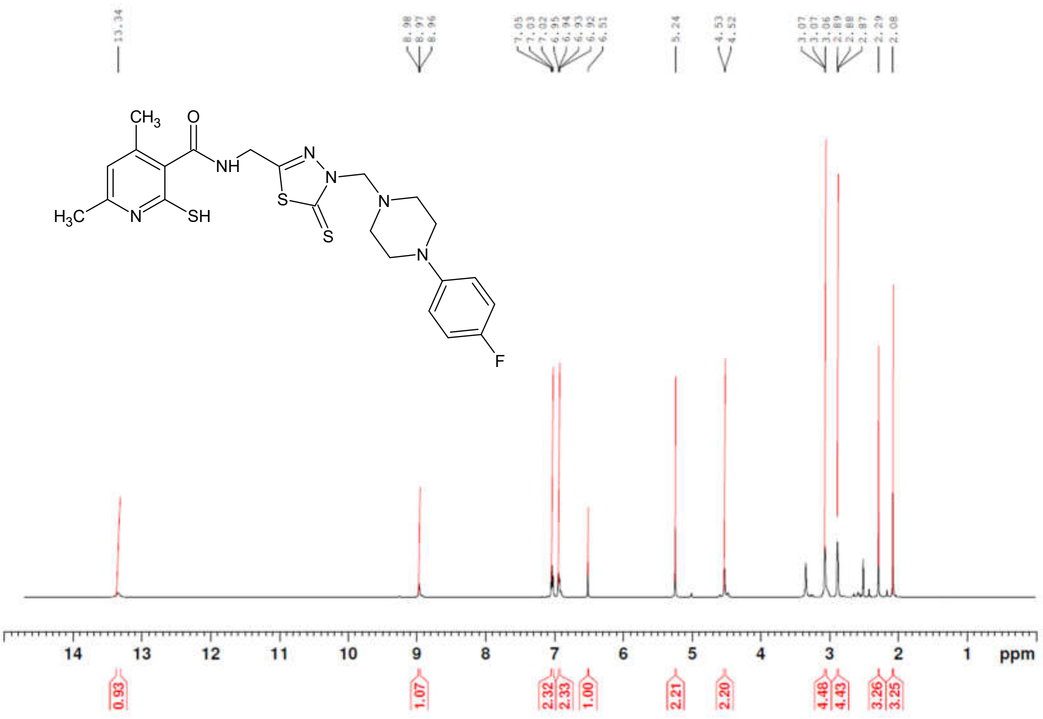

^{1}H NMR of 4

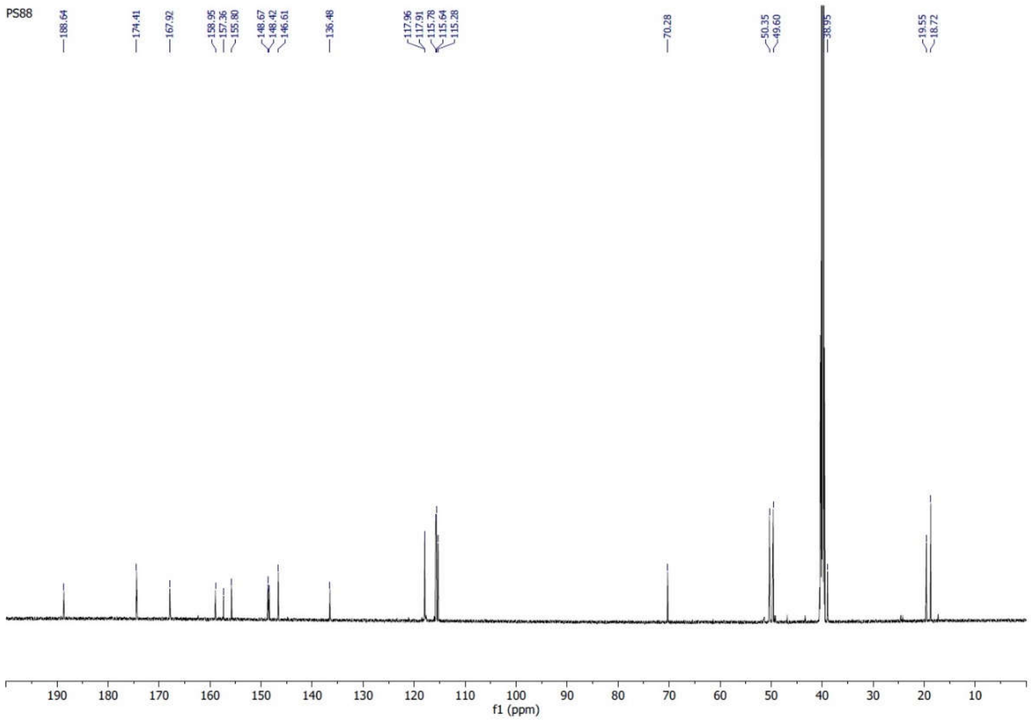

^{13}C NMR of 4

5

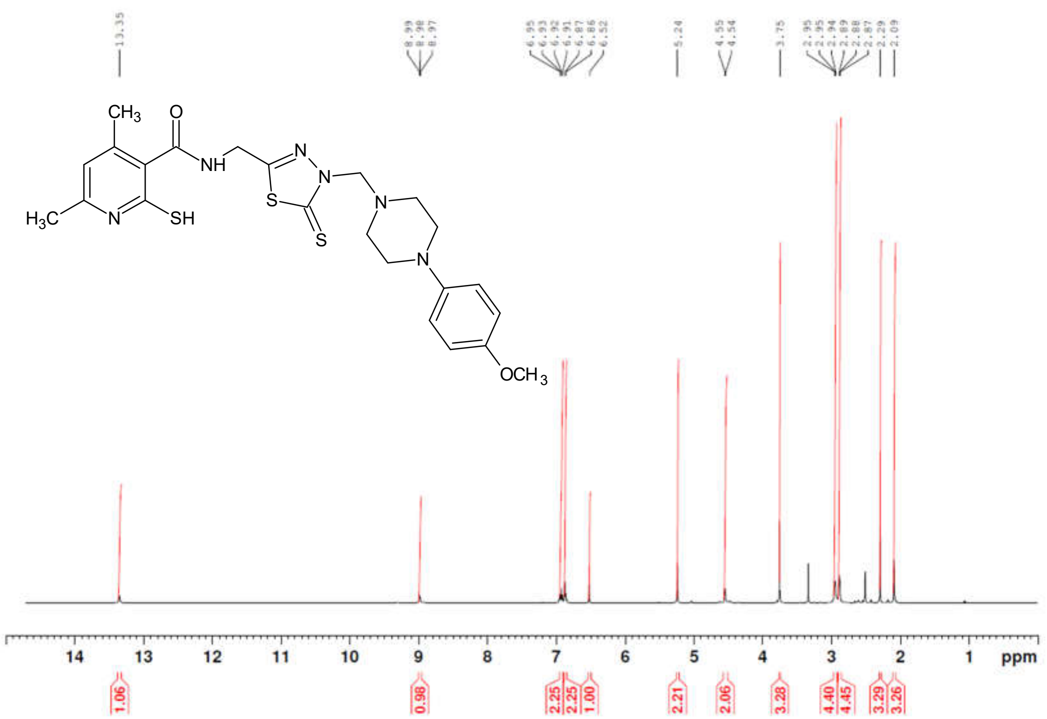

^{1}H NMR of 5

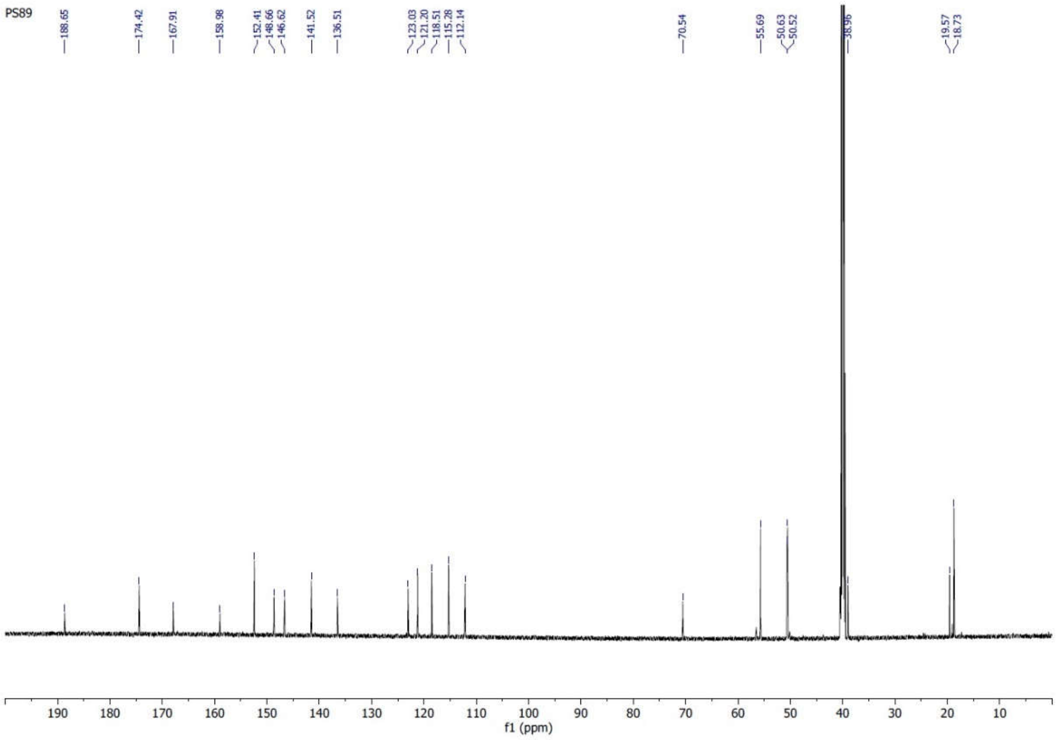

^{13}C NMR of 5

6

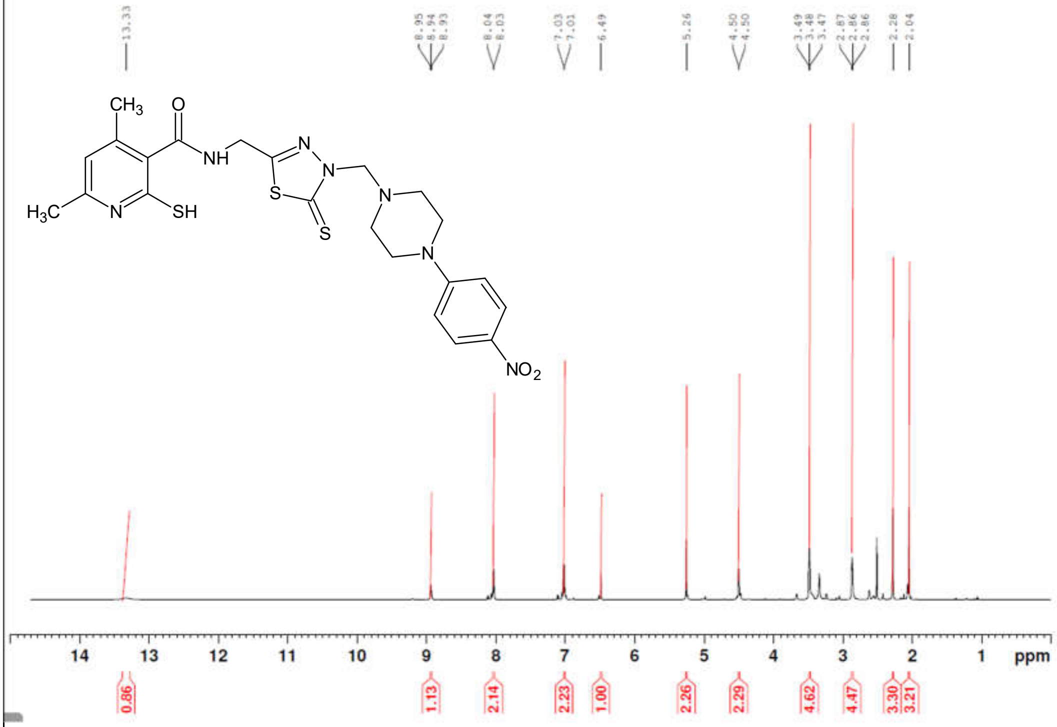

^{1}H NMR of 6

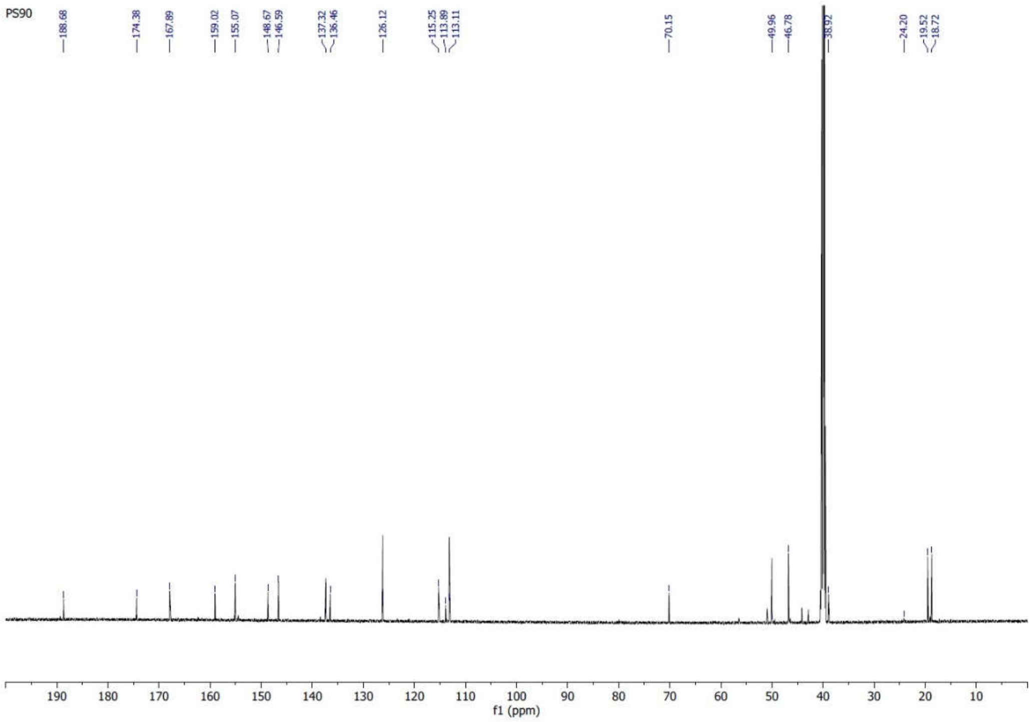

^{13}C NMR of 6

7

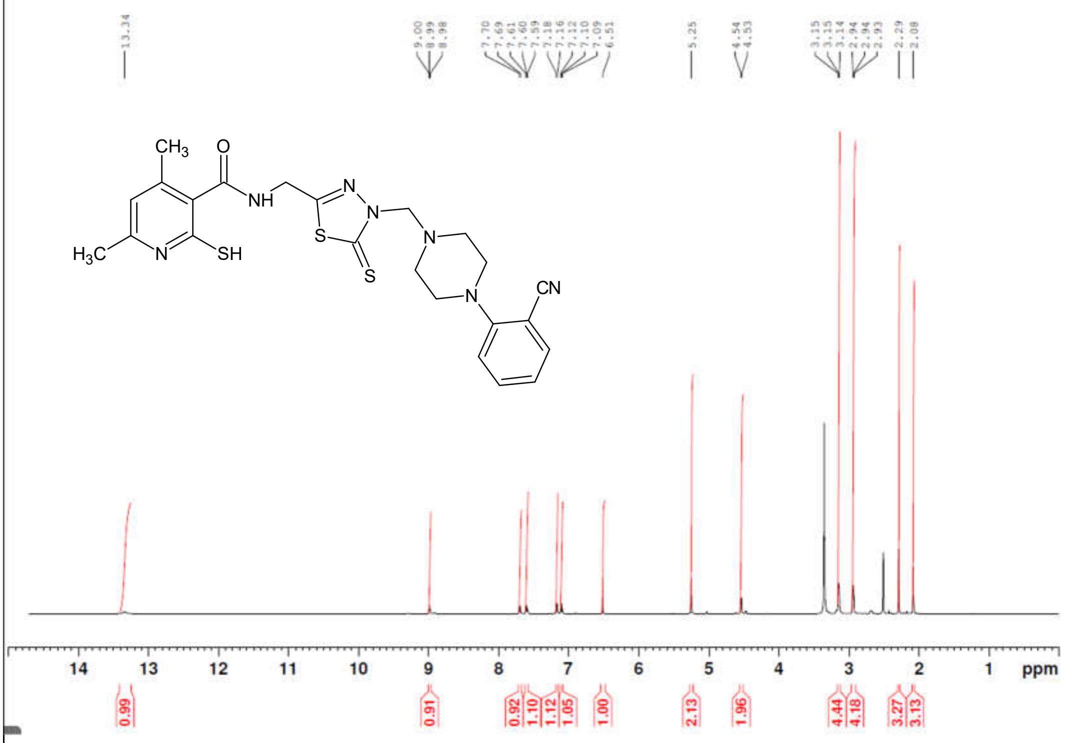

^{1}H NMR of 7

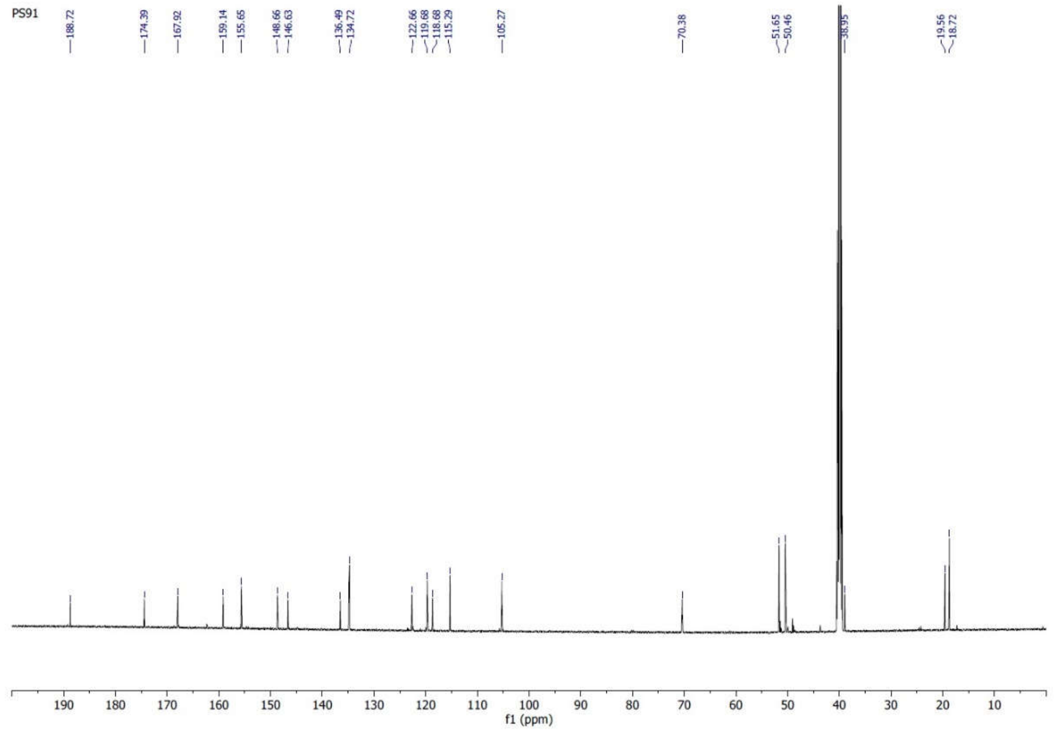

^{13}C NMR of 7

8

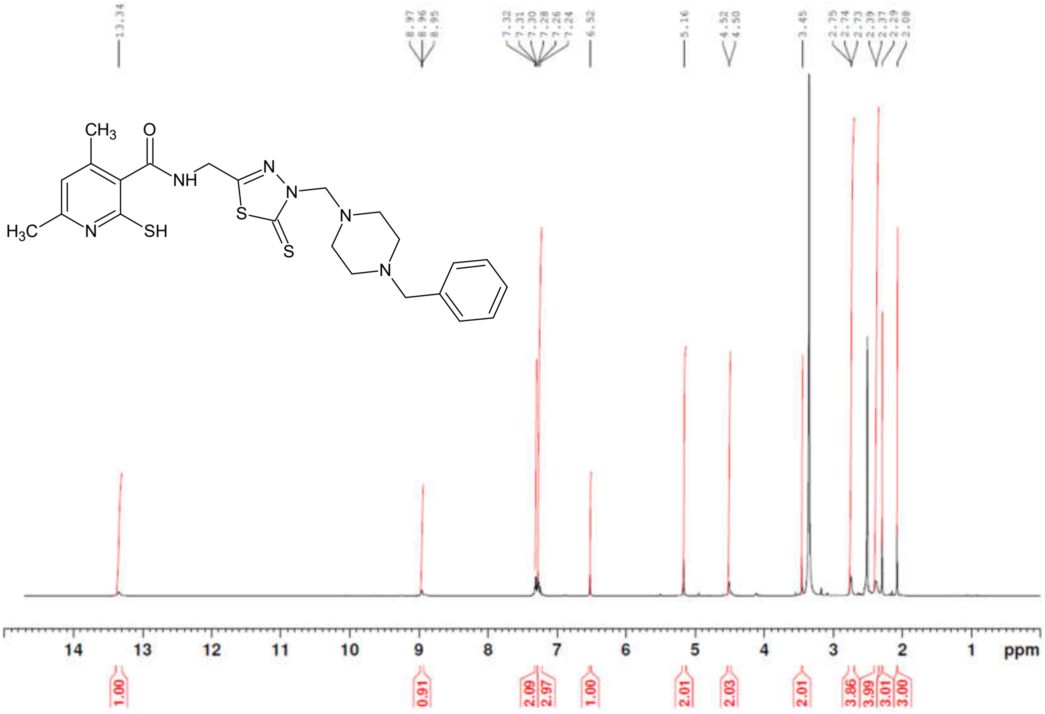

^{1}H NMR of 8

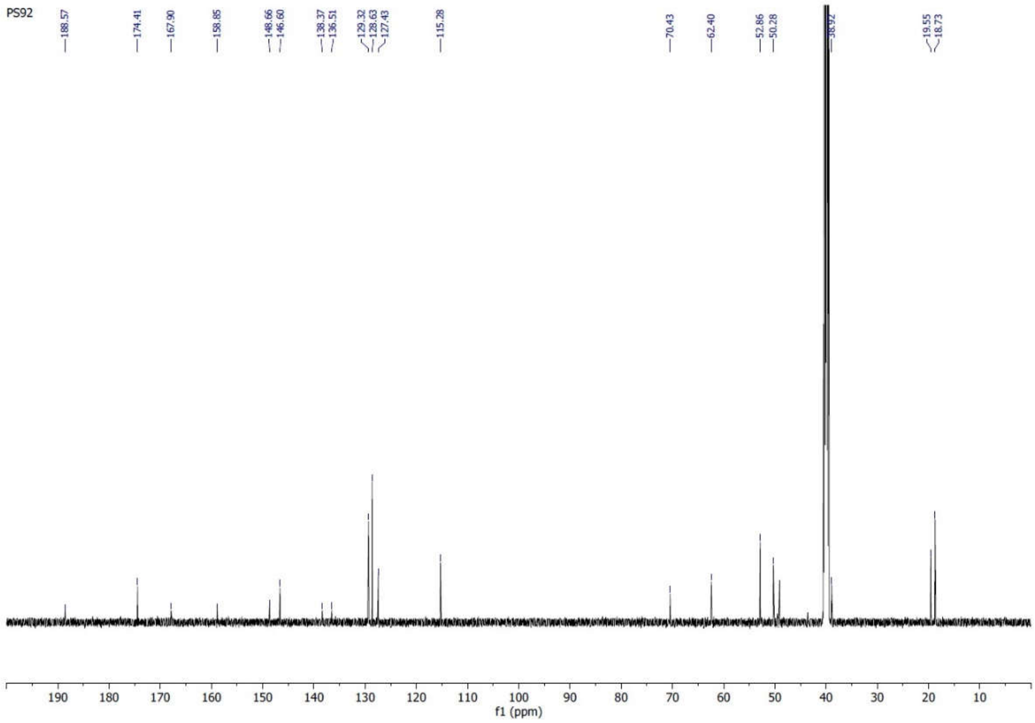

^{13}C NMR of 8

9

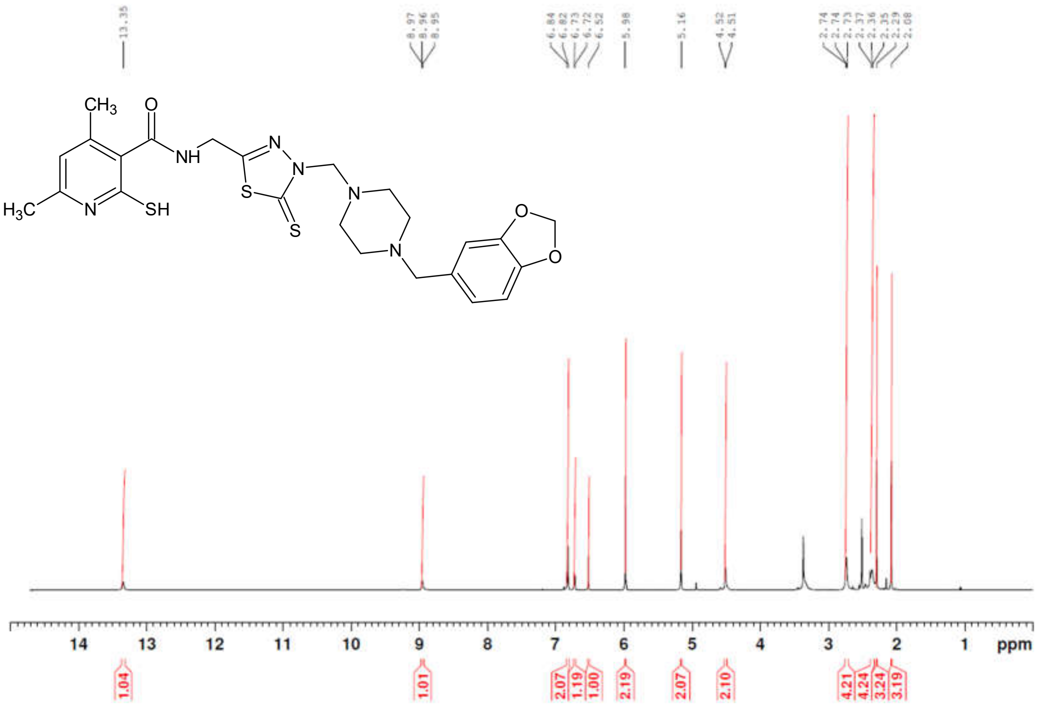

^{1}H NMR of 9

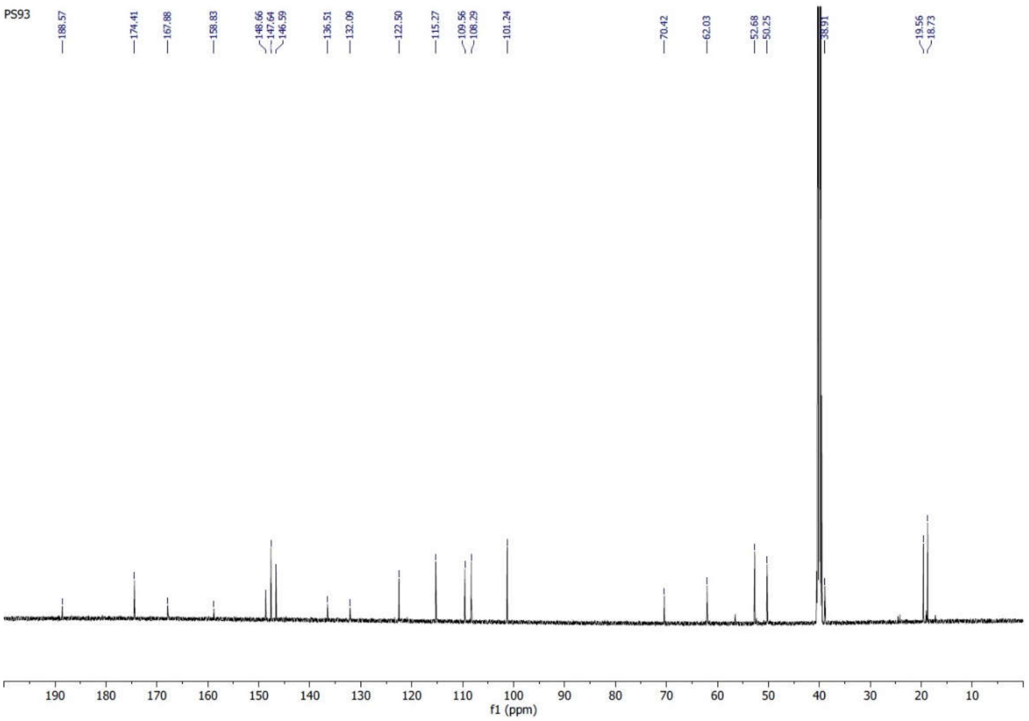

^{13}C NMR of 9

10

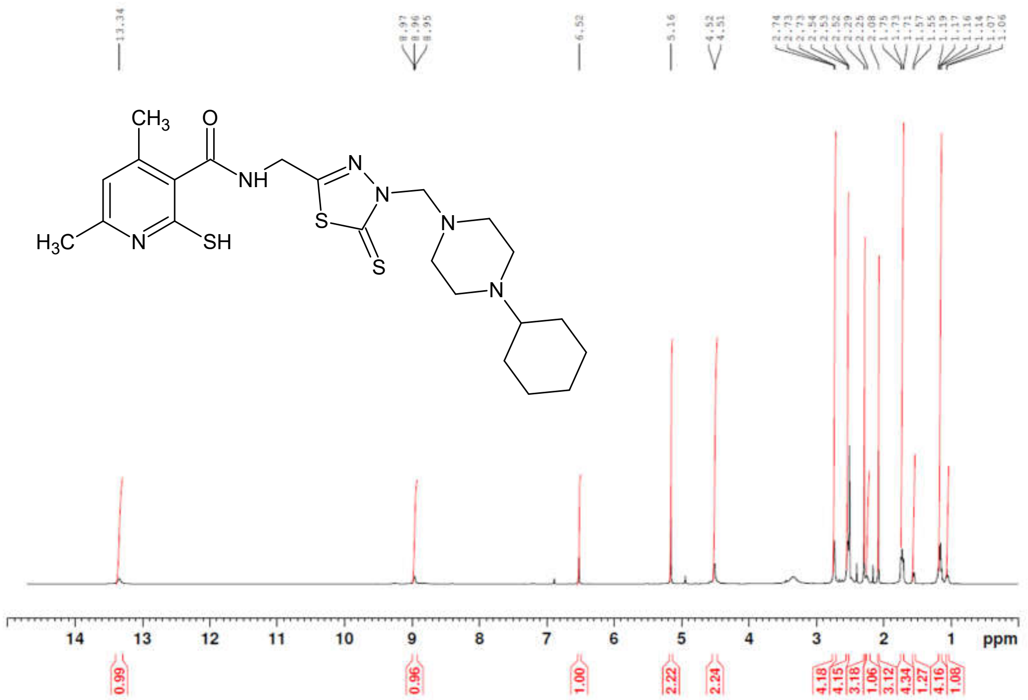

^{1}H NMR of 10

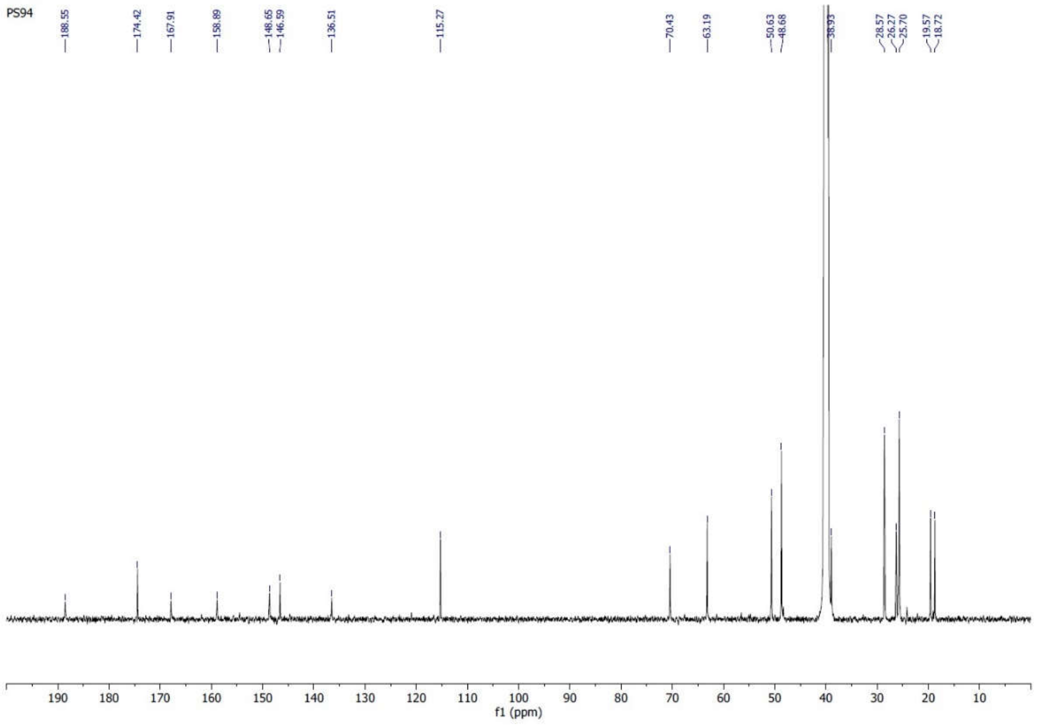

^{13}C NMR of 10

11

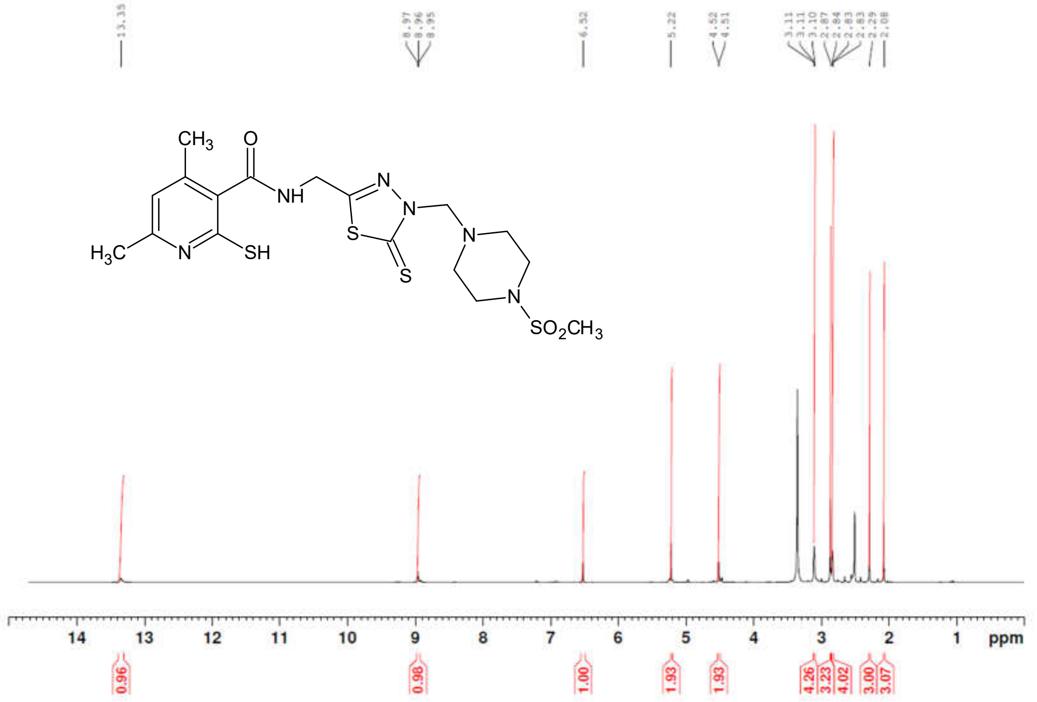

^{1}H NMR of 11

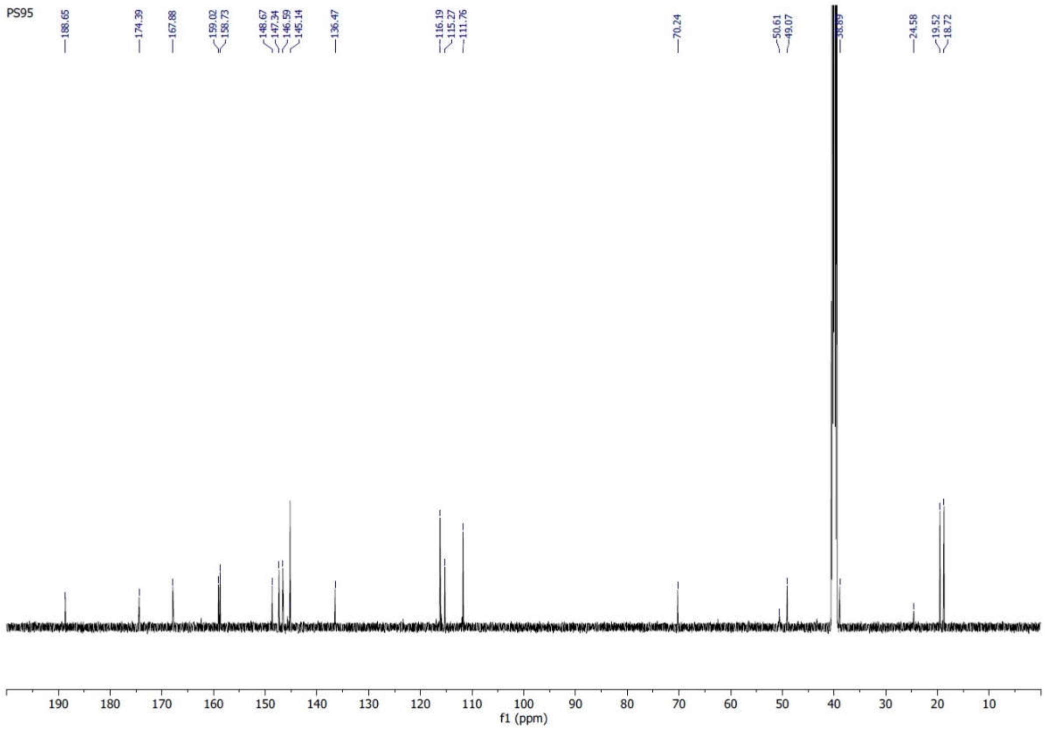

^{13}C NMR of 11

12

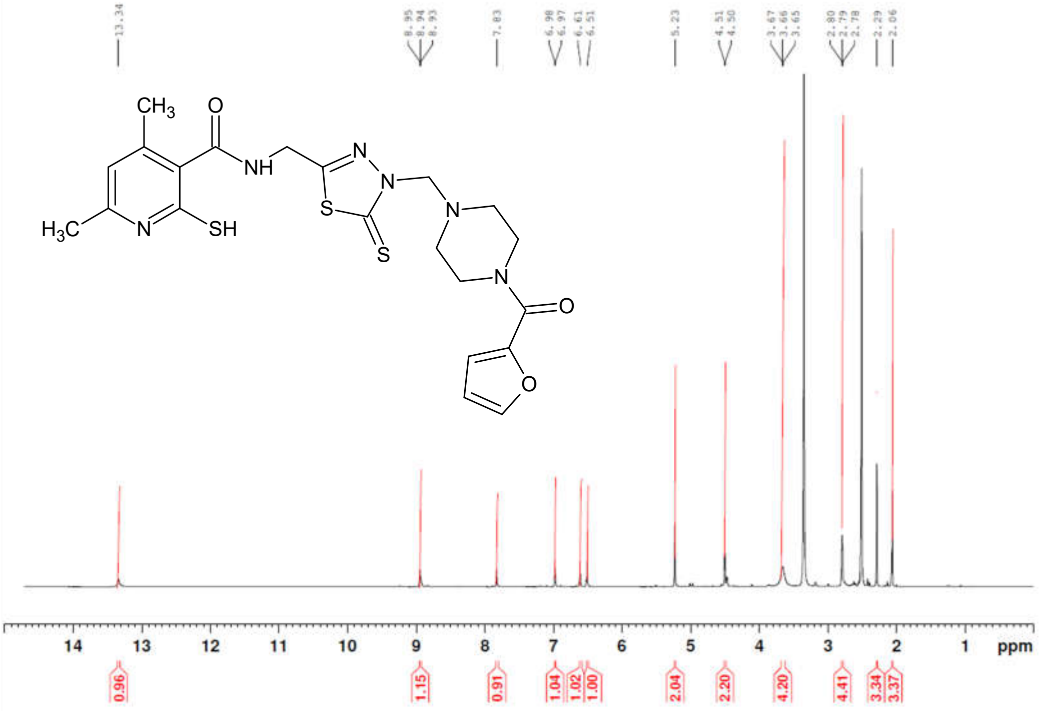

^{1}H NMR of 12

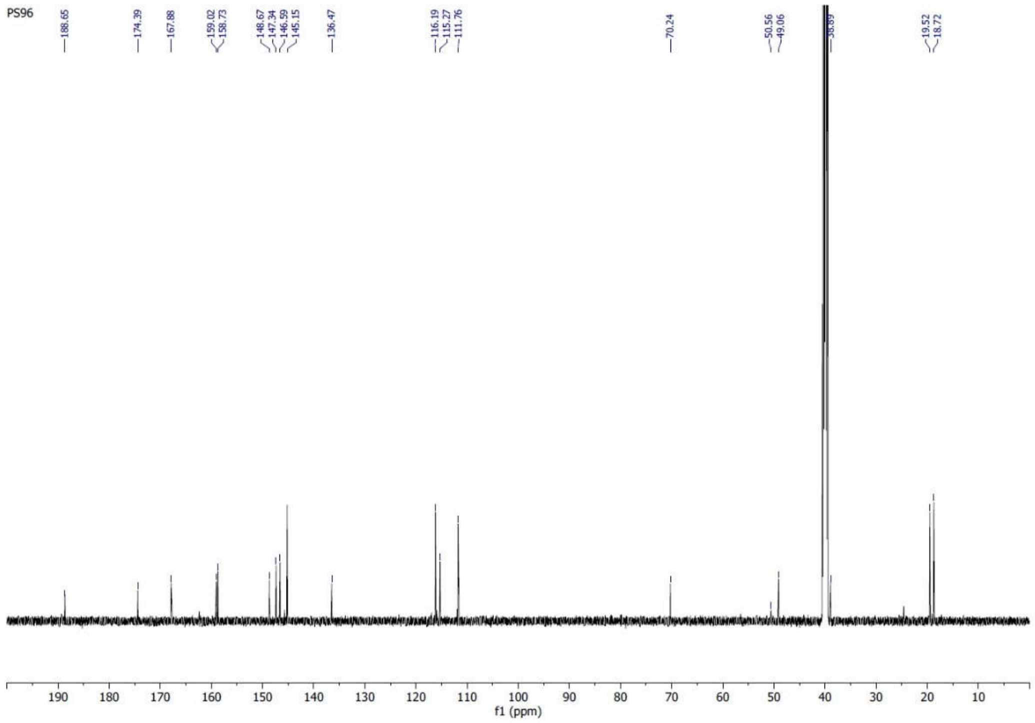

^{13}C NMR of 12

Table S2. Visualizations of Fourier-Transform Infrared (FT-IR) spectra of compounds **2-12**.

Comp**.**	ATR-FT-IR spectra
**2**	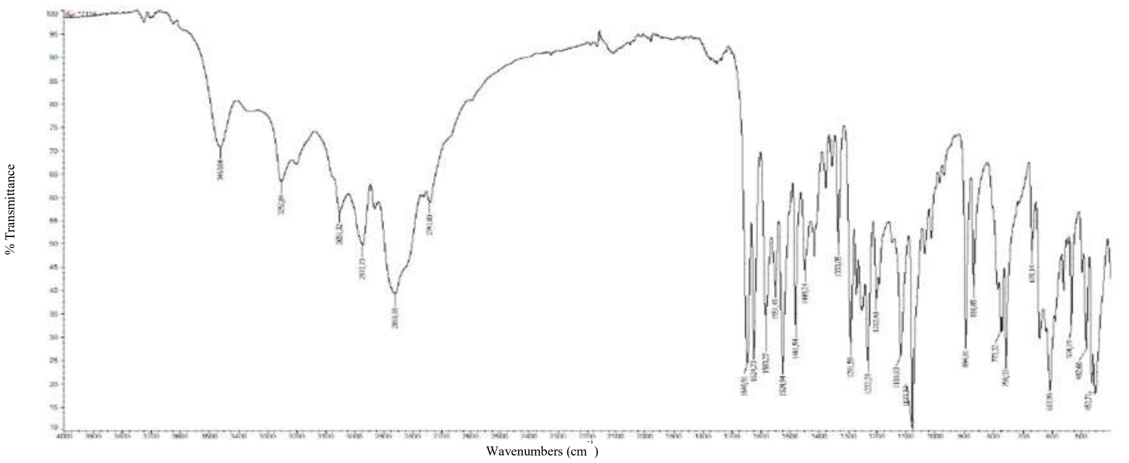
**3**	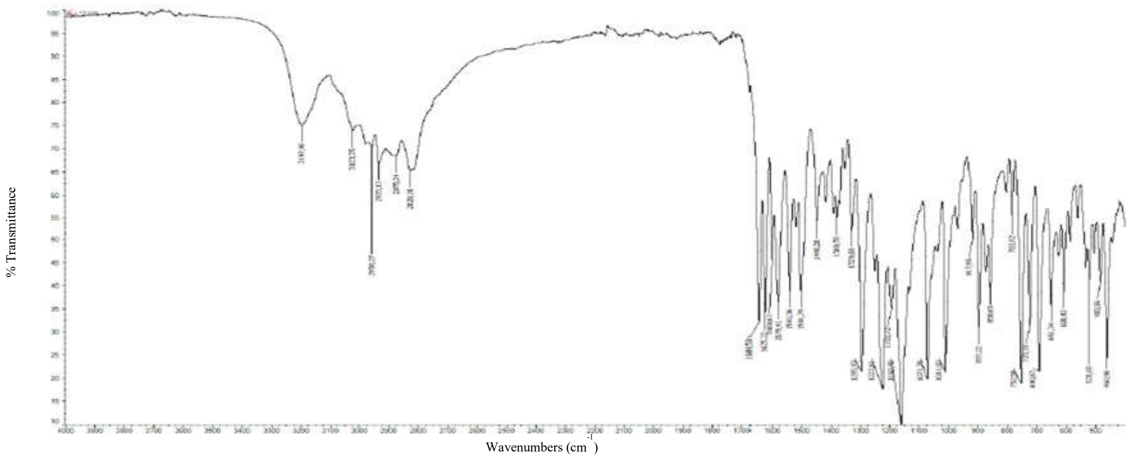
**4**	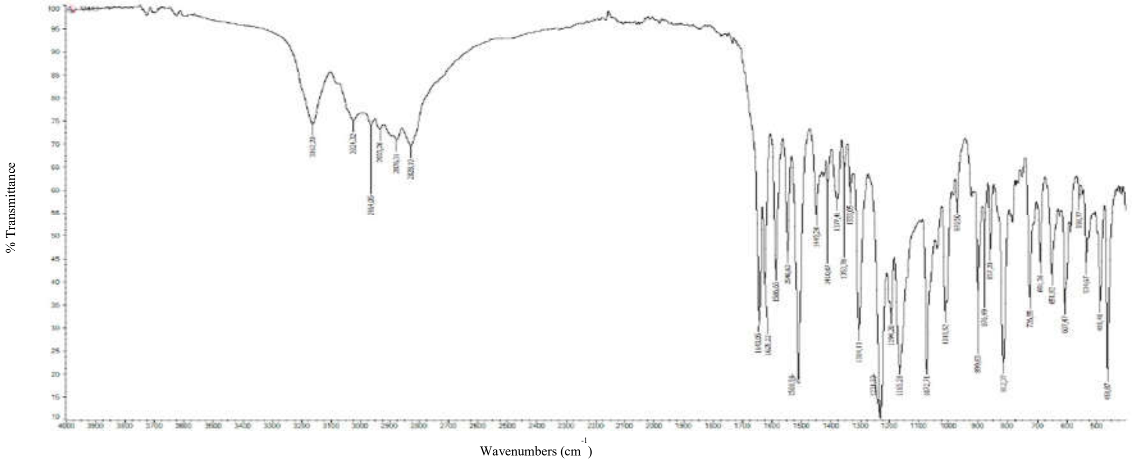

**5**	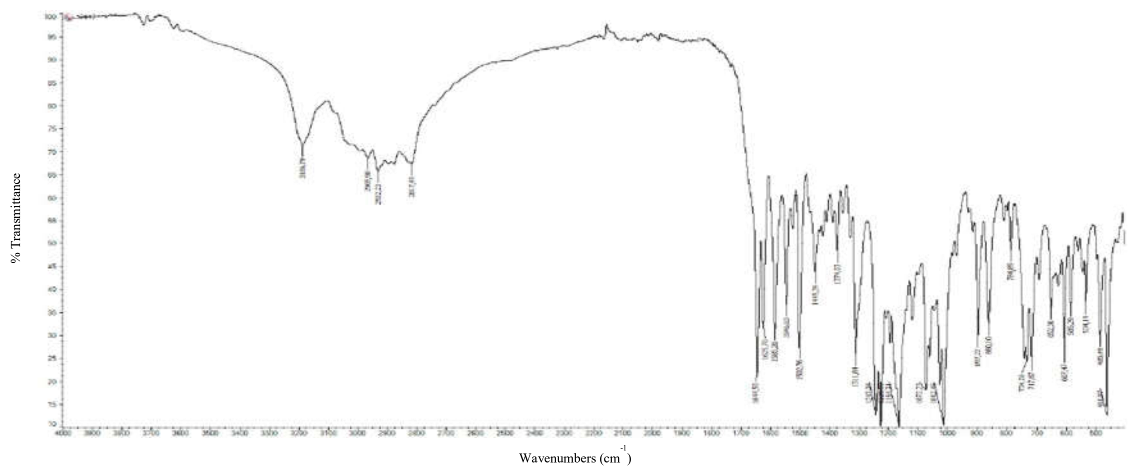 % Transmittance Wavenumbers (cm^{-1})
**6**	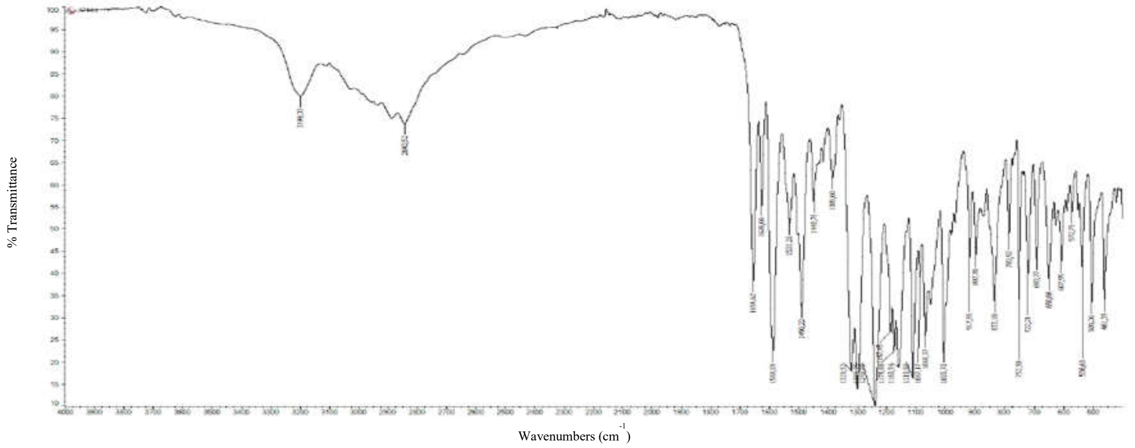 % Transmittance Wavenumbers (cm^{-1})
**7**	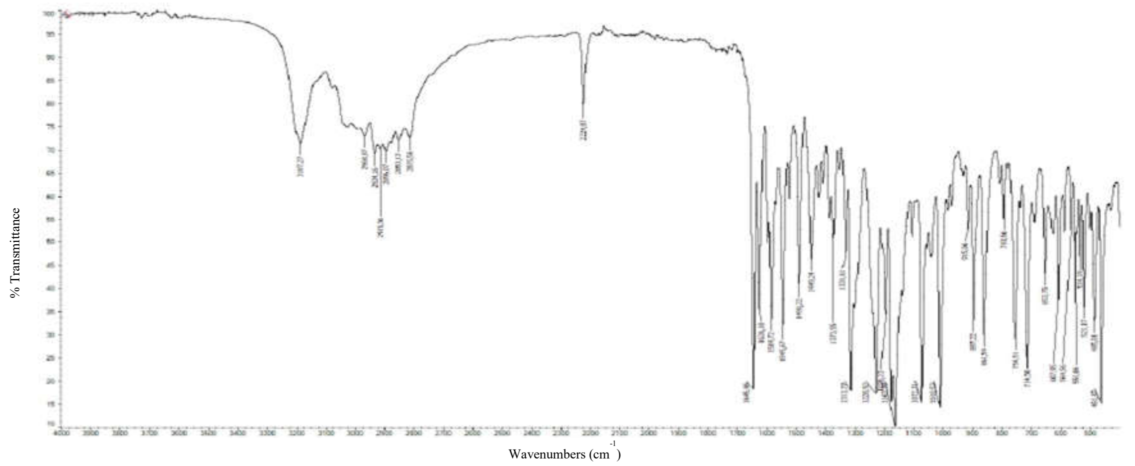 % Transmittance Wavenumbers (cm^{-1})

**8**	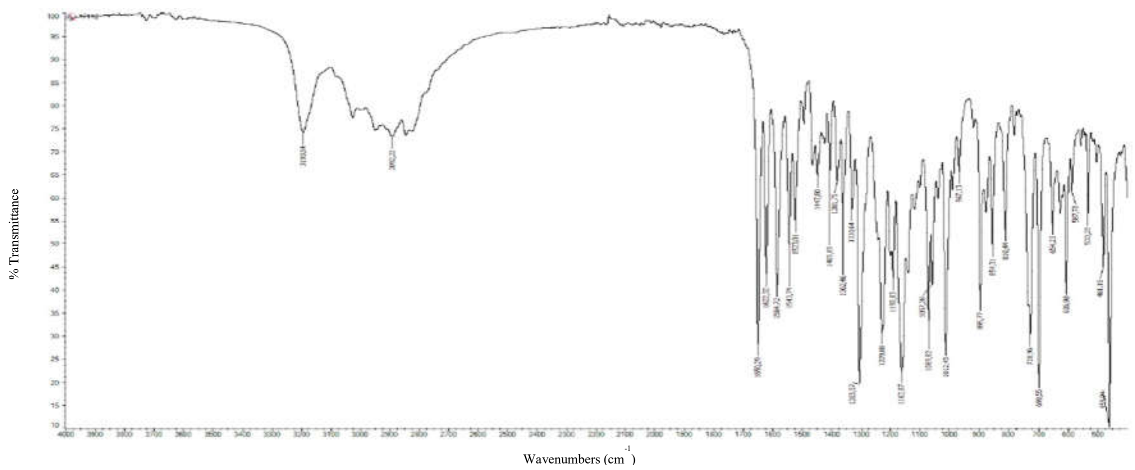% Transmittance<br>Wavenumbers (cm-1)
**9**	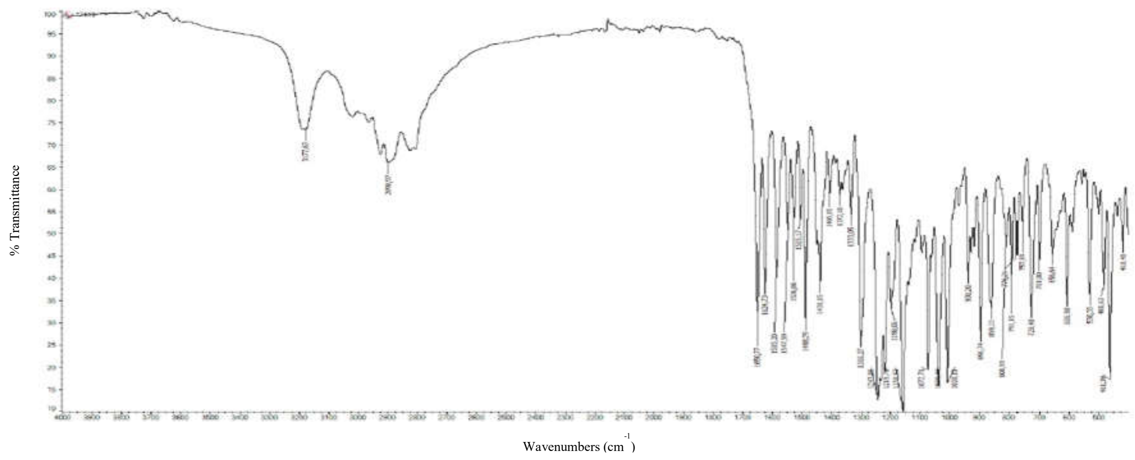% Transmittance<br>Wavenumbers (cm-1)
**10**	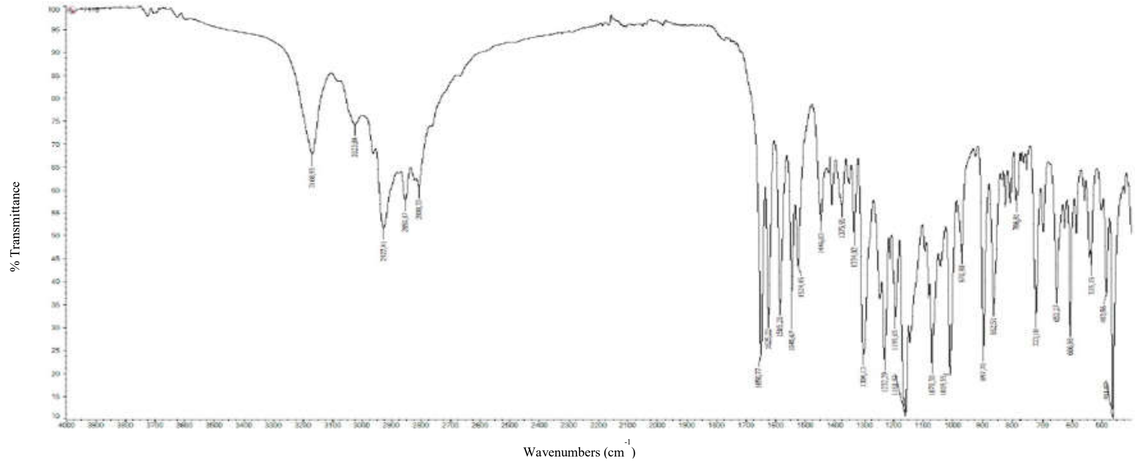% Transmittance<br>Wavenumbers (cm-1)

**11**	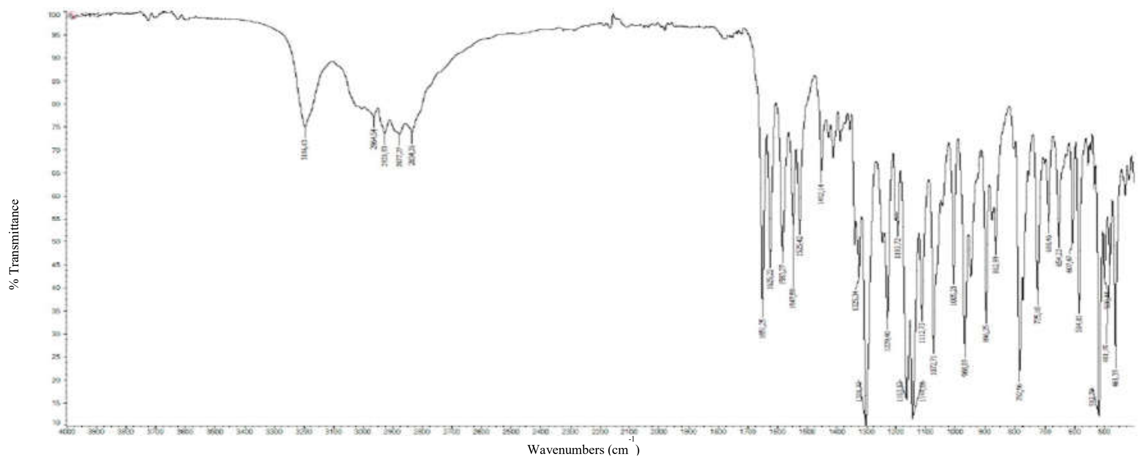
**12**	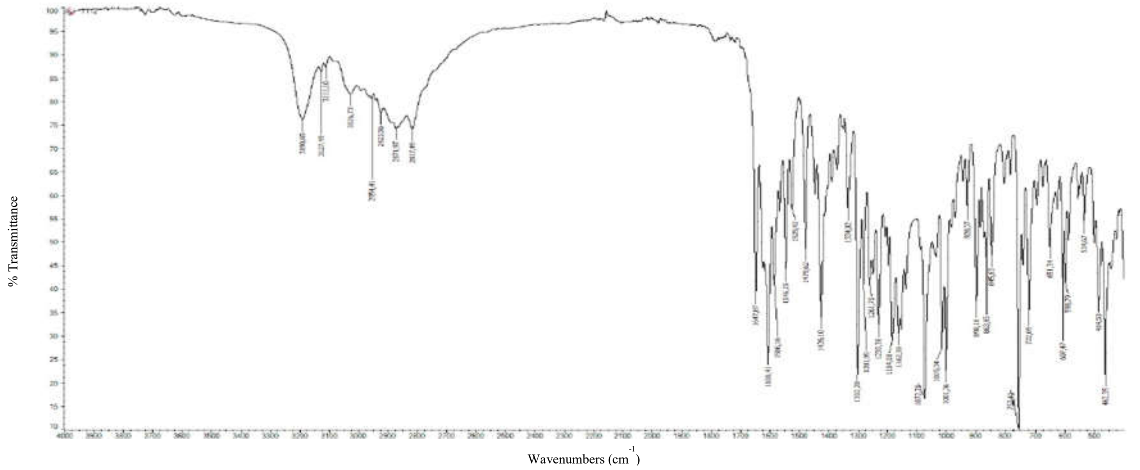

Table S3. Visualizations of High Resolution Mass Spectrometry (HRMS) spectra of compounds **2-12**

Comp.	HRMS spectra
**2**	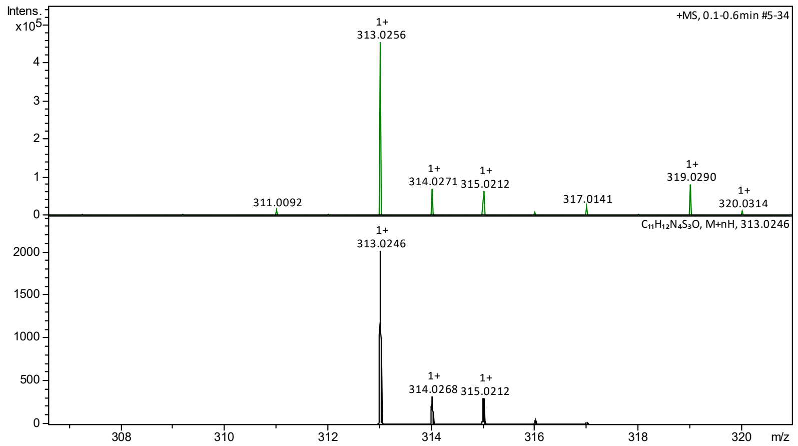

<table>
<tr><td>3</td><td>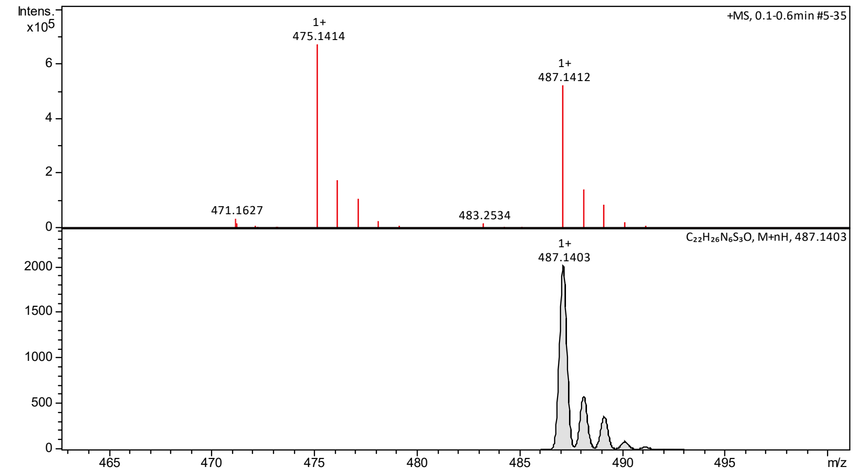
</td></tr>
<tr><td>4</td><td>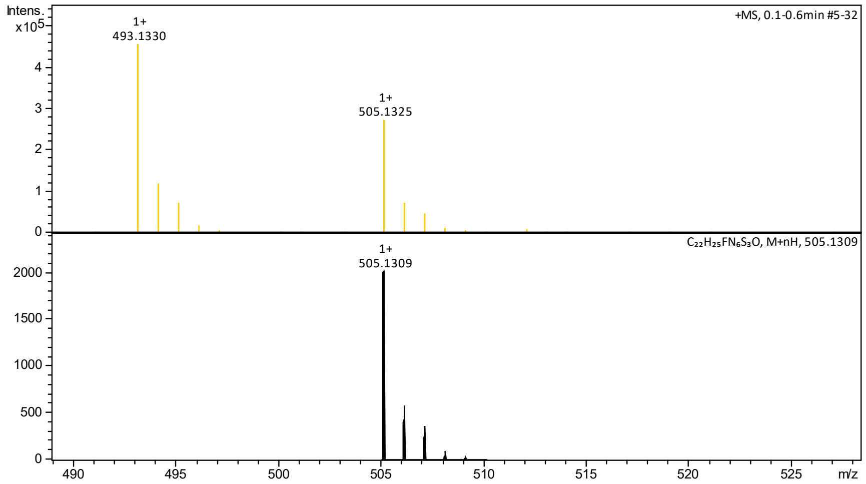
</td></tr>
<tr><td>5</td><td>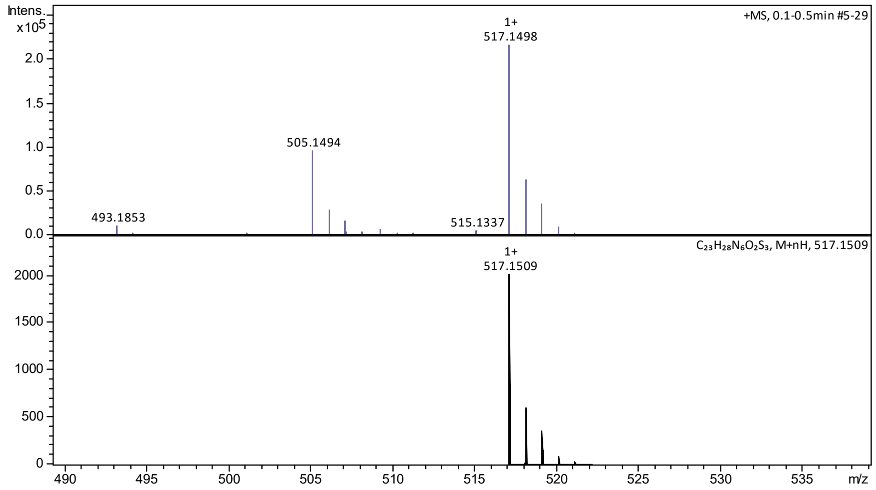
</td></tr>
</table>

<table>
<tr><td>6</td><td>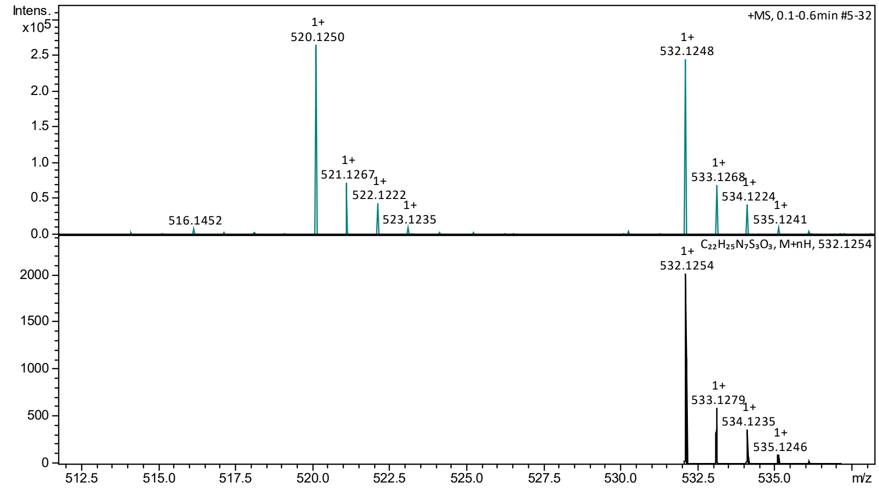
</td></tr>
<tr><td>7</td><td>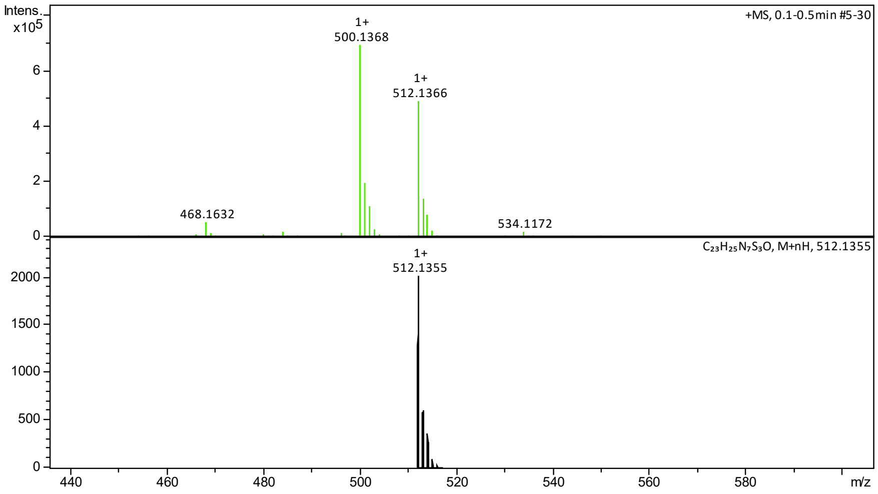
</td></tr>
<tr><td>8</td><td>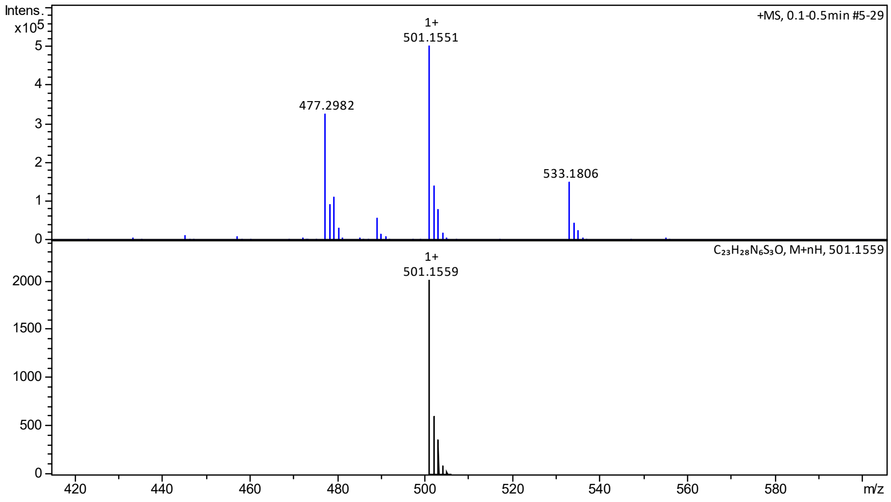
</td></tr>
</table>

**9**	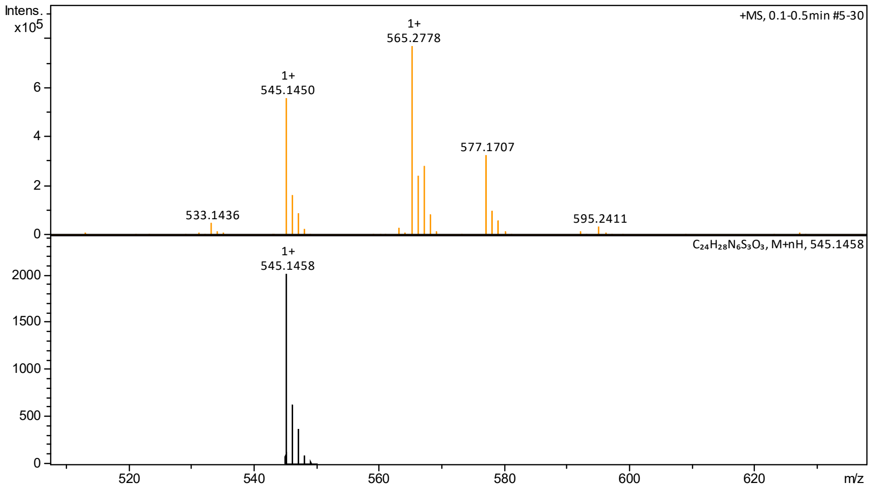
**10**	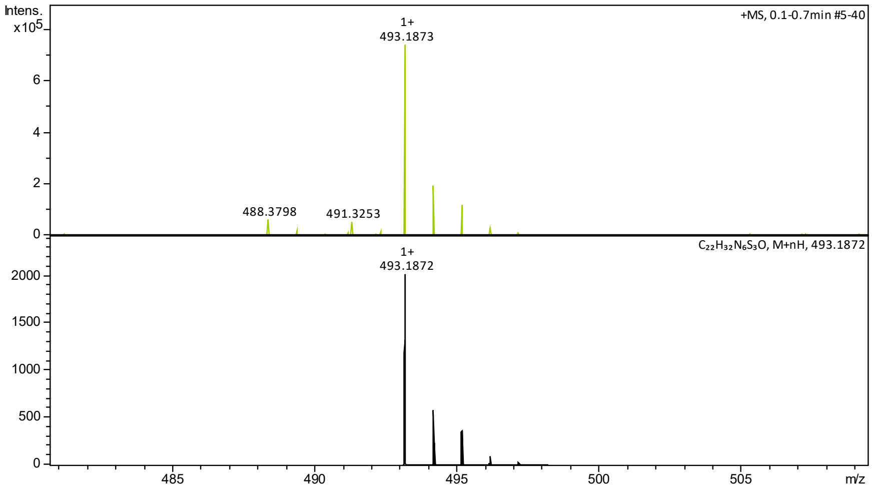
**11**	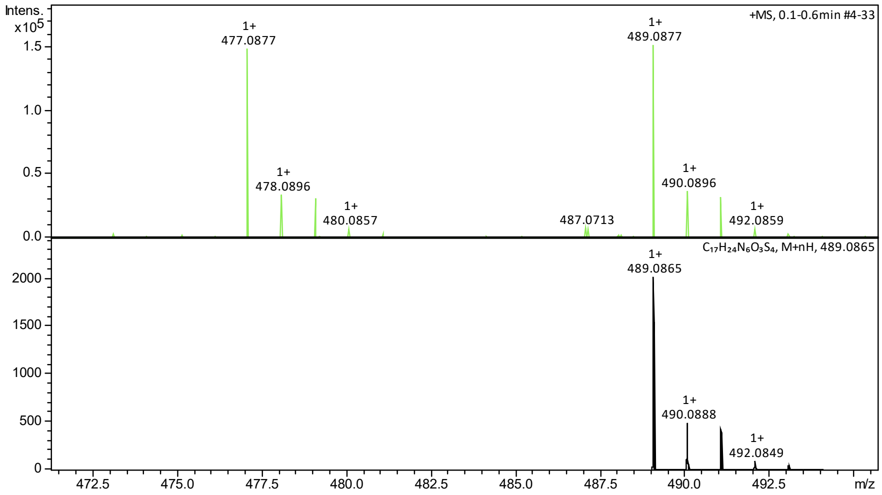

9
Intens. x10^5
+MS, 0.1-0.5min #5-30
1+ 565.2778
1+ 545.1450
577.1707
533.1436
595.2411
$C_{24}H_{28}N_6S_3O_3$, M+nH, 545.1458
1+ 545.1458
520 540 560 580 600 620 m/z

10
Intens. x10^5
+MS, 0.1-0.7min #5-40
1+ 493.1873
488.3798
491.3253
$C_{22}H_{32}N_6S_3O$, M+nH, 493.1872
1+ 493.1872
485 490 495 500 505 m/z

11
Intens. x10^5
+MS, 0.1-0.6min #4-33
1+ 477.0877
1+ 489.0877
1+ 478.0896
1+ 490.0896
1+ 480.0857
1+ 492.0859
487.0713
$C_{17}H_{24}N_6O_3S_4$, M+nH, 489.0865
1+ 489.0865
1+ 490.0888
1+ 492.0849
472.5 475.0 477.5 480.0 482.5 485.0 487.5 490.0 492.5 m/z

**12**	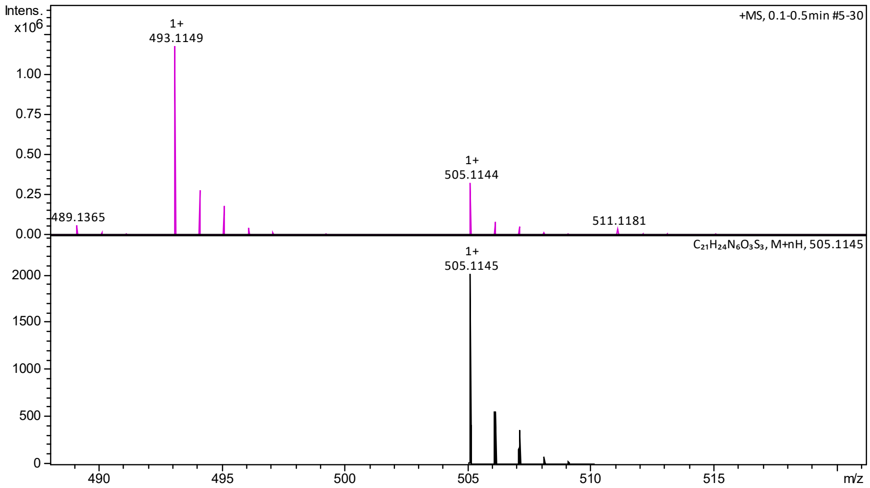

Intens.
x10^6
+MS, 0.1-0.5min #5-30
1+
493.1149
1+
505.1144
489.1365
511.1181
C21H24N6O3S3, M+nH, 505.1145
1+
505.1145
490
495
500
505
510
515
m/z

Table S4. Average growth inhibition zones of microorganisms after exposure to compounds investigated. Values are expressed in mm.

**Compound**	**Gram-positive pathogens**					**Gram-negative pathogens**			**Fungi**			**Commensal Gram-positive**	
	S. aureus ref.	***S. epidermidis*** ref.	***S. aureus*** clin.	***S. agalactiae*** ref.	***S. aureus*** clin.	***P. aeruginosa*** ref.	***R. ornithinolytica*** ref.	***K. variicola*** clin.	***C. albicans*** ref.	***C. albicans*** clin.	***A. niger*** ref.	***Corynebacterium*** sp.	***L. fermentum***
**2**	17.8	26.6	14.8	22.5	17.1	0	9.4	0	0	0	0	0	0
**3**	19.1	26.5	15.9	23	17.5	0	8.2	0	0	0	0	0	30.7
**4**	19.2	26.9	14.7	21.2	16	0	8	7	0	0	0	12	31.6
**5**	17.4	23	13.3	20.2	15.2	0	7	0	0	0	0	0	0
**6**	24	15.7	11.5	20.6	16	0	12.3	0	0	0	0	0	33.1
**7**	24.8	15.9	12.7	21.1	13.9	0	10.3	0	0	0	0	0	0
**8**	23.2	15.2	13.2	21	16.1	0	8.7	0	0	0	0	0	0
**9**	25.6	15.9	14.5	21.5	16.3	0	8.8	0	0	0	0	0	0
**10**	23.9	15.3	16.6	21	15.9	0	14.2	0	0	0	0	0	31.5
**11**	23.4	14.3	10	18.2	12.9	0	7.7	0	0	0	0	0	32.5
**12**	6.9	0	0	9	6.8	0	0	0	0	0	0	0	26.8

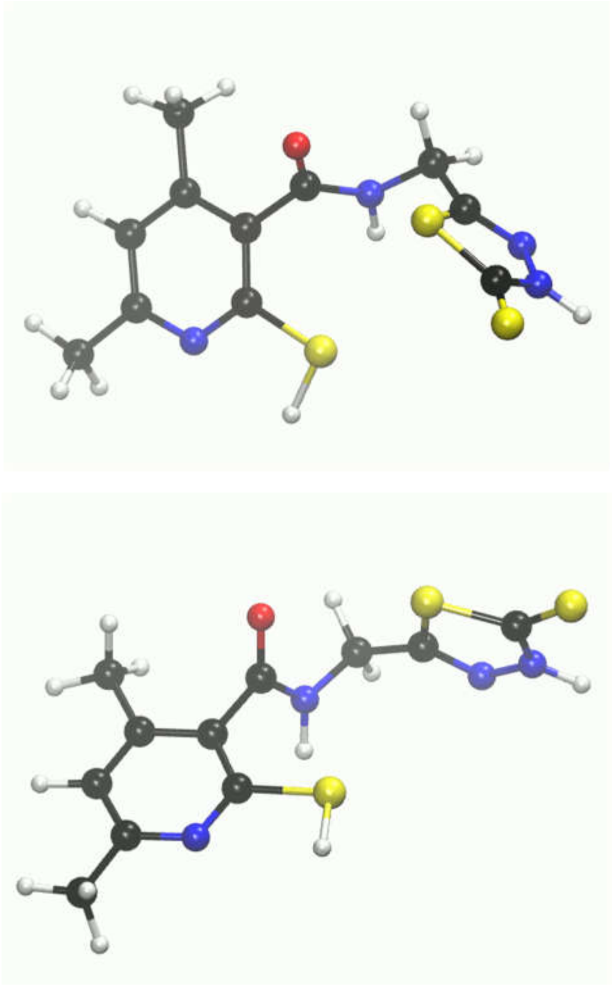

Compound 2

Figure S1. Optimized structures of compounds **2** – **12** obtained as a result of DFT/ωB97XD/6-311+G(d,p) simulations in the gas phase and with solvent reaction field. Color coding: carbon – black, oxygen – red, nitrogen – blue, sulphur – yellow, hydrogen – white.

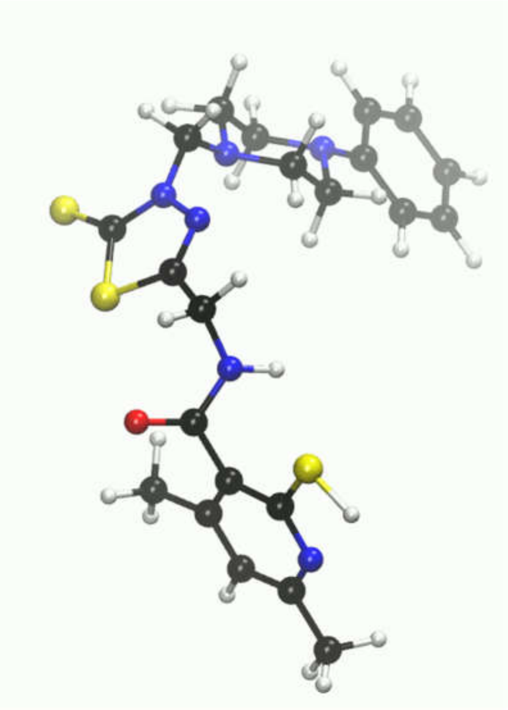

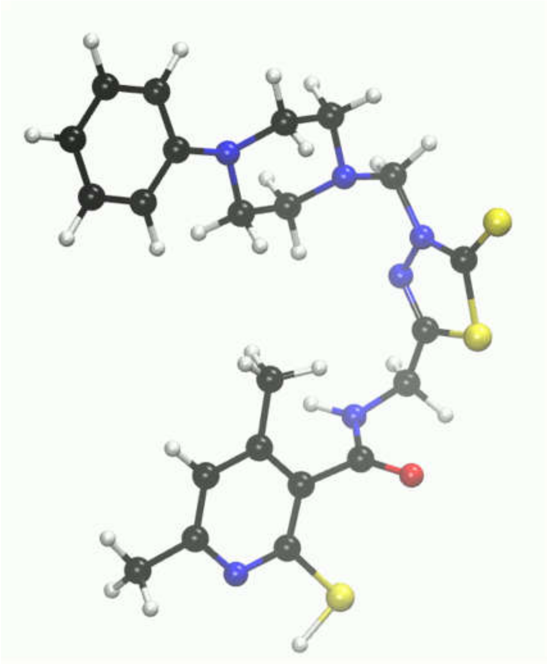

Compound 3

Figure S1 (Continuation). Optimized structures of compounds **2** – **12** obtained as a result of DFT/ωB97XD/6-311+G(d,p) simulations in the gas phase and with solvent reaction field. Color coding: carbon – black, oxygen – red, nitrogen – blue, sulphur – yellow, hydrogen – white.

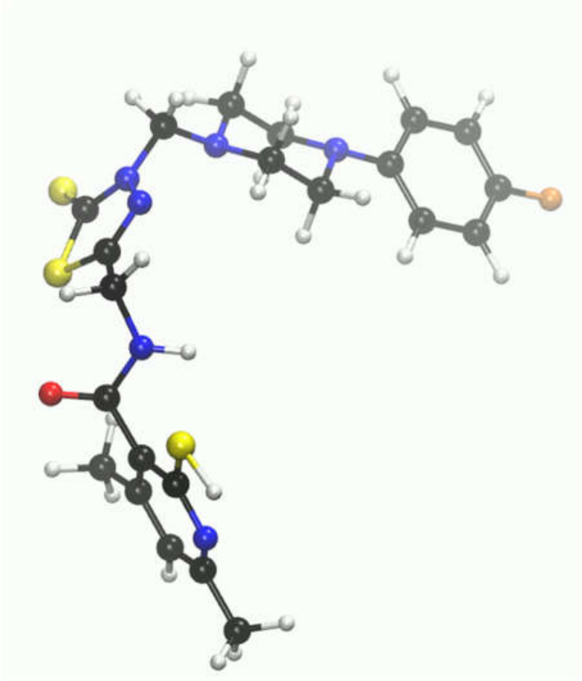

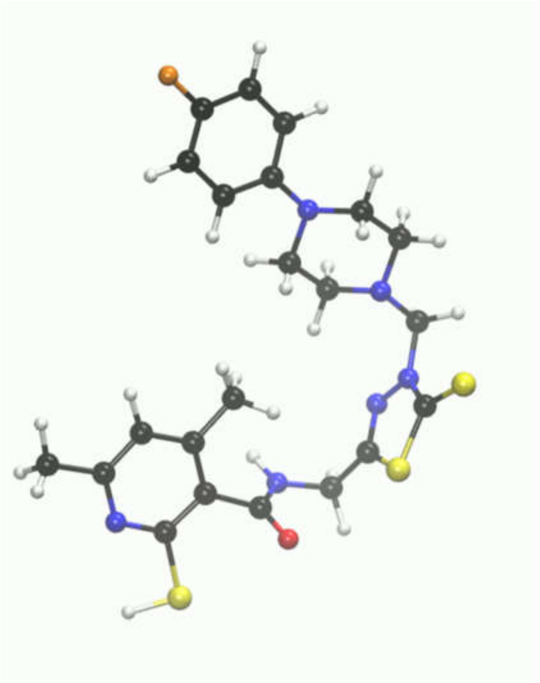

Compound 4

Figure S1 (Continuation). Optimized structures of compounds **2** – **12** obtained as a result of DFT/ωB97XD/6-311+G(d,p) simulations in the gas phase and with solvent reaction field. Color coding: carbon – black, oxygen – red, nitrogen – blue, sulphur – yellow, hydrogen – white, fluorine – orange.

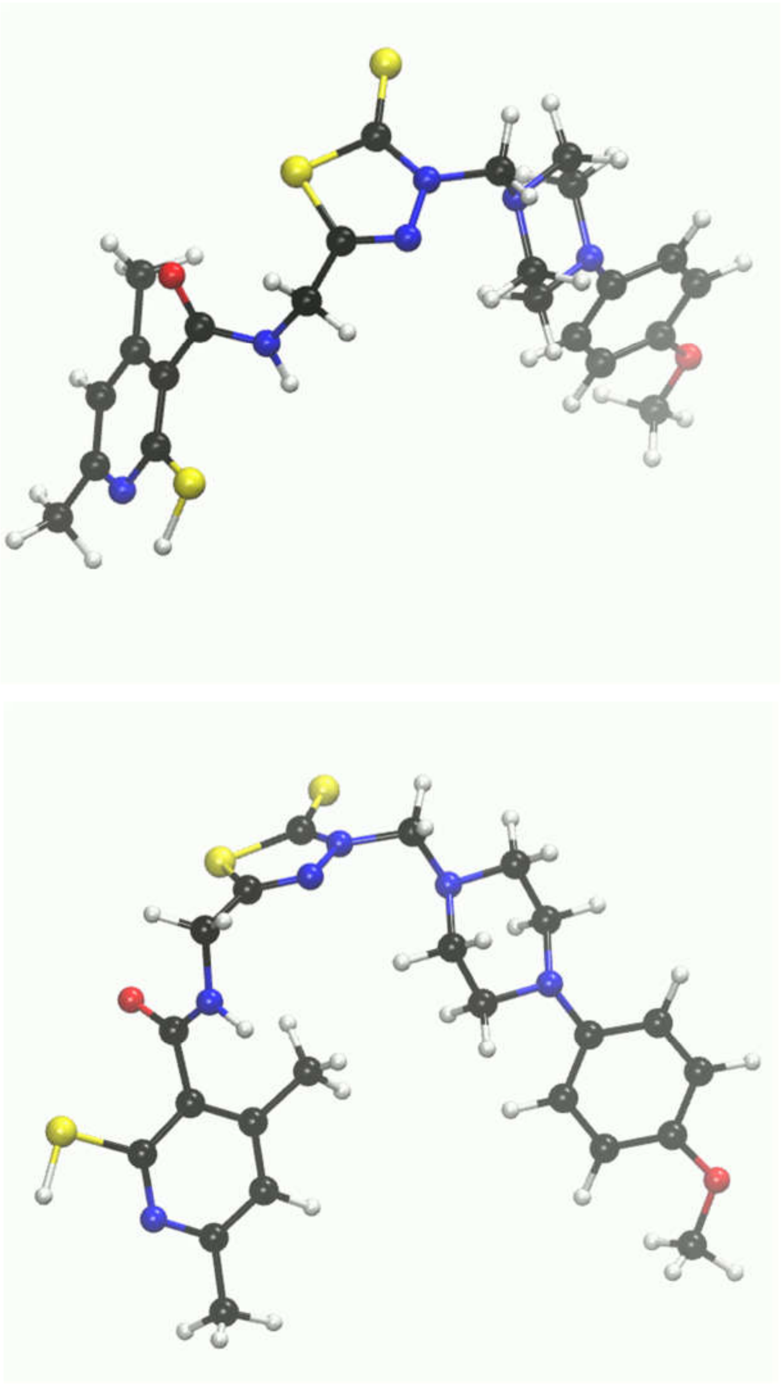

Compound 5

Figure S1 (Continuation). Optimized structures of compounds **2** – **12** obtained as a result of DFT/ωB97XD/6-311+G(d,p) simulations in the gas phase and with solvent reaction field. Color coding: carbon – black, oxygen – red, nitrogen – blue, sulphur – yellow, hydrogen – white.

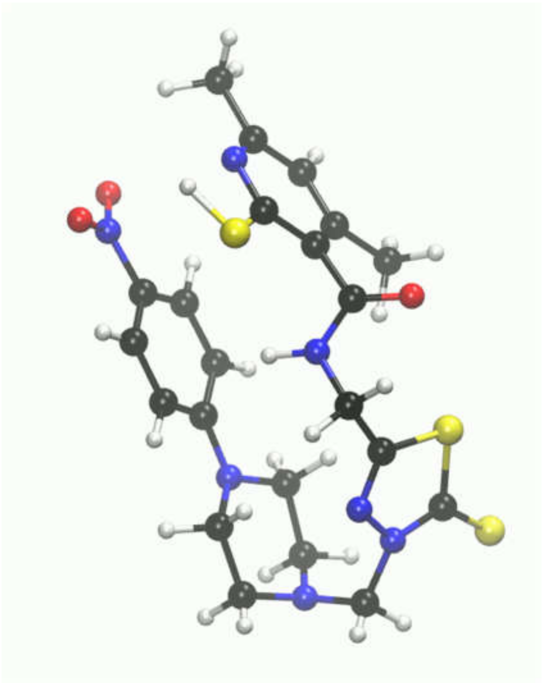

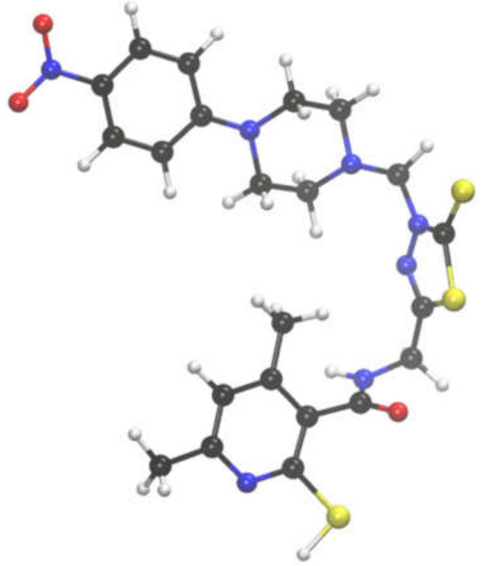

Compound 6

Figure S1 (Continuation). Optimized structures of compounds **2** – **12** obtained as a result of DFT/ωB97XD/6-311+G(d,p) simulations in the gas phase and with solvent reaction field. Color coding: carbon – black, oxygen – red, nitrogen – blue, sulphur – yellow, hydrogen – white.

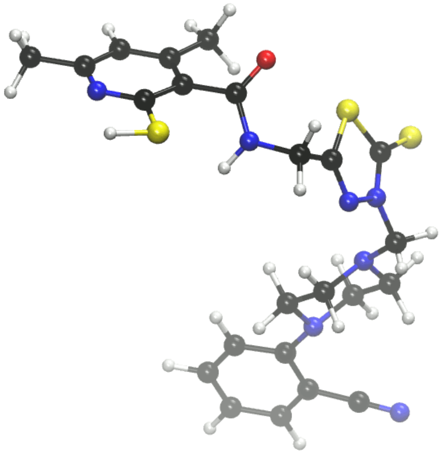

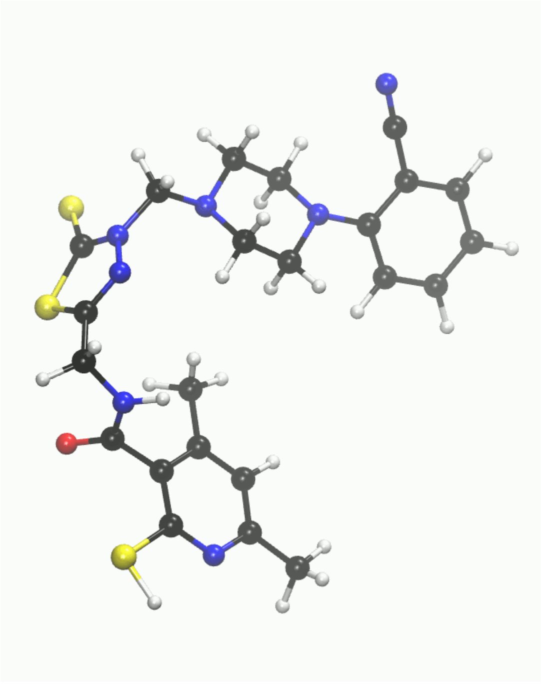

Compound 7

Figure S1 (Continuation). Optimized structures of compounds **2** – **12** obtained as a result of DFT/ωB97XD/6-311+G(d,p) simulations in the gas phase and with solvent reaction field. Color coding: carbon – black, oxygen – red, nitrogen – blue, sulphur – yellow, hydrogen – white.

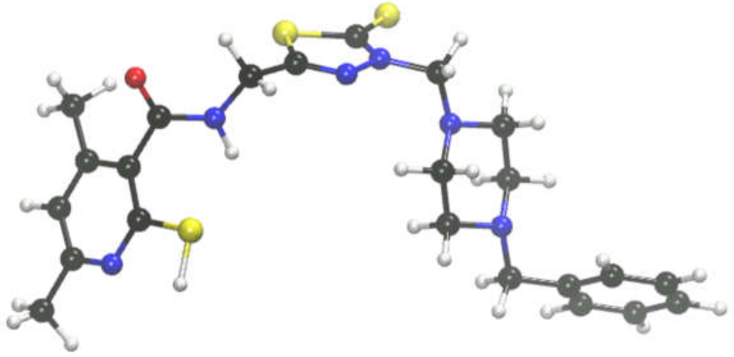

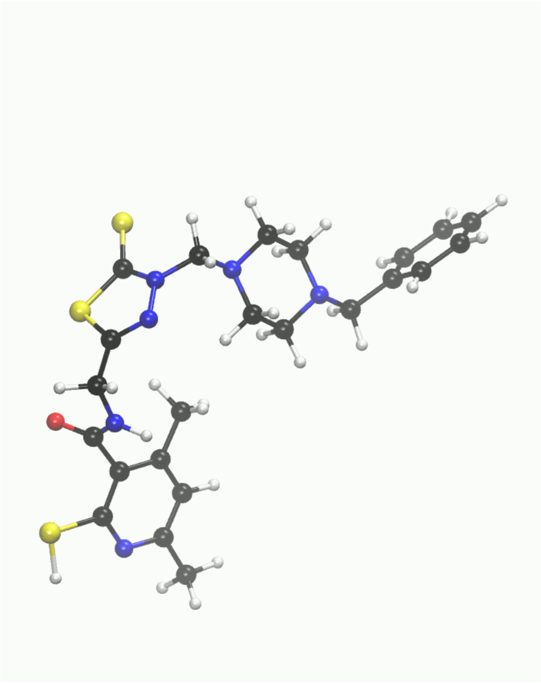

Compound 8

Figure S1 (Continuation). Optimized structures of compounds **2** – **12** obtained as a result of DFT/ωB97XD/6-311+G(d,p) simulations in the gas phase and with solvent reaction field. Color coding: carbon – black, oxygen – red, nitrogen – blue, sulphur – yellow, hydrogen – white.

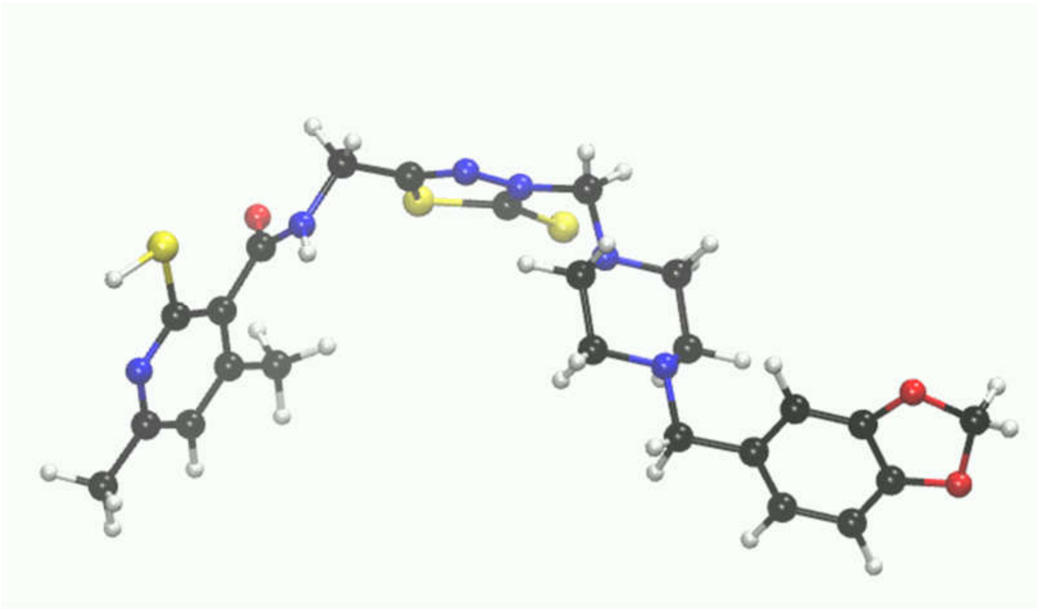

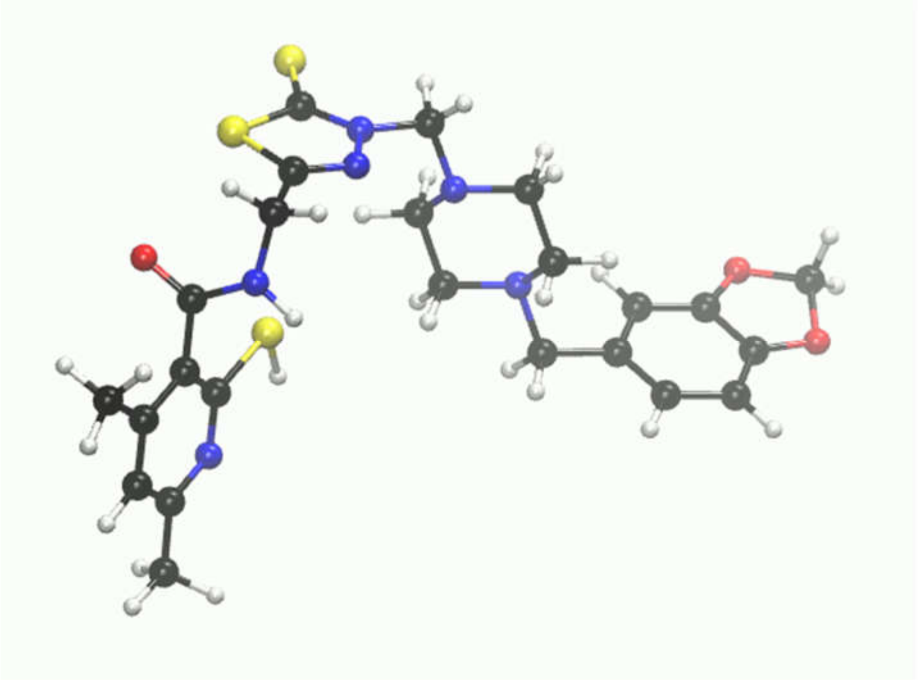

Compound 9

Figure S1 (Continuation). Optimized structures of compounds **2** – **12** obtained as a result of DFT/ωB97XD/6-311+G(d,p) simulations in the gas phase and with solvent reaction field. Color coding: carbon – black, oxygen – red, nitrogen – blue, sulphur – yellow, hydrogen – white.

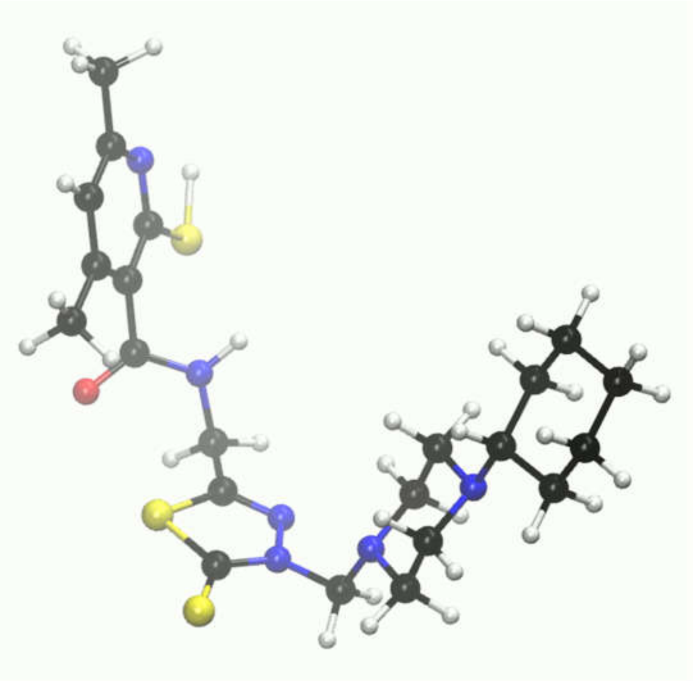

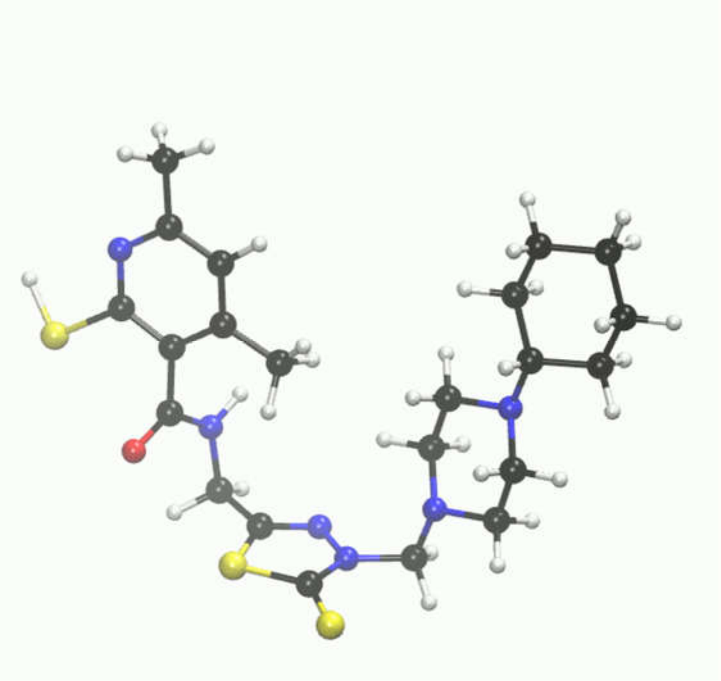

Compound 10

Figure S1 (Continuation). Optimized structures of compounds **2** – **12** obtained as a result of DFT/ωB97XD/6-311+G(d,p) simulations in the gas phase and with solvent reaction field. Color coding: carbon – black, oxygen – red, nitrogen – blue, sulphur – yellow, hydrogen – white.

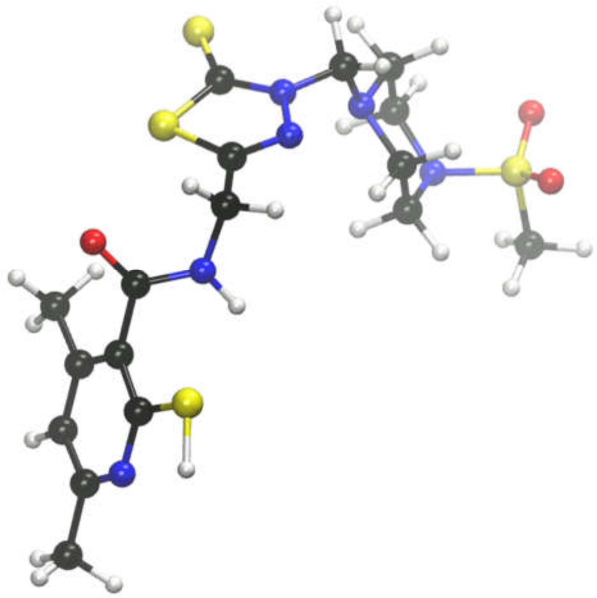

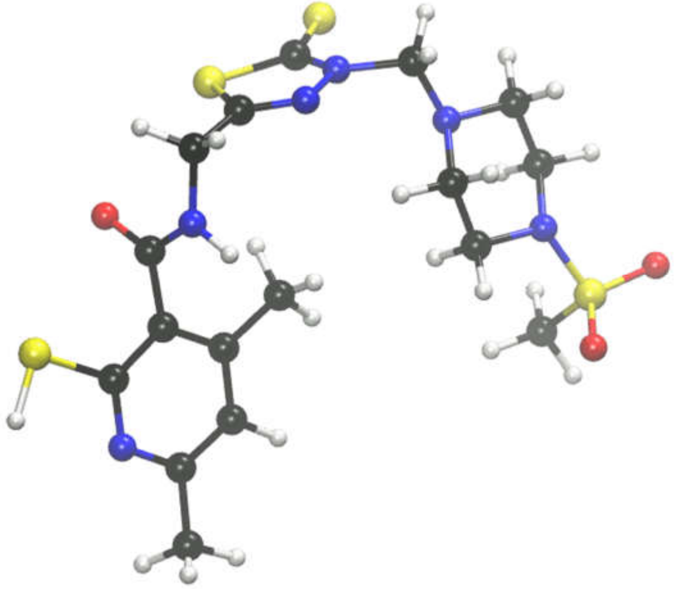

Compound 11

Figure S1 (Continuation). Optimized structures of compounds **2** – **12** obtained as a result of DFT/ωB97XD/6-311+G(d,p) simulations in the gas phase and with solvent reaction field. Color coding: carbon – black, oxygen – red, nitrogen – blue, sulphur – yellow, hydrogen – white.

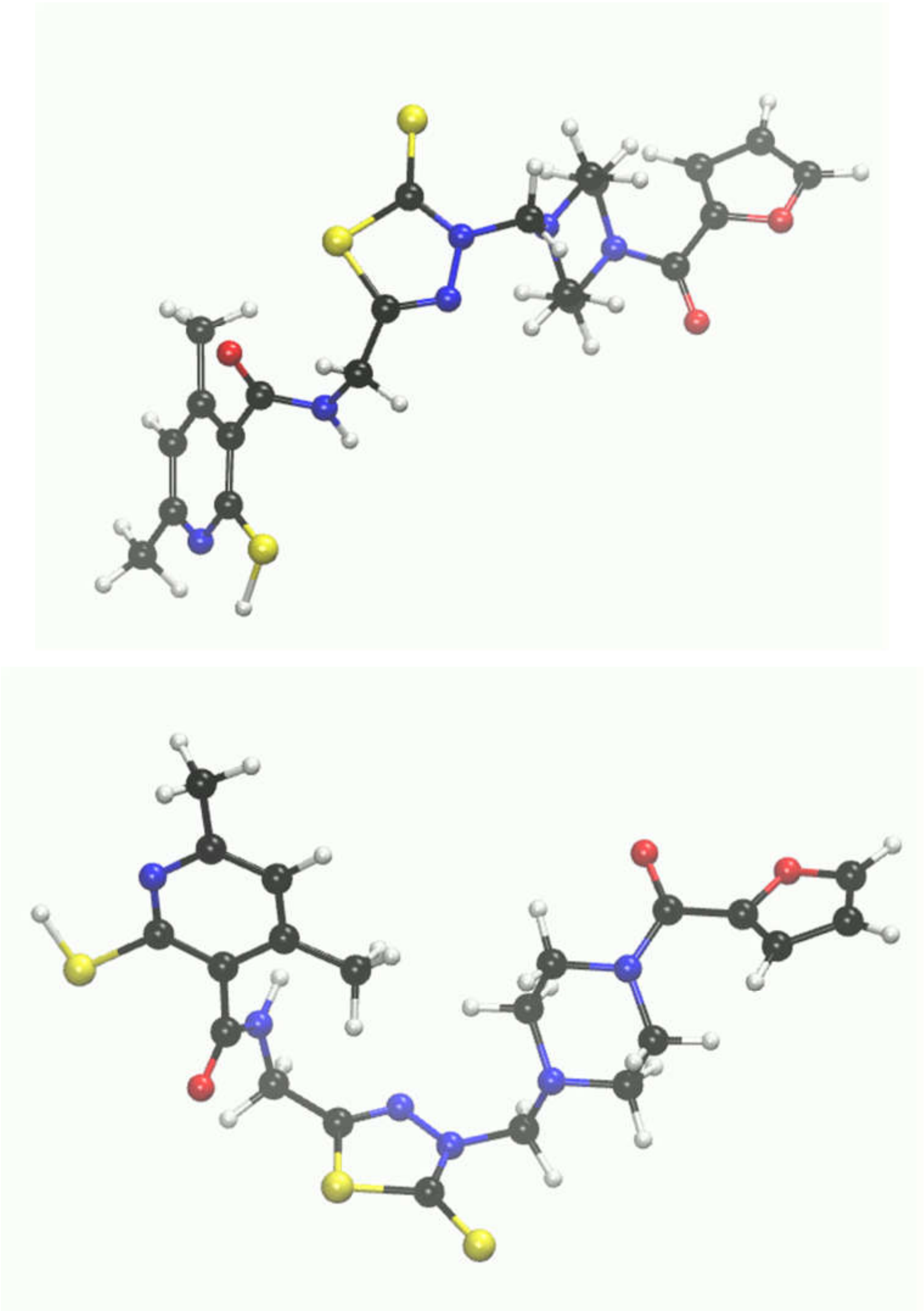

Compound 12

Figure S1 (Continuation). Optimized structures of compounds **2** – **12** obtained as a result of DFT/ωB97XD/6-311+G(d,p) simulations in the gas phase and with solvent reaction field. Color coding: carbon – black, oxygen – red, nitrogen – blue, sulphur – yellow, hydrogen – white.

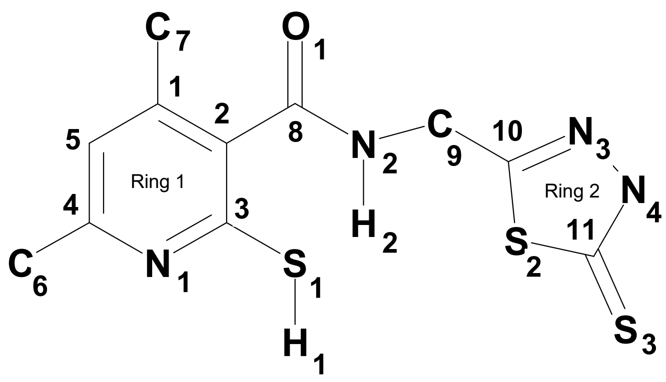

Scheme S1. Core part of the studied compounds. Some hydrogen atoms are omitted for clarity. The atom numbering scheme was prepared especially for the study and QTAIM analyses.

Table S5. Selected metric parameters (see Scheme S1) obtained at the DFT/ωB97XD/6-311+G(d,p) level of theory in the gas phase and using IEF-PCM solvation model (water as a solvent) of three investigated compounds (denoted as **3**, **6**, and **7**).

**Metric parameters**	**Gas phase**	**IEF-PCM/water**
	**Compound 3**	
**Bond in Å**		
**C1-C2**	1.4000	1.3972
**C2-C3**	1.4007	1.4006
**C3-N1**	1.3262	1.3248
**N1-C4**	1.3356	1.3405
**C4-C5**	1.3900	1.3881
**C5-C1**	1.3907	1.3933
**C4-C6**	1.5010	1.5006
**C1-C7**	1.5032	1.5056
**C3-S1**	1.7853	1.7830
**S1-H1**	1.3427	1.3426
**C2-C8**	1.5000	1.5005
**C8-O1**	1.2174	1.2245
**C8-N2**	1.3605	1.3505
**N2-H2**	1.0071	1.0079
**N2-C9**	1.4478	1.4503
**C9-C10**	1.5020	1.5047
**C10-N3**	1.2848	1.2852
**N3-N4**	1.3599	1.3608
**N4-C11**	1.3608	1.3523
**C11-S2**	1.7583	1.7457
**S2-C10**	1.7458	1.7414
**C11-S3**	1.6516	1.6685

Table S5. (Continuation). Selected metric parameters (see Scheme S1) obtained at the DFT/ωB97XD/6-311+G(d,p) level of theory in the gas phase and using IEF-PCM solvation model (water as a solvent) of three investigated compounds (denoted as **3**, **6**, and **7**).

**Valence angle [°]**		
**C3-S1-H1**	93.0	94.0
**C2-C8-O1**	122.7	122.2
**C2-C8-N2**	115.5	115.3
**C8-N2-C9**	121.0	121.9
**N2-C9-C10**	112.5	111.1
**C9-C10-S2**	124.1	124.0
**Dihedral angle [°]**		
**C7-C1-C2-C8**	1.9	1.8
**C1-C2-C8-O1**	55.4	113.7
**C2-C8-N2-C9**	-179.2	174.2
**O1-C8-N2-H2**	-174.1	-178.8
**C8-N2-C9-C10**	-85.3	-79.8
**N2-C9-C10-S2**	67.5	88.1

Table S5. (Continuation). Selected metric parameters (see Scheme S1) obtained at the DFT/ωB97XD/6-311+G(d,p) level of theory in the gas phase and using IEF-PCM solvation model (water as a solvent) of three investigated compounds (denoted as **3**, **6**, and **7**).

**Metric parameters**	**Gas phase**	**IEF-PCM/water**
	**Compound 6**	
**Bond in Å**		
**C1-C2**	1.3993	1.3972
**C2-C3**	1.3995	1.4001
**C3-N1**	1.3264	1.3250
**N1-C4**	1.3353	1.3404
**C4-C5**	1.3915	1.3884
**C5-C1**	1.3892	1.3930
**C4-C6**	1.4990	1.5006
**C1-C7**	1.5032	1.5057
**C3-S1**	1.7832	1.7827
**S1-H1**	1.3434	1.3426
**C2-C8**	1.4969	1.5005
**C8-O1**	1.2199	1.2245
**C8-N2**	1.3585	1.3505
**N2-H2**	1.0107	1.0078
**N2-C9**	1.4471	1.4503
**C9-C10**	1.5032	1.5045
**C10-N3**	1.2837	1.2851
**N3-N4**	1.3599	1.3608
**N4-C11**	1.3611	1.3525
**C11-S2**	1.7575	1.7461
**S2-C10**	1.7458	1.7421
**C11-S3**	1.6566	1.6681

Table S5. (Continuation). Selected metric parameters (see Scheme S1) obtained at the DFT/ωB97XD/6-311+G(d,p) level of theory in the gas phase and using IEF-PCM solvation model (water as a solvent) of three investigated compounds (denoted as **3**, **6**, and **7**).

**Valence angle [°]**		
**C3-S1-H1**	92.9	94.1
**C2-C8-O1**	122.2	122.3
**C2-C8-N2**	116.4	115.1
**C8-N2-C9**	120.0	121.9
**N2-C9-C10**	111.6	111.0
**C9-C10-S2**	124.5	124.1
**Dihedral angle [°]**		
**C7-C1-C2-C8**	5.2	1.7
**C1-C2-C8-O1**	56.2	112.6
**C2-C8-N2-C9**	-179.6	173.1
**O1-C8-N2-H2**	-171.9	-178.6
**C8-N2-C9-C10**	-81.5	-79.6
**N2-C9-C10-S2**	65.5	86.4

Table S5. (Continuation). Selected metric parameters (see Scheme S1) obtained at the DFT/ωB97XD/6-311+G(d,p) level of theory in the gas phase and using IEF-PCM solvation model (water as a solvent) of three investigated compounds (denoted as **3**, **6**, and **7**).

**Metric parameters**	**Gas phase**	**IEF-PCM/water**
	**Compound 7**	
**Bond in Å**		
**C1-C2**	1.4003	1.3974
**C2-C3**	1.4009	1.3963
**C3-N1**	1.3263	1.3264
**N1-C4**	1.3355	1.3394
**C4-C5**	1.3901	1.3905
**C5-C1**	1.3906	1.3915
**C4-C6**	1.5010	1.5009
**C1-C7**	1.5032	1.5043
**C3-S1**	1.7853	1.7815
**S1-H1**	1.3427	1.3423
**C2-C8**	1.5001	1.5025
**C8-O1**	1.2174	1.2234
**C8-N2**	1.3606	1.3519
**N2-H2**	1.0070	1.0087
**N2-C9**	1.4478	1.4507
**C9-C10**	1.5020	1.5044
**C10-N3**	1.2848	1.2853
**N3-N4**	1.3601	1.3605
**N4-C11**	1.3613	1.3522
**C11-S2**	1.7588	1.7467
**S2-C10**	1.7458	1.7413
**C11-S3**	1.6508	1.6680

Table S5. (Continuation). Selected metric parameters (see Scheme S1) obtained at the DFT/ωB97XD/6-311+G(d,p) level of theory in the gas phase and using IEF-PCM solvation model (water as a solvent) of three investigated compounds (denoted as **3**, **6**, and **7**).

**Valence angle [°]**		
**C3-S1-H1**	93.0	94.5
**C2-C8-O1**	122.6	122.6
**C2-C8-N2**	115.6	114.4
**C8-N2-C9**	121.0	122.1
**N2-C9-C10**	112.6	110.0
**C9-C10-S2**	124.1	124.0
**Dihedral angle [°]**		
**C7-C1-C2-C8**	1.9	0.3
**C1-C2-C8-O1**	54.4	97.3
**C2-C8-N2-C9**	-178.8	169.1
**O1-C8-N2-H2**	-174.3	-176.4
**C8-N2-C9-C10**	-85.8	-81.3
**N2-C9-C10-S2**	66.6	87.3

Table S6. Partial atomic charges for selected atoms (see Scheme S1) according to the QTAIM theory (the data are given for the gas phase as well as IEF-PCM results).

**Atomic charge**	**Compound 3**		**Compound 6**		**Compound 7**	
	**Gas phase**	**IEF-PCM**	**Gas phase**	**IEF-PCM**	**Gas phase**	**IEF-PCM**
**qC1**	0.0217	0.0135	0.0208	0.0137	0.0214	0.0160
**qC2**	-0.0367	-0.0348	-0.0314	-0.0350	-0.0381	-0.0379
**qC3**	0.4334	0.4445	0.4348	0.4439	0.4335	0.4391
**qN1**	-1.1826	-1.2009	-1.1762	-1.2007	-1.1826	-1.1994
**qC4**	0.5379	0.5242	0.5431	0.5245	0.5384	0.5265
**qC5**	-0.0317	-0.0319	-0.0360	-0.0305	-0.0320	-0.0309
**qC6**	0.0054	0.0023	0.0019	0.0026	0.0058	0.0024
**qC7**	0.0021	-0.0083	-0.0005	-0.0078	0.0018	-0.0079
**qS1**	0.0599	0.0608	0.0540	0.0602	0.0600	0.0527
**qH1**	0.0233	0.0282	0.0415	0.0290	0.0237	0.0351
**qC8**	1.4470	1.4461	1.4391	1.4466	1.4469	1.4464
**qO1**	-1.1672	-1.1990	-1.1695	-1.1988	-1.1672	-1.1982
**qN2**	-1.1743	-1.1713	-1.1823	-1.1709	-1.1750	-1.1692
**qH2**	0.4140	0.4372	0.4253	0.4372	0.4136	0.4374
**qC9**	0.3986	0.3969	0.4011	0.3966	0.3982	0.3946
**qC10**	0.4884	0.4988	0.4927	0.4989	0.4882	0.4969
**qN3**	-0.7271	-0.7190	-0.7384	-0.7189	-0.7275	-0.7201
**qN4**	-0.7789	-0.7819	-0.7727	-0.7836	-0.7805	-0.7808
**qC11**	0.0069	0.0482	0.0005	0.0473	0.0049	0.0491
**qS2**	0.2865	0.3100	0.3043	0.3101	0.2848	0.3071
**qS3**	0.0874	-0.0381	0.0667	-0.0348	0.0921	-0.0344

Table S7. Electron density at selected Bond and Ring Critical Points (B/RCPs) obtained for compounds denoted as **3**, **6**, and **7** derived from the QTAIM theory. The data are given for the gas phase as well as solvent reaction field (IEF PCM; water as a solvent). For details see Scheme S1.

**Bond**	**Electron density (ρ= e/a_0^3)**					
	**3**		**6**		**7**	
	**Gas phase**	**IEF PCM**	**Gas phase**	**IEF PCM**	**Gas phase**	**IEF PCM**
**C1-C2**	0.3043	0.3049	0.3045	0.3049	0.3041	0.3050
**C2-C3**	0.3028	0.3035	0.3033	0.3038	0.3027	0.3054
**C3-N1**	0.3494	0.3499	0.3495	0.3498	0.3493	0.3489
**N1-C4**	0.3380	0.3344	0.3381	0.3345	0.3381	0.3349
**C4-C5**	0.3134	0.3144	0.3127	0.3143	0.3134	0.3131
**C5-C1**	0.3115	0.3101	0.3121	0.3102	0.3116	0.3108
**C4-C6**	0.2576	0.2576	0.2583	0.2577	0.2576	0.2575
**C1-C7**	0.2530	0.2519	0.2531	0.2518	0.2530	0.2525
**C3-S1**	0.1923	0.1932	0.1929	0.1932	0.1922	0.1937
**S1-H1**	0.2183	0.2185	0.2184	0.2185	0.2184	0.2184
**C2-C8**	0.2615	0.2620	0.2627	0.2620	0.2614	0.2613
**C8-O1**	0.4069	0.3998	0.4045	0.3998	0.4069	0.4006
**C8-N2**	0.3182	0.3256	0.3199	0.3256	0.3181	0.3249
**N2-H2**	0.3408	0.3404	0.3369	0.3405	0.3409	0.3399
**N2-C9**	0.2637	0.2623	0.2653	0.2624	0.2636	0.2622
**C9-C10**	0.2565	0.2547	0.2557	0.2548	0.2565	0.2549
**C10-N3**	0.3722	0.3723	0.3729	0.3723	0.3723	0.3723
**N3-N4**	0.3590	0.3584	0.3588	0.3583	0.3588	0.3586
**N4-C11**	0.3161	0.3216	0.3158	0.3214	0.3159	0.3217
**C11-S2**	0.2032	0.2077	0.2038	0.2075	0.2030	0.2073
**S2-C10**	0.2054	0.2074	0.2056	0.2072	0.2054	0.2074
**C11-S3**	0.2147	0.2104	0.2130	0.2106	0.2149	0.2106
**Ring**						
**Ring 1**	0.0242	0.0242	0.0244	0.0242	0.0242	0.0242
**Ring 2**	0.0416	0.0421	0.0420	0.0421	0.0415	0.0421

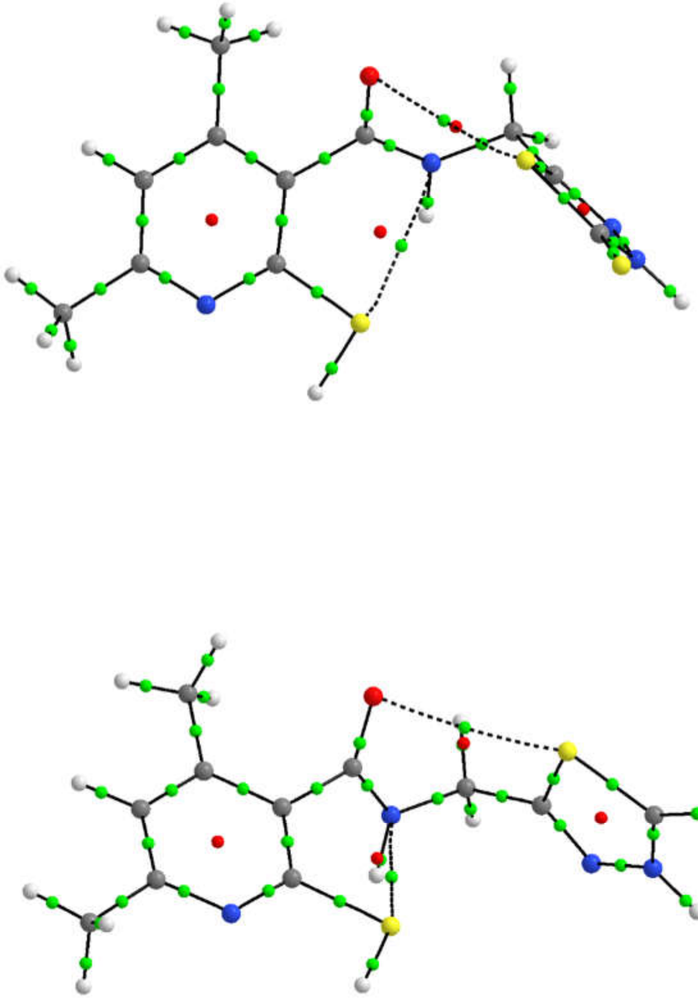

Compound 2

Figure S2. QTAIM molecular graphs of compounds **2** – **12** obtained as a result of the DFT/ωB97XD/6-311+G(d,p) simulations in the gas phase (two presentations to show the detected network of noncovalent interactions). Color coding: carbon – grey, oxygen – red, nitrogen – blue, sulphur – yellow, hydrogen – white, fluorine – green (compound **4**). Dotted lines indicate the network of detected noncovalent interactions. Green and red spheres indicate the presence of Bond and Ring Critical Points (BCPs and RCPs), respectively.

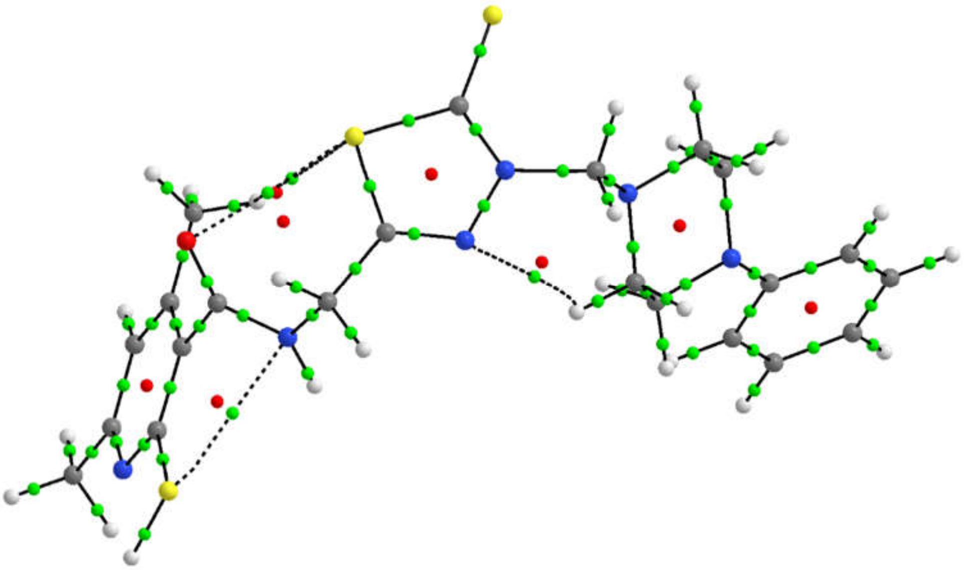

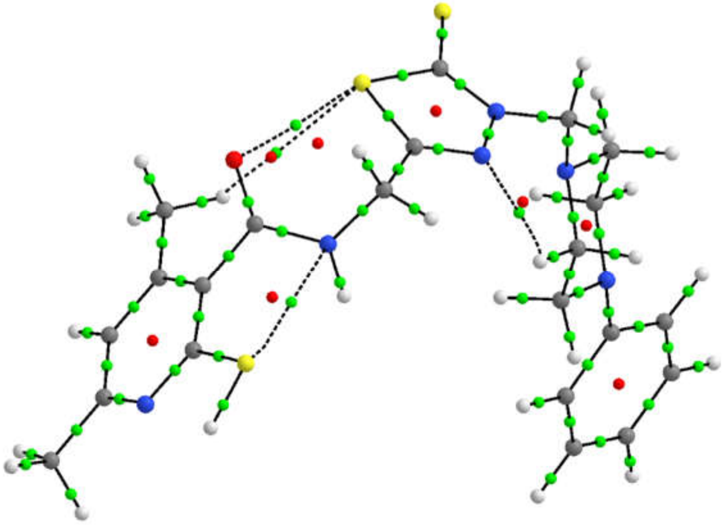

Compound 3

Figure S2 (Continuation). QTAIM molecular of compounds **2** – **12** obtained as a result of the DFT/ωB97XD/6-311+G(d,p) simulations in the gas phase (two presentations to show the detected network of noncovalent interactions). Color coding: carbon – grey, oxygen – red, nitrogen – blue, sulphur – yellow, hydrogen – white, fluorine – green (compound **4**). Dotted lines indicate the network of detected noncovalent interactions. Green and red spheres indicate the presence of Bond and Ring Critical Points (BCPs and RCPs), respectively.

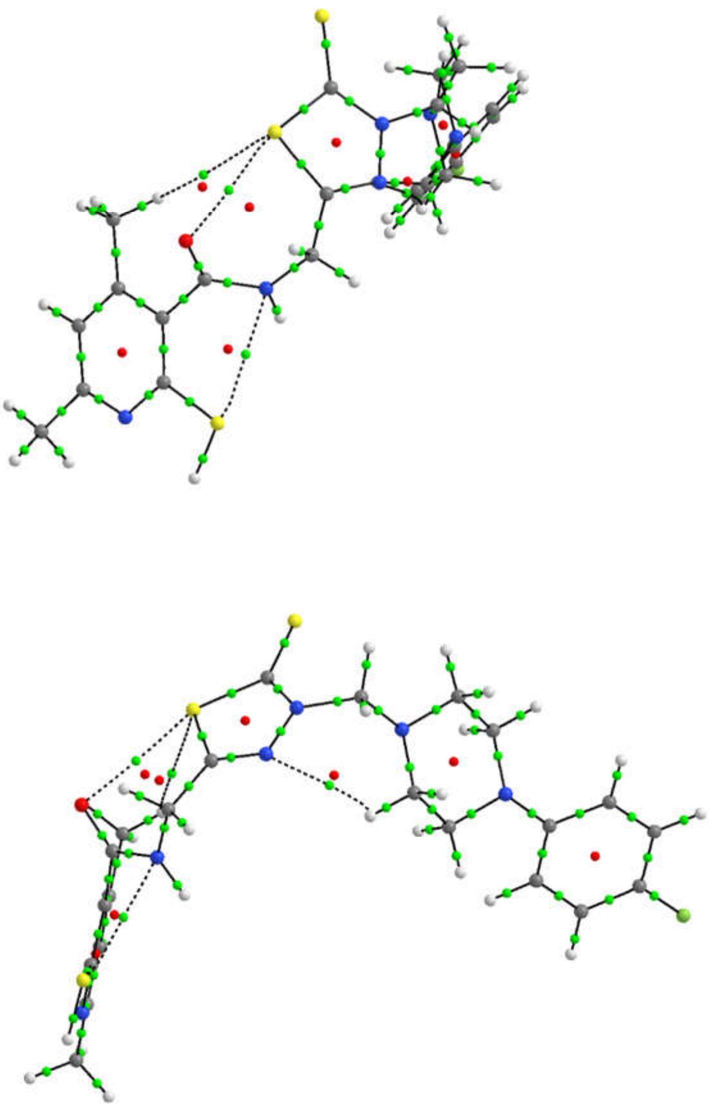

Compound 4

Figure S2 (Continuation). QTAIM molecular graphs of compounds **2** – **12** obtained as a result of the DFT/ωB97XD/6-311+G(d,p) simulations in the gas phase (two presentations to show the detected network of noncovalent interactions). Color coding: carbon – grey, oxygen – red, nitrogen – blue, sulphur – yellow, hydrogen – white, fluorine – green (compound **4**). Dotted lines indicate the network of detected noncovalent interactions. Green and red spheres indicate the presence of Bond and Ring Critical Points (BCPs and RCPs), respectively.

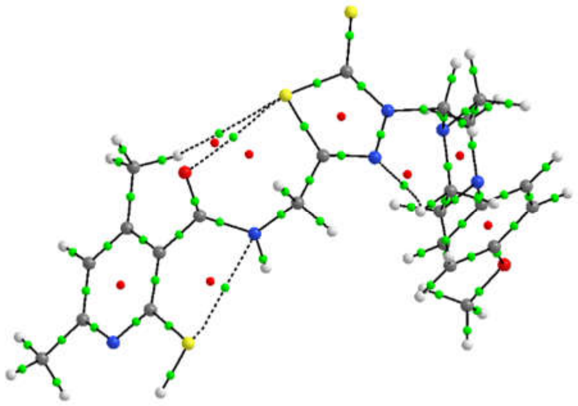

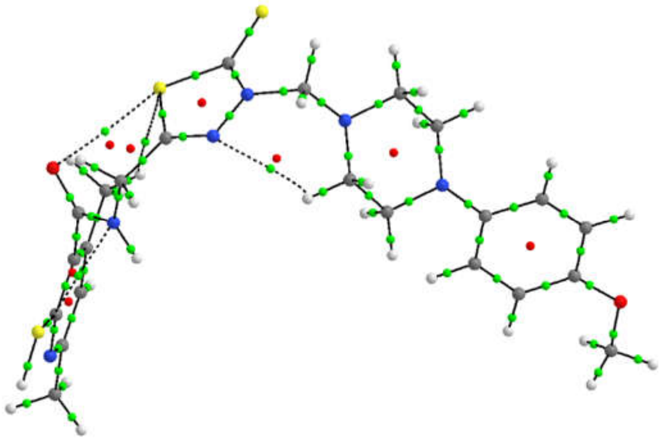

Compound 5

Figure S2 (Continuation). QTAIM molecular graphs of compounds **2** – **12** obtained as a result of the DFT/ωB97XD/6-311+G(d,p) simulations in the gas phase (two presentations to show the detected network of noncovalent interactions). Color coding: carbon – grey, oxygen – red, nitrogen – blue, sulphur – yellow, hydrogen – white, fluorine – green (compound **4**). Dotted lines indicate the network of detected noncovalent interactions. Green and red spheres indicate the presence of Bond and Ring Critical Points (BCPs and RCPs), respectively.

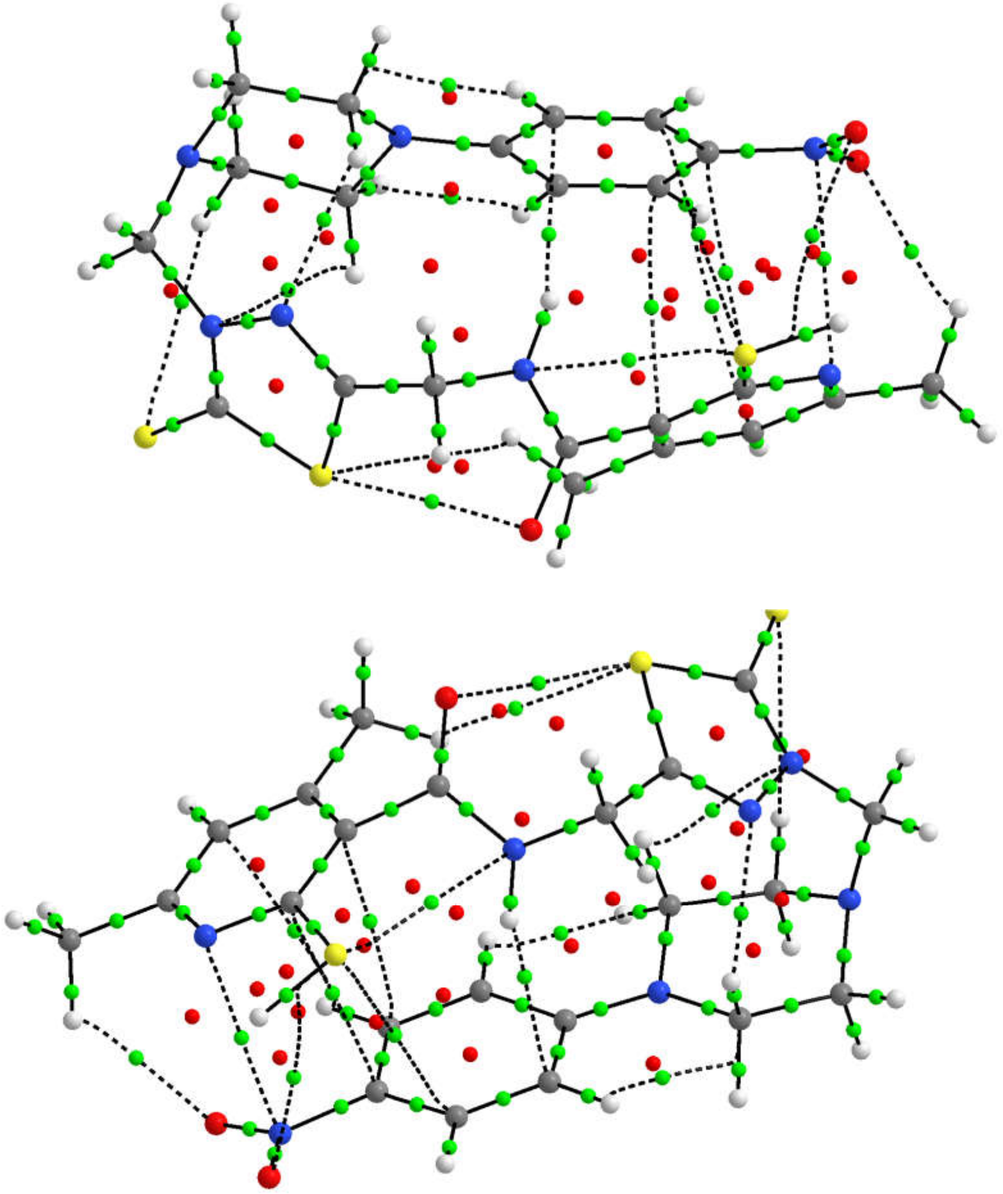

Compound 6

Figure S2 (Continuation). QTAIM molecular graphs of compounds **2** – **12** obtained as a result of the DFT/ωB97XD/6-311+G(d,p) simulations in the gas phase (two presentations to show the detected network of noncovalent interactions). Color coding: carbon – grey, oxygen – red, nitrogen – blue, sulphur – yellow, hydrogen – white, fluorine – green (compound **4**). Dotted lines indicate the network of detected noncovalent interactions. Green and red spheres indicate the presence of Bond and Ring Critical Points (BCPs and RCPs), respectively.

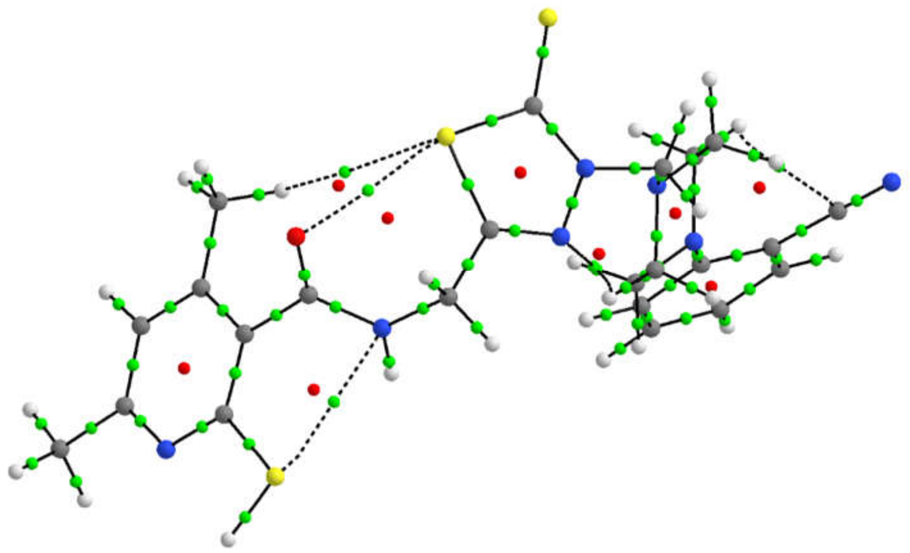

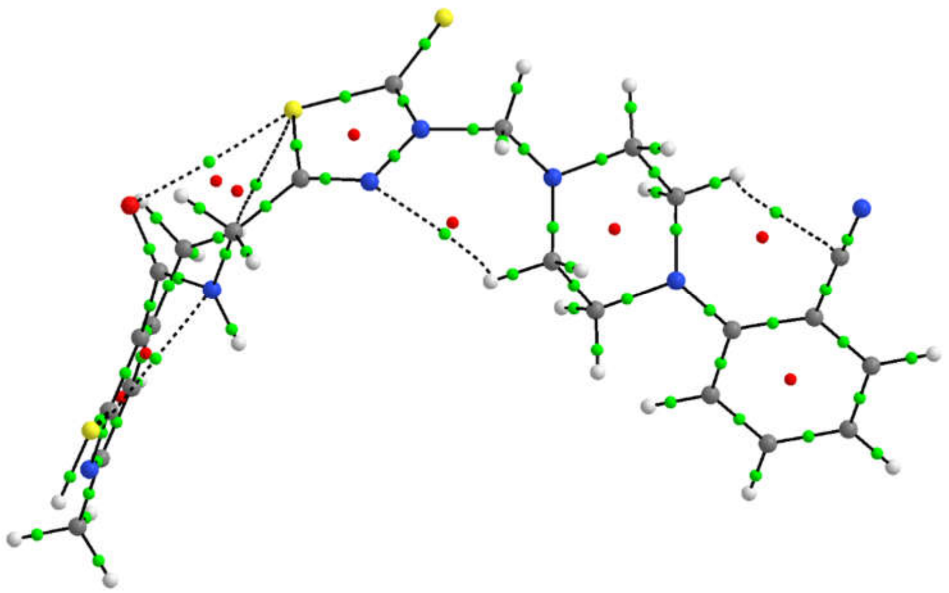

Compound 7

Figure S2 (Continuation). QTAIM molecular graphs of compounds **2** – **12** obtained as a result of the DFT/ωB97XD/6-311+G(d,p) simulations in the gas phase (two presentations to show the detected network of noncovalent interactions). Color coding: carbon – grey, oxygen – red, nitrogen – blue, sulphur – yellow, hydrogen – white, fluorine – green (compound **4**). Dotted lines indicate the network of detected noncovalent interactions. Green and red spheres indicate the presence of Bond and Ring Critical Points (BCPs and RCPs), respectively.

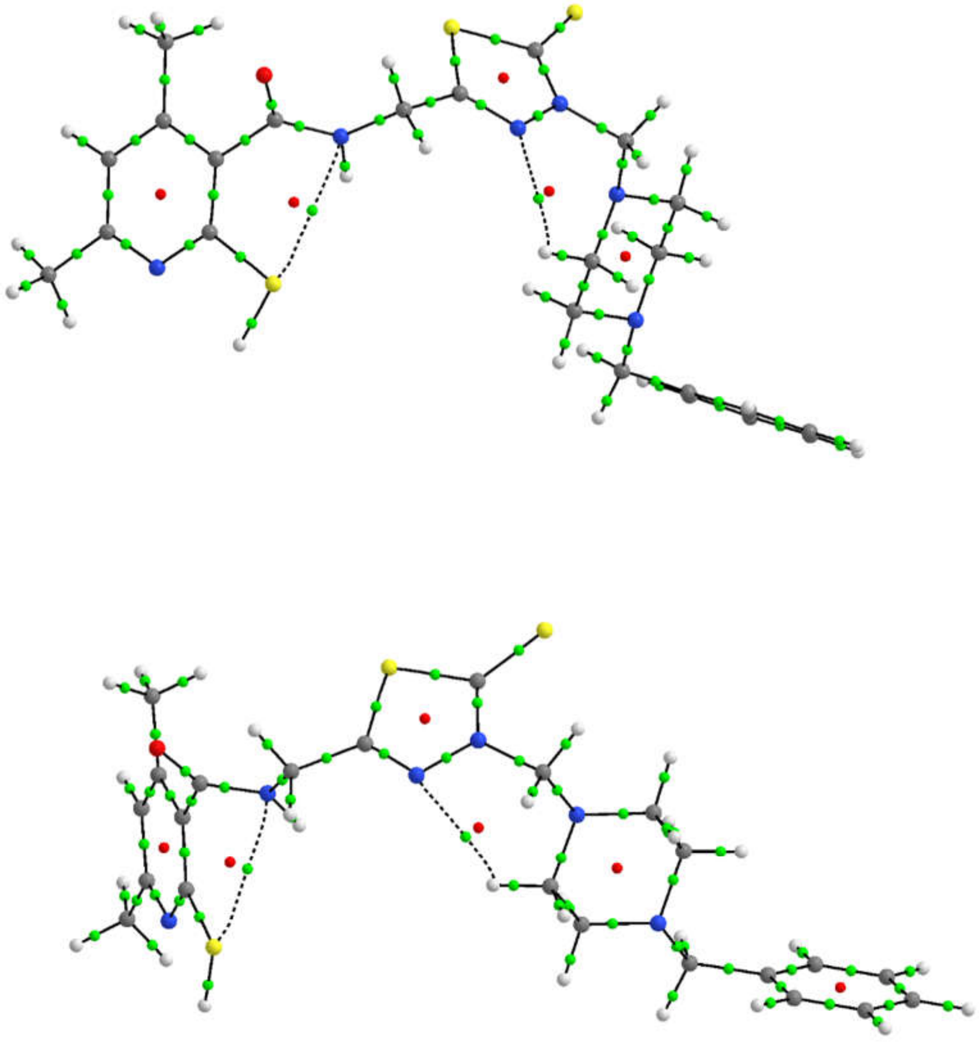

Compound 8

Figure S2 (Continuation). QTAIM molecular graphs of compounds **2** – **12** obtained as a result of the DFT/ωB97XD/6-311+G(d,p) simulations in the gas phase (two presentations to show the detected network of noncovalent interactions). Color coding: carbon – grey, oxygen – red, nitrogen – blue, sulphur – yellow, hydrogen – white, fluorine – green (compound **4**). Dotted lines indicate the network of detected noncovalent interactions. Green and red spheres indicate the presence of Bond and Ring Critical Points (BCPs and RCPs), respectively.

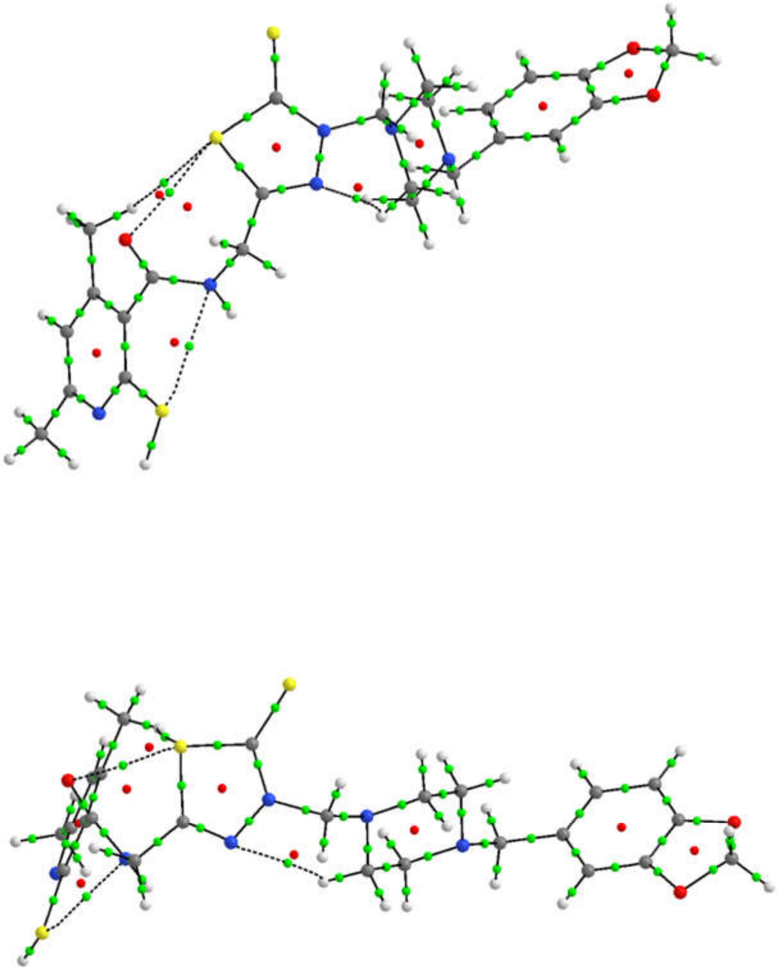

Compound 9

Figure S2 (Continuation). QTAIM molecular graphs of compounds **2** – **12** obtained as a result of the DFT/ωB97XD/6-311+G(d,p) simulations in the gas phase (two presentations to show the detected network of noncovalent interactions). Color coding: carbon – grey, oxygen – red, nitrogen – blue, sulphur – yellow, hydrogen – white, fluorine – green (compound **4**). Dotted lines indicate the network of detected noncovalent interactions. Green and red spheres indicate the presence of Bond and Ring Critical Points (BCPs and RCPs), respectively.

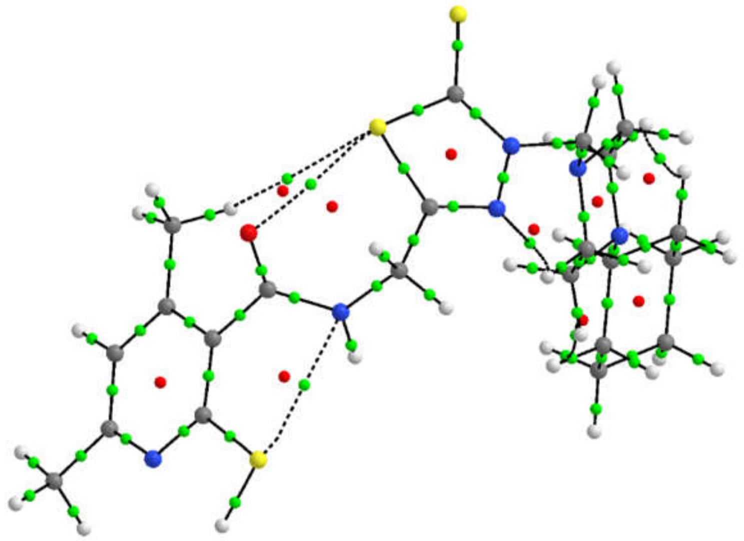

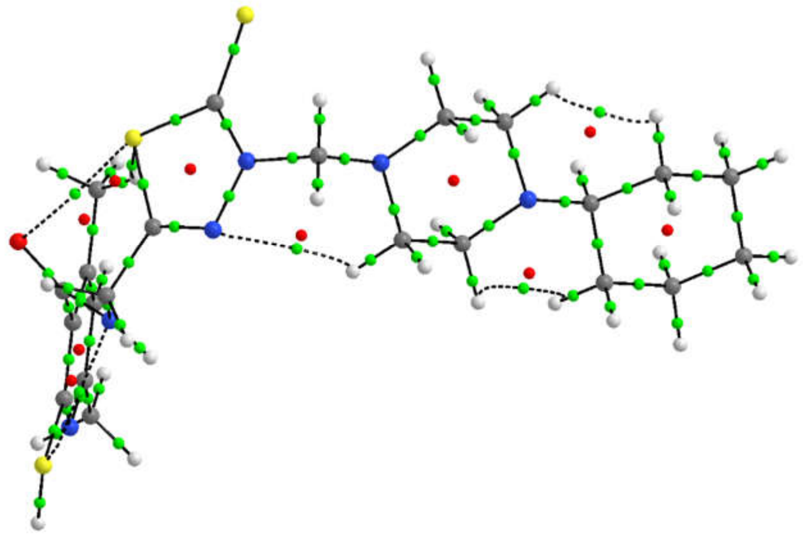

Compound 10

Figure S2 (Continuation). QTAIM molecular graphs of compounds **2** – **12** obtained as a result of the DFT/ωB97XD/6-311+G(d,p) simulations in the gas phase (two presentations to show the detected network of noncovalent interactions). Color coding: carbon – grey, oxygen – red, nitrogen – blue, sulphur – yellow, hydrogen – white, fluorine – green (compound **4**). Dotted lines indicate the network of detected noncovalent interactions. Green and red spheres indicate the presence of Bond and Ring Critical Points (BCPs and RCPs), respectively.

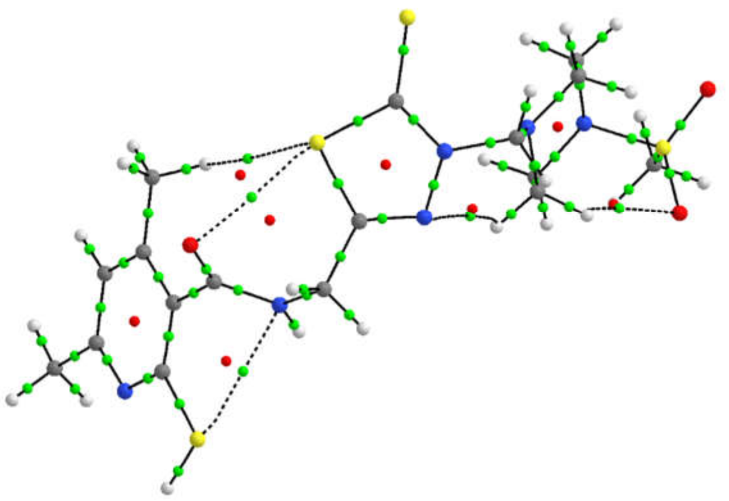

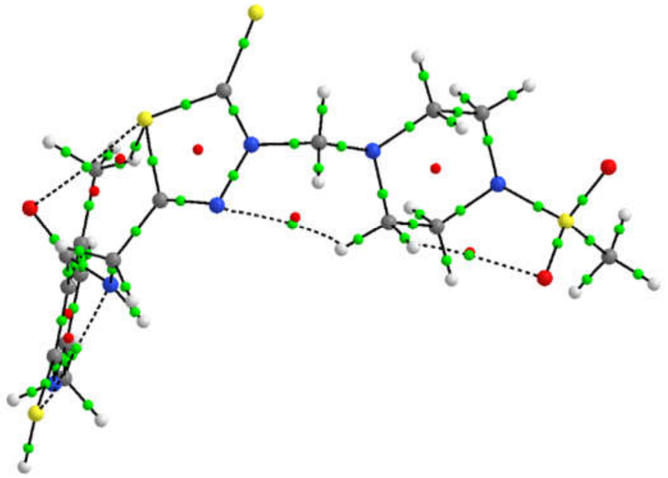

Compound 11

Figure S2 (Continuation). QTAIM molecular graphs of compounds **2** – **12** obtained as a result of the DFT/ωB97XD/6-311+G(d,p) simulations in the gas phase (two presentations to show the detected network of noncovalent interactions). Color coding: carbon – grey, oxygen – red, nitrogen – blue, sulphur – yellow, hydrogen – white, fluorine – green (compound **4**). Dotted lines indicate the network of detected noncovalent interactions. Green and red spheres indicate the presence of Bond and Ring Critical Points (BCPs and RCPs), respectively.

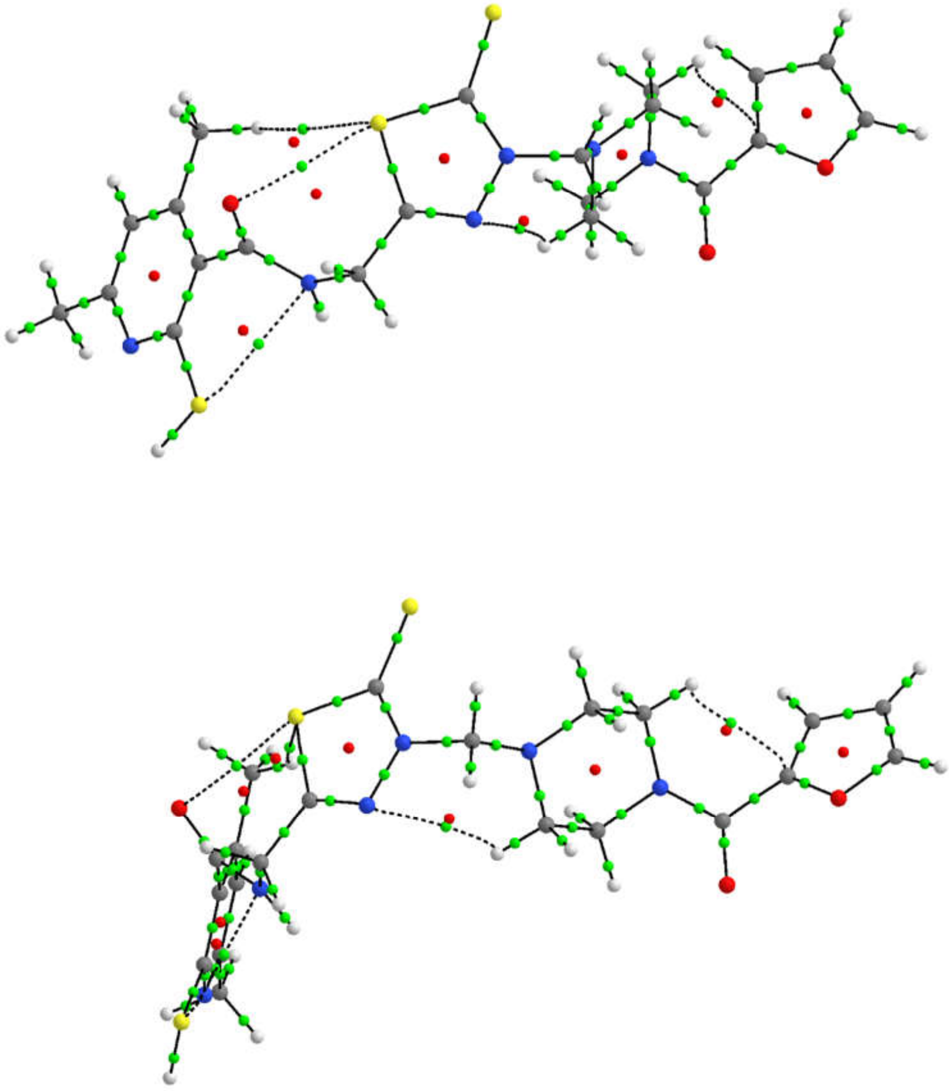

Compound 12

Figure S2 (Continuation). QTAIM molecular graphs of compounds **2** – **12** obtained as a result of the DFT/ωB97XD/6-311+G(d,p) simulations in the gas phase (two presentations to show the detected network of noncovalent interactions). Color coding: carbon – grey, oxygen – red, nitrogen – blue, sulphur – yellow, hydrogen – white, fluorine – green (compound **4**). Dotted lines indicate the network of detected noncovalent interactions. Green and red spheres indicate the presence of Bond and Ring Critical Points (BCPs and RCPs), respectively.

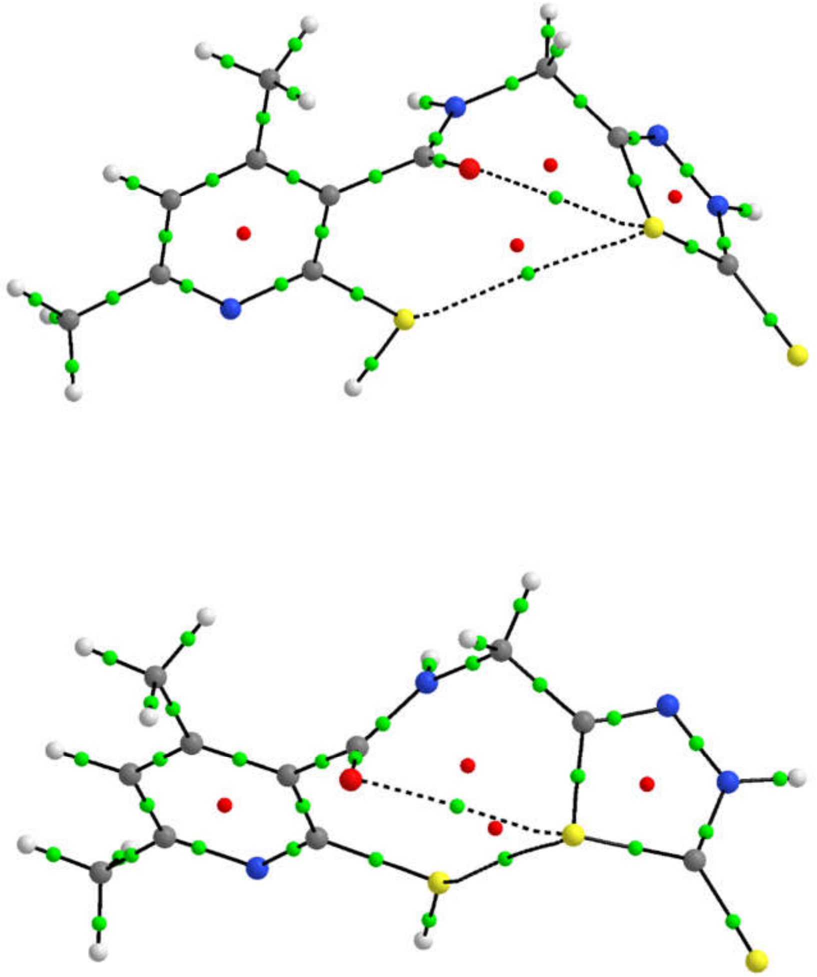

Compound 2

Figure S3. QTAIM molecular graphs of compounds **2** – **12** obtained as a result of the DFT/ωB97XD/6-311+G(d,p) simulations with solvent reaction field IEF-PCM and water as a solvent (two presentations to show the detected network of noncovalent interactions). Color coding: carbon – grey, oxygen – red, nitrogen – blue, sulphur – yellow, hydrogen – white, fluorine – green (compound **4**). Dotted lines indicate the network noncovalent interactions. Green and red spheres indicate the presence of Bond and Ring Critical Points (BCPs and RCPs), respectively.

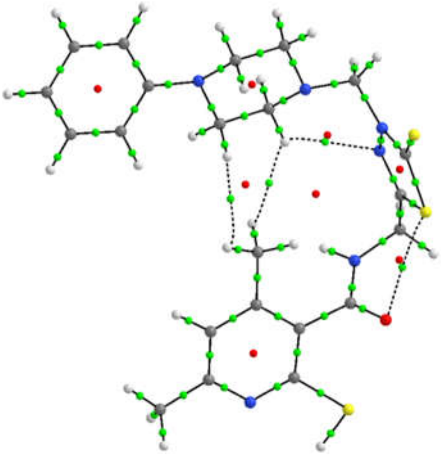

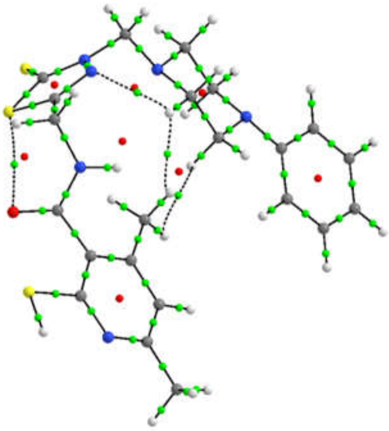

Compound 3

Figure S3 (Continuation). QTAIM molecular graphs of compounds **2** – **12** obtained as a result of the DFT/ωB97XD/6-311+G(d,p) simulations with solvent reaction field IEF-PCM and water as a solvent (two presentations to show the detected network of noncovalent interactions). Color coding: carbon – grey, oxygen – red, nitrogen – blue, sulphur – yellow, hydrogen – white, fluorine – green (compound **4**). Dotted lines indicate the network noncovalent interactions. Green and red spheres indicate the presence of Bond and Ring Critical Points (BCPs and RCPs), respectively.

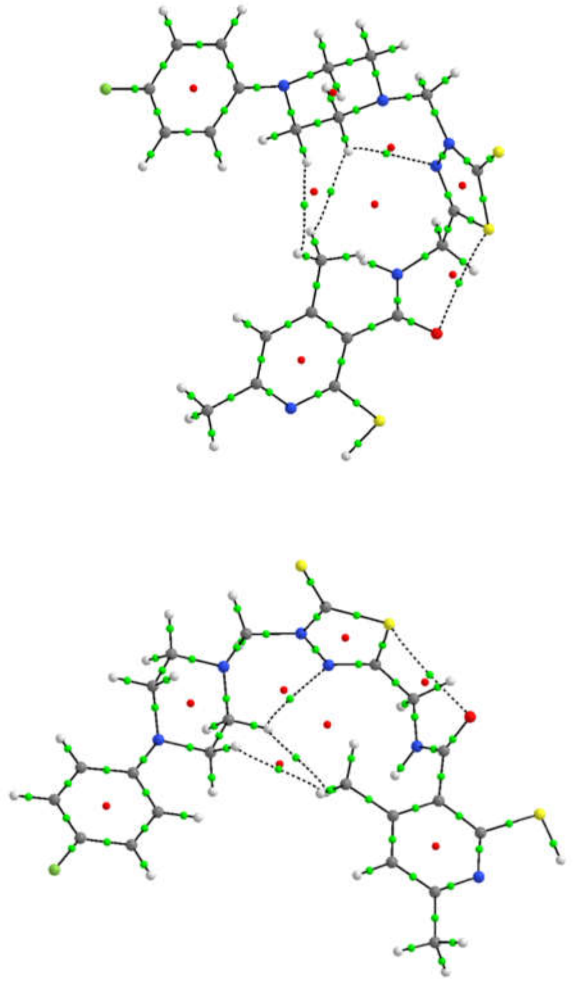

Compound 4

Figure S3 (Continuation). QTAIM molecular graphs of compounds **2** – **12** obtained as a result of the DFT/ωB97XD/6-311+G(d,p) simulations with solvent reaction field IEF-PCM and water as a solvent (two presentations to show the detected network of noncovalent interactions). Color coding: carbon – grey, oxygen – red, nitrogen – blue, sulphur – yellow, hydrogen – white, fluorine – green (compound **4**). Dotted lines indicate the network noncovalent interactions. Green and red spheres indicate the presence of Bond and Ring Critical Points (BCPs and RCPs), respectively.

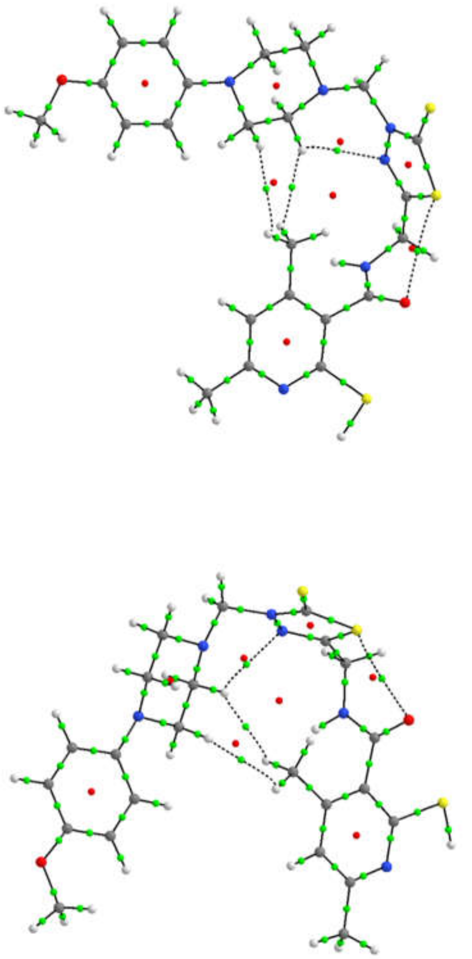

Compound 5

Figure S3 (Continuation). QTAIM molecular graphs of compounds **2** – **12** obtained as a result of the DFT/ωB97XD/6-311+G(d,p) simulations with solvent reaction field IEF-PCM and water as a solvent (two presentations to show the detected network of noncovalent interactions). Color coding: carbon – grey, oxygen – red, nitrogen – blue, sulphur – yellow, hydrogen – white, fluorine – green (compound **4**). Dotted lines indicate the network noncovalent interactions. Green and red spheres indicate the presence of Bond and Ring Critical Points (BCPs and RCPs), respectively.

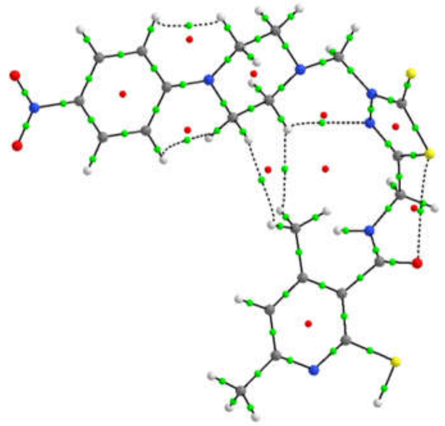

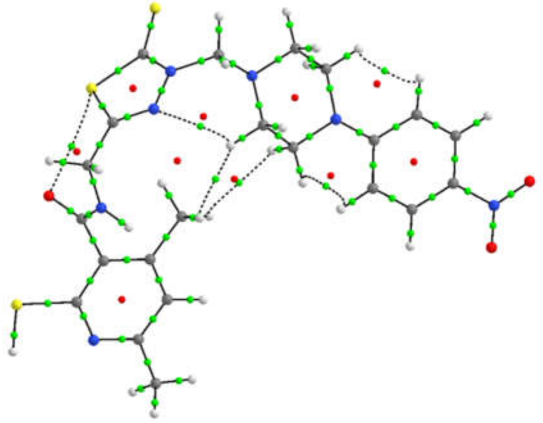

Compound 6

Figure S3 (Continuation). QTAIM molecular graphs of compounds **2** – **12** obtained as a result of the DFT/ωB97XD/6-311+G(d,p) simulations with solvent reaction field IEF-PCM and water as a solvent (two presentations to show the detected network of noncovalent interactions). Color coding: carbon – grey, oxygen – red, nitrogen – blue, sulphur – yellow, hydrogen – white, fluorine – green (compound **4**). Dotted lines indicate the network noncovalent interactions. Green and red spheres indicate the presence of Bond and Ring Critical Points (BCPs and RCPs), respectively.

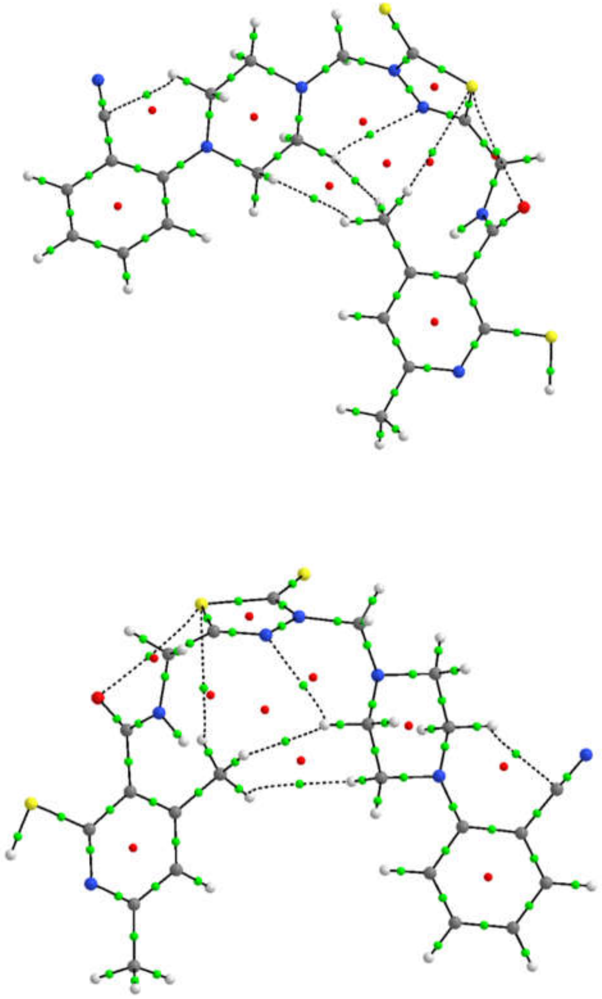

Compound 7

Figure S3 (Continuation). QTAIM molecular graphs of compounds **2** – **12** obtained as a result of the DFT/ωB97XD/6-311+G(d,p) simulations with solvent reaction field IEF-PCM and water as a solvent (two presentations to show the detected network of noncovalent interactions). Color coding: carbon – grey, oxygen – red, nitrogen – blue, sulphur – yellow, hydrogen – white, fluorine – green (compound **4**). Dotted lines indicate the network noncovalent interactions. Green and red spheres indicate the presence of Bond and Ring Critical Points (BCPs and RCPs), respectively.

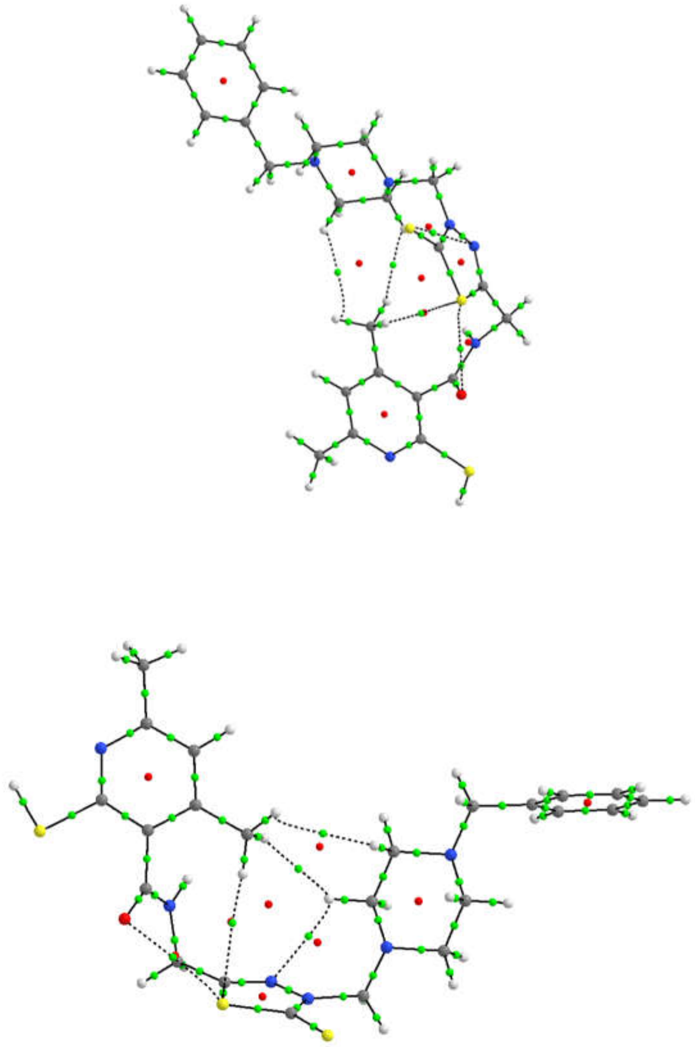

Compound 8

Figure S3 (Continuation). QTAIM molecular graphs of compounds **2** – **12** obtained as a result of the DFT/ωB97XD/6-311+G(d,p) simulations with solvent reaction field IEF-PCM and water as a solvent (two presentations to show the detected network of noncovalent interactions). Color coding: carbon – grey, oxygen – red, nitrogen – blue, sulphur – yellow, hydrogen – white, fluorine – green (compound **4**). Dotted lines indicate the network noncovalent interactions. Green and red spheres indicate the presence of Bond and Ring Critical Points (BCPs and RCPs), respectively.

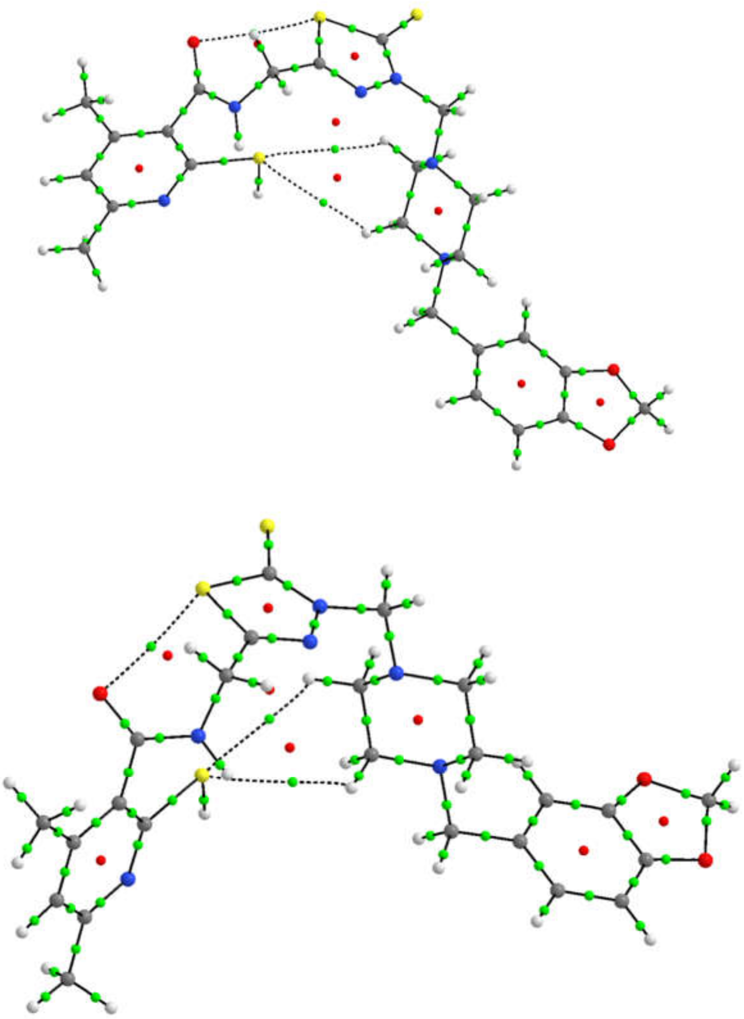

Compound 9

Figure S3 (Continuation). QTAIM molecular graphs of compounds **2** – **12** obtained as a result of the DFT/ωB97XD/6-311+G(d,p) simulations with solvent reaction field IEF-PCM and water as a solvent (two presentations to show the detected network of noncovalent interactions). Color coding: carbon – grey, oxygen – red, nitrogen – blue, sulphur – yellow, hydrogen – white, fluorine – green (compound **4**). Dotted lines indicate the network noncovalent interactions. Green and red spheres indicate the presence of Bond and Ring Critical Points (BCPs and RCPs), respectively.

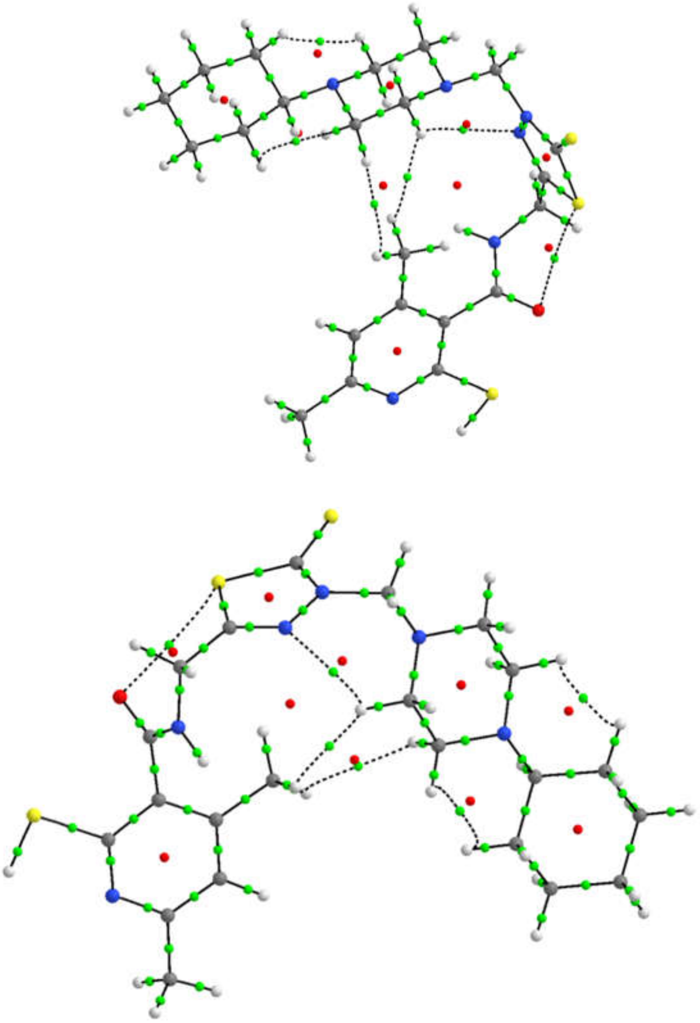

Compound 10

Figure S3 (Continuation). QTAIM molecular graphs of compounds **2** – **12** obtained as a result of the DFT/ωB97XD/6-311+G(d,p) simulations with solvent reaction field IEF-PCM and water as a solvent (two presentations to show the detected network of noncovalent interactions). Color coding: carbon – grey, oxygen – red, nitrogen – blue, sulphur – yellow, hydrogen – white, fluorine – green (compound **4**). Dotted lines indicate the network noncovalent interactions. Green and red spheres indicate the presence of Bond and Ring Critical Points (BCPs and RCPs), respectively.

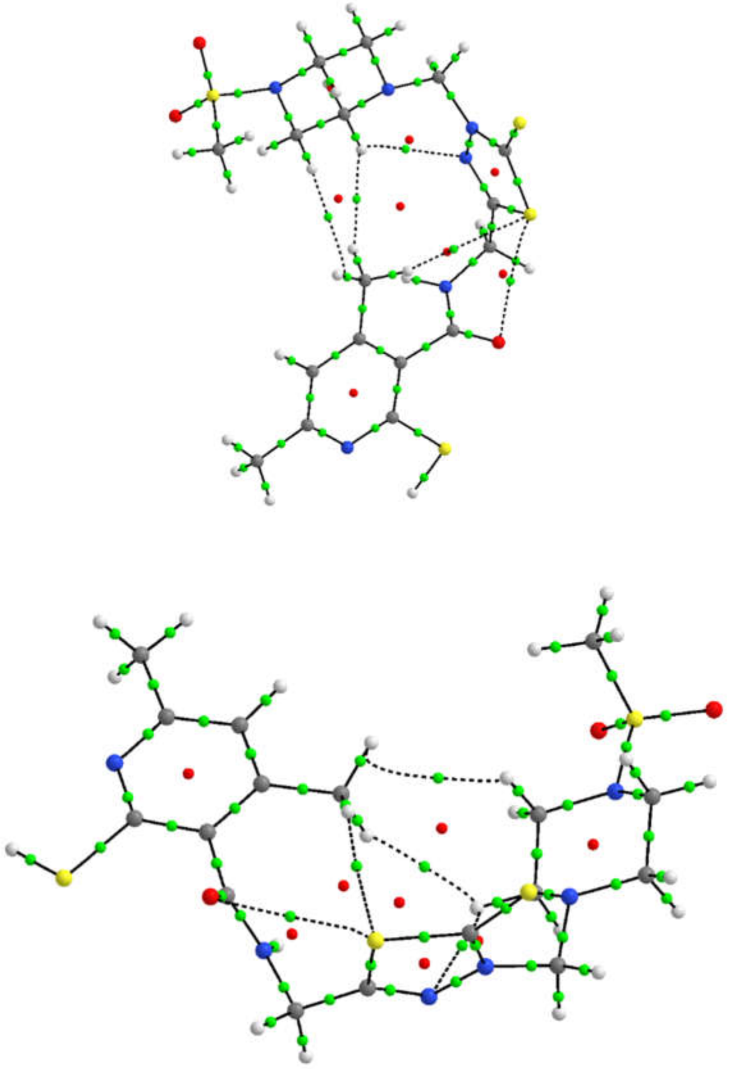

Compound 11

Figure S3 (Continuation). QTAIM molecular graphs of compounds **2** – **12** obtained as a result of the DFT/ωB97XD/6-311+G(d,p) simulations with solvent reaction field IEF-PCM and water as a solvent (two presentations to show the detected network of noncovalent interactions). Color coding: carbon – grey, oxygen – red, nitrogen – blue, sulphur – yellow, hydrogen – white, fluorine – green (compound **4**). Dotted lines indicate the network noncovalent interactions. Green and red spheres indicate the presence of Bond and Ring Critical Points (BCPs and RCPs), respectively.

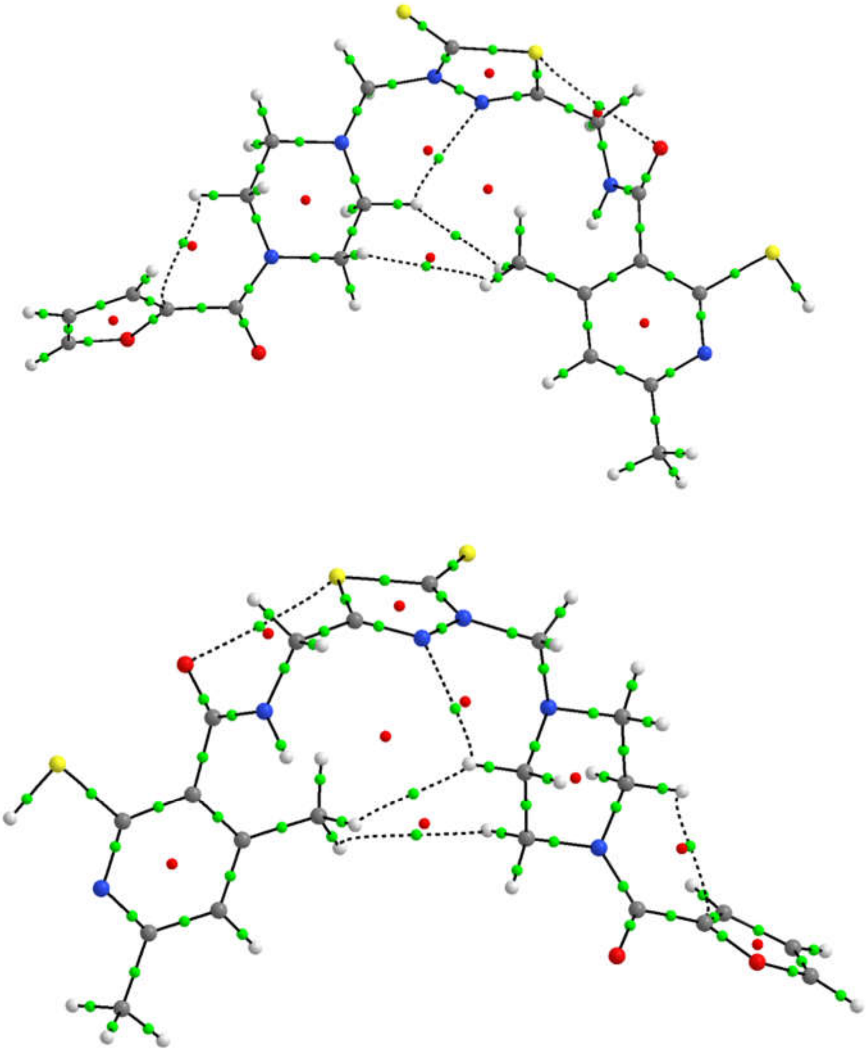

Compound 12

Figure S3 (Continuation). QTAIM molecular graphs of compounds **2** – **12** obtained as a result of the DFT/ωB97XD/6-311+G(d,p) simulations with solvent reaction field IEF-PCM and water as a solvent (two presentations to show the detected network of noncovalent interactions). Color coding: carbon – grey, oxygen – red, nitrogen – blue, sulphur – yellow, hydrogen – white, fluorine – green (compound **4**). Dotted lines indicate the network noncovalent interactions. Green and red spheres indicate the presence of Bond and Ring Critical Points (BCPs and RCPs), respectively.

Table S8. Physicochemical properties obtained for the set of the investigated compounds (**2 – 12**) according to SwissADME predictor.

**Compound**	**2**	**3**	**4**	**5**	**6**	**7**	**8**	**9**	**10**	**11**	**12**
Formula	$C_{11}H_{12}N_4OS_3$	$C_{22}H_{26}N_6OS_3$	$C_{22}H_{25}FN_6OS_3$	$C_{23}H_{28}N_6O_2S_3$	$C_{22}H_{25}N_7O_3S_3$	$C_{23}H_{25}N_7OS_3$	$C_{23}H_{28}N_6OS_3$	$C_{24}H_{28}N_6O_3S_3$	$C_{22}H_{32}N_6OS_3$	$C_{17}H_{24}N_6O_3S_4$	$C_{21}H_{24}N_6O_3S_3$
Molecular weight	312.43 g/mol	486.68 g/mol	504.67 g/mol	516.70 g/mol	531.67 g/mol	511.69 g/mol	500.70 g/mol	544.71 g/mol	492.72 g/mol	488.67 g/mol	504.65 g/mol
Num. heavy atoms	19	32	33	34	35	34	33	36	32	30	33
Num. arom. heavy atoms	11	17	17	17	17	17	17	17	11	11	16
Fraction Csp^3	0.27	0.36	0.36	0.39	0.36	0.35	0.39	0.42	0.64	0.53	0.38
Num. rotatable bonds	4	7	7	8	8	7	8	8	7	7	8
Num. H-bond acceptors	3	4	5	5	6	5	5	7	5	7	6
Num. H-bond donors	2	1	1	1	1	1	1	1	1	1	1
Molar Refractivity	79.76	140.44	140.40	146.94	149.27	145.16	144.69	150.76	142.13	129.17	137.38
TPSA	169.80 Å^2	165.42 Å^2	165.42 Å^2	174.65 Å^2	211.24 Å^2	189.21 Å^2	165.42 Å^2	183.88 Å^2	165.42 Å^2	207.94 Å^2	195.63 Å^2

Table S9. Lipophilicity and water solubility according to SwissADME predictor.

**Compound**	**2**	**3**	**4**	**5**	**6**	**7**	**8**	**9**	**10**	**11**	**12**
**Lipophilicity**											
Log Po/w (XLOGP3)	3.17	4.85	4.95	4.82	4.68	4.57	4.56	4.38	4.88	2.32	3.59
Log Po/w (WLOGP)	2.28	2.62	3.18	2.63	3.05	2.49	2.46	2.19	2.75	1.45	1.85
LogPo/w (MLOGP)	0.28	1.82	2.20	1.52	0.90	1.18	1.77	1.35	1.78	-0.27	0.41
Log Po/w (SILICOS-IT)	3.25	1.57	2.00	1.64	-0.17	1.62	1.97	1.81	1.67	-0.58	0.91
Consensus Log Po/w	2.24	2.72	3.08	2.65	2.11	2.46	2.69	2.43	2.77	0.73	1.69
**Water solubility**											
Log S (ESOL)	-3.94	-5.84	-6.01	-5.92	-5.92	-5.80	-5.67	-5.80	-5.76	-4.14	-5.06
Solubility	3.60e-02 mg/ml; 1.15e-04 mol/l	6.97e-04 mg/ml; 1.43e-06 mol/l	4.97e-04 mg/ml; 9.85e-07 mol/l	6.18e-04 mg/ml; 1.20e-06 mol/l	6.45e-04 mg/ml; 1.21e-06 mol/l	8.12e-04 mg/ml; 1.59e-06 mol/l	1.07e-03 mg/ml; 2.14e-06 mol/l	8.67e-04 mg/ml; 1.59e-06 mol/l	8.53e-04 mg/ml; 1.73e-06 mol/l	3.53e-02 mg/ml; 7.23e-05 mol/l	4.38e-03 mg/ml; 8.68e-06 mol/l
Class	Soluble	Moderately soluble	Poorly soluble	Moderately soluble	Moderately soluble	Moderately soluble	Moderately soluble	Moderately soluble	Moderately soluble	Moderately soluble	Moderately soluble
Log S (Ali)	-6.41	-8.06	-8.16	-8.22	-8.84	-8.27	-7.76	-7.96	-8.09	-6.33	-7.38
Solubility	1.23e-04 mg/ml; 3.92e-07 mol/l	4.26e-06 mg/ml; 8.75e-09 mol/l	3.48e-06 mg/ml; 6.89e-09 mol/l	3.11e-06 mg/ml; 6.02e-09 mol/l	7.62e-07 mg/ml; 1.43e-09 mol/l	2.77e-06 mg/ml; 5.41e-09 mol/l	8.76e-06 mg/ml; 1.75e-08 mol/l	6.00e-06 mg/ml; 1.10e-08 mol/l	4.01e-06 mg/ml; 8.15e-09 mol/l	2.31e-04 mg/ml; 4.73e-07 mol/l	2.08e-05 mg/ml; 4.12e-08 mol/l
Class	Poorly soluble	Poorly soluble	Poorly soluble	Poorly soluble	Poorly soluble	Poorly soluble	Poorly soluble	Poorly soluble	Poorly soluble	Poorly soluble	Poorly soluble
Log S (SILICOS-IT)	-4.14	-5.98	-6.24	-6.07	-5.79	-6.04	-6.37	-6.07	-4.89	-3.77	-5.12
Solubility	2.27e-02 mg/ml; 7.28e-05 mol/l	5.11e-04 mg/ml; 1.05e-06 mol/l	2.92e-04 mg/ml; 5.78e-07 mol/l	4.37e-04 mg/ml; 8.45e-07 mol/l	8.68e-04 mg/ml; 1.63e-06 mol/l	4.70e-04 mg/ml; 9.18e-07 mol/l	2.15e-04 mg/ml; 4.29e-07 mol/l	4.65e-04 mg/ml; 8.53e-07 mol/l	6.32e-03 mg/ml; 1.28e-05 mol/l	8.24e-02 mg/ml; 1.69e-04 mol/l	3.81e-03 mg/ml; 7.55e-06 mol/l
Class	Moderately soluble	Moderately soluble	Poorly soluble	Poorly soluble	Moderately soluble	Poorly soluble	Poorly soluble	Poorly soluble	Moderately soluble	Soluble	Moderately soluble

Table S10. Pharmacokinetics, druglikeness and medicinal chemistry.

Compound	2	3	4	5	6	7	8	9	10	11	12
**Pharmacokinetics**											
GI absorption	Low	Low	Low	Low	Low	Low	Low	Low	Low	Low	Low
BBB permeant	No	No	No	No	No	No	No	No	No	No	No
P-gp substrate	No	No	No	No	No	No	No	Yes	No	Yes	No
Log Kp (skin permeation)	-5.96 cm/s	-5.83 cm/s	-5.86 cm/s	-6.03 cm/s	-6.22 cm/s	-6.18 cm/s	-6.12 cm/s	-6.51 cm/s	-5.84 cm/s	-7.63 cm/s	-6.83 cm/s
**Druglikeness**											
Lipinski	Yes, 0 violation	Yes, 0 violation	Yes; 1 violation: MW>500	Yes; 1 violation: MW>500	Yes; 1 violation: MW>500	Yes; 1 violation: MW>500	Yes; 1 violation: MW>500	Yes; 1 violation: MW>500	Yes; 0 violation	Yes; 0 violation	Yes; 1 violation: MW>500
Ghose	Yes	No; 2 violations: MW>480, MR>130	No; 2 violations: MW>480, MR>130	No; 2 violations: MW>480, MR>130	No; 2 violations: MW>480, MR>130	No; 2 violations: MW>480, MR>130	No; 2 violations: MW>480, MR>130	No; 2 violations: MW>480, MR>130	No; 2 violations: MW>480, MR>130	No; 1 violation: MW>480	No; 2 violations: MW>480, MR>130
Veber	No; 1 violation: TPSA>140	No; 1 violation: TPSA>140	No; 1 violation: TPSA>140	No; 1 violation: TPSA>140	No; 1 violation: TPSA>140	No; 1 violation: TPSA>140	No; 1 violation: TPSA>140	No; 1 violation: TPSA>140	No; 1 violation: TPSA>140	No; 1 violation: TPSA>140	No; 1 violation: TPSA>140
Egan	No; 1 violation: TPSA>131.6	No; 1 violation: TPSA>131.6	No; 1 violation: TPSA>131.6	No; 1 violation: TPSA>131.6	No; 1 violation: TPSA>131.6	No; 1 violation: TPSA>131.6	No; 1 violation: TPSA>131.6	No; 1 violation: TPSA>131.6	No; 1 violation: TPSA>131.6	No; 1 violation: TPSA>131.6	No; 1 violation: TPSA>131.6
Muegge	No; 1 violation: TPSA>150	No; 1 violation: TPSA>150	No; 1 violation: TPSA>150	No; 1 violation: TPSA>150	No; 1 violation: TPSA>150	No; 1 violation: TPSA>150	No; 1 violation: TPSA>150	No; 1 violation: TPSA>150	No; 1 violation: TPSA>150	No; 1 violation: TPSA>150	No; 1 violation: TPSA>150
Bioavailability Score	0.55	0.55	0.55	0.55	0.55	0.55	0.55	0.55	0.55	0.55	0.55
**Medicinal chemistry**											
PAINS	0 alert	0 alert	0 alert	1 alert: anil_di_alk_C	1 alert: anil_di_alk_A	0 alert	0 alert	0 alert	0 alert	0 alert	0 alert
Brenk	2 alerts: thiocarbonyl_group, thiol_2	2 alerts: thiocarbonyl_group, thiol_2	2 alerts: thiocarbonyl_group, thiol_2	2 alerts: thiocarbonyl_group, thiol_2	3 alerts: nitro_group, thiocarbonyl_group, thiol_2	2 alerts: thiocarbonyl_group, thiol_2	2 alerts: thiocarbonyl_group, thiol_2	2 alerts: thiocarbonyl_group, thiol_2	2 alerts: thiocarbonyl_group, thiol_2	2 alerts: thiocarbonyl_group, thiol_2	2 alerts: thiocarbonyl_group, thiol_2
Leadlikeness	Yes	No; 2 violations: MW>350, XLOGP3>3.5	No; 2 violations: MW>350, XLOGP3>3.5	No; 3 violations: MW>350, Rotors>7, XLOGP3>3.5	No; 3 violations: MW>350, Rotors>7, XLOGP3>3.5	No; 2 violations: MW>350, XLOGP3>3.5	No; 3 violations: MW>350, Rotors>7, XLOGP3>3.5	No; 3 violations: MW>350, Rotors>7, XLOGP3>3.5	No; 2 violations: MW>350, XLOGP3>3.5	No; 1 violation: MW>350	No; 3 violations: MW>350, Rotors>7, XLOGP3>3.5
Synthetic accessibility	2.61	3.79	3.78	3.91	3.88	3.86	3.92	4.11	4.02	3.74	3.90

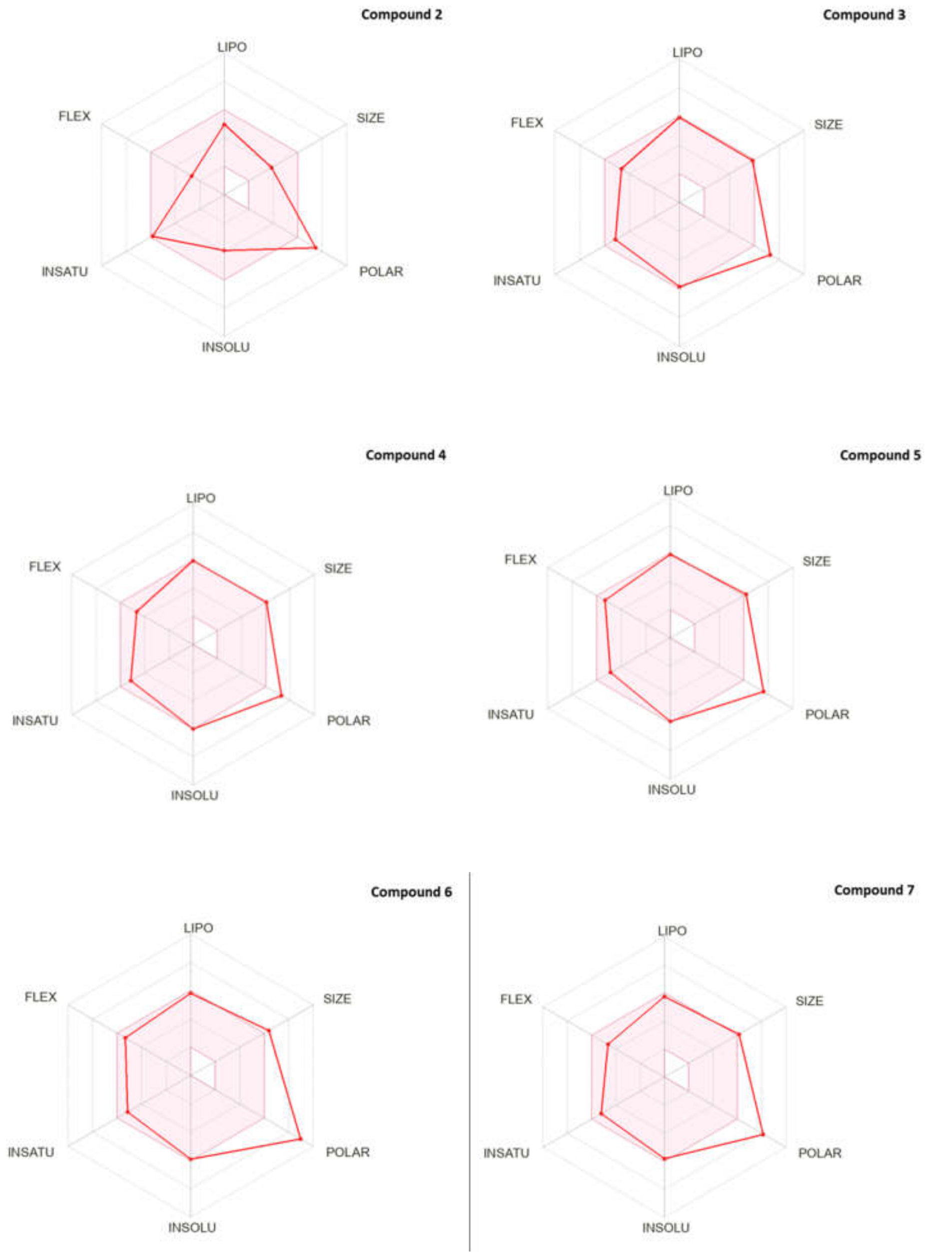

Figure S4. Bioavailability radar obtained as a result of SwissADME analysis.

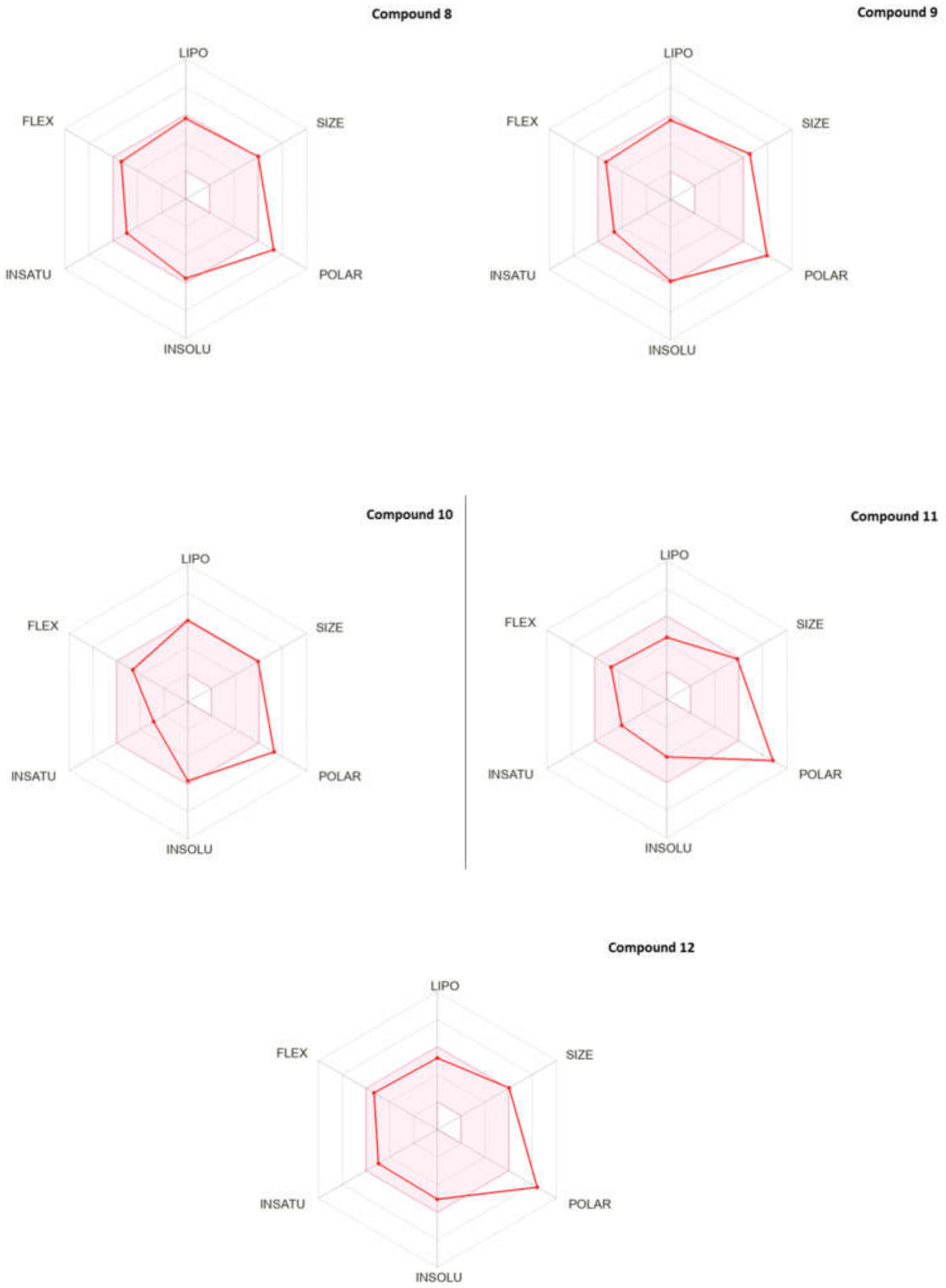

Figure S4 (Continuation). Bioavailability radar obtained as a result of SwissADME analysis.

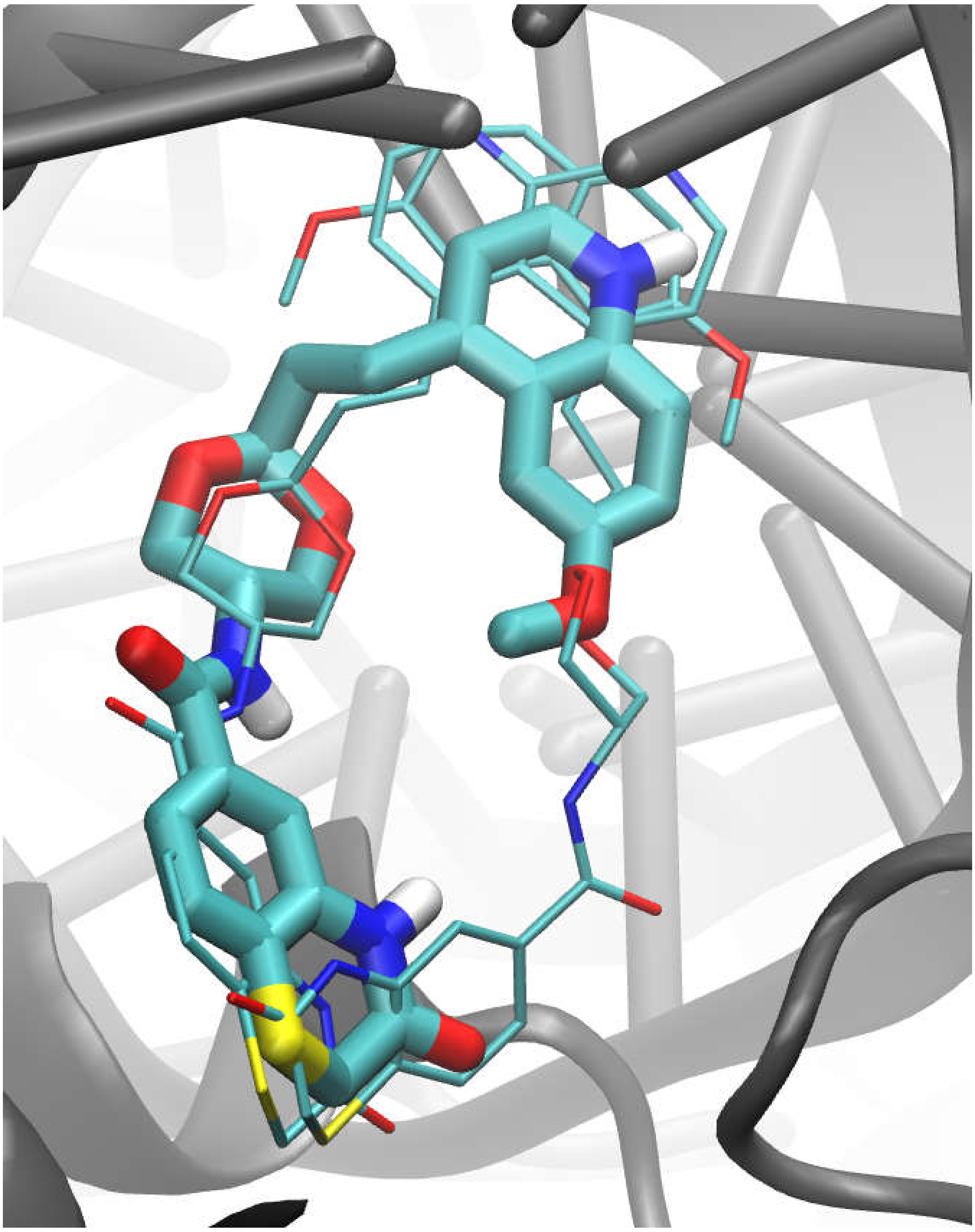

Figure S5. Redocking of the original ligand N-{(2r,5r)-2-[2-(6-methoxyquinolin-4-yl)ethyl]-1,3-dioxan-5-yl}-3-oxo-3,4-dihydro-2H-1,4-benzothiazine-6-carboxamide into the DNA gyrase (PDB deposit 9N26).

The experimental 9N26 deposit contains two equivalent positions of the ligand due to the crystal disorder phenomenon. The RMSD of the redocked ligand (calculated for the non-hydrogen atoms with respect to the left position of the original ligand) is 2.28 Å, which can be at least partially attributed to the disordered nature of the ligand, which increases the uncertainty of its experimental position. The graphical representation shown in Figure S5 indicates however that the docking procedure has correctly recognized the binding site and its extended nature.

Coordinates of compounds 2 – 12 (gas phase)

Compound 2

C	-5.563729	-0.038737	0.164702
C	-4.400398	0.721640	0.211349
C	-3.179139	0.052046	0.078197
C	-5.492064	-1.418174	0.008415
N	-4.322896	-2.054321	-0.099369
C	-3.205498	-1.341244	-0.064817
C	-6.721929	-2.276918	-0.045783
C	-4.467442	2.211258	0.399605
H	-6.529736	0.444888	0.253375
S	-1.687171	-2.273430	-0.177407
H	-2.327312	-3.450228	-0.263655
C	-1.895972	0.826984	0.162635
O	-1.612391	1.518344	1.122941
N	-1.081101	0.729983	-0.926058
H	-1.303349	0.056984	-1.643261
C	0.254010	1.293210	-0.908775
C	1.256972	0.372502	-0.273880
N	2.111060	-0.280892	-0.978206
N	2.868265	-1.066869	-0.178086
C	2.657853	-1.075152	1.163176
S	1.336822	0.052719	1.446049
H	3.588420	-1.630237	-0.603440
S	3.479328	-1.960813	2.282503
H	0.215550	2.240811	-0.370082
H	0.577381	1.480585	-1.932867
H	-5.494489	2.565732	0.304747
H	-4.085782	2.483692	1.385476
H	-3.849699	2.732819	-0.335589
H	-7.632438	-1.682375	0.040929
H	-6.699684	-3.009803	0.764117
H	-6.748912	-2.831070	-0.986940

Compound 3

C	4.637749	-2.396563	1.833063
C	4.133407	-1.138407	1.522276
C	4.301369	-0.676694	0.211375
C	5.298117	-3.144543	0.865282
N	5.463833	-2.694708	-0.381293
C	4.977720	-1.501711	-0.696251
C	5.865537	-4.502326	1.161099
C	3.433062	-0.316442	2.568018
H	4.518725	-2.794154	2.834375
S	5.261650	-0.969399	-2.376531
H	5.971126	-2.068221	-2.679816
C	3.806741	0.691774	-0.152831
O	4.150766	1.695053	0.444696
N	2.922628	0.739247	-1.185863
H	2.592506	-0.117664	-1.599281
C	2.382212	2.000088	-1.648868
C	1.179519	2.434329	-0.860823
N	0.005181	2.453474	-1.381773
N	-0.925458	2.858174	-0.476576
C	-0.512305	3.143458	0.788258

S	1.230755	2.914108	0.816940
C	-2.329951	2.838018	-0.874002
S	-1.438578	3.629207	2.066475
H	2.077959	1.900726	-2.691112
H	3.167606	2.754967	-1.580161
H	3.236752	-0.913908	3.458989
H	4.045364	0.544073	2.844340
H	2.483451	0.077606	2.197790
H	5.670510	-4.804701	2.190915
H	6.945063	-4.498763	0.992565
H	5.432160	-5.243380	0.485481
N	-2.964250	1.607926	-0.493698
H	-2.335499	3.019874	-1.956482
H	-2.812974	3.671389	-0.363826
C	-2.813213	0.542206	-1.470059
C	-4.344990	1.752013	-0.063484
C	-4.840602	0.435625	0.509340
C	-3.314793	-0.775507	-0.900596
N	-4.694661	-0.640477	-0.469633
C	-5.410559	-1.824814	-0.201118
C	-4.796956	-3.080087	-0.142976
C	-6.799509	-1.760978	-0.015844
C	-7.538364	-2.905930	0.228276
C	-5.549496	-4.228283	0.088108
C	-6.920015	-4.152785	0.280734
H	-4.387186	2.518721	0.714163
H	-5.005982	2.052919	-0.896036
H	-4.266329	0.205125	1.421497
H	-5.888009	0.539138	0.789512
H	-3.369662	0.770054	-2.397490
H	-1.756068	0.443288	-1.728700
H	-2.654929	-1.079782	-0.069720
H	-3.259081	-1.537136	-1.681286
H	-7.305318	-0.805564	-0.093868
H	-3.725674	-3.174022	-0.265835
H	-5.047552	-5.189040	0.127349
H	-7.501582	-5.048349	0.464752
H	-8.611708	-2.826374	0.362170

Compound 4

C	-4.654584	2.710317	1.800051
C	-4.265628	1.405840	1.515799
C	-4.469995	0.935934	0.212791
C	-5.240591	3.496592	0.814877
N	-5.442416	3.039537	-0.423676
C	-5.065960	1.801392	-0.713488
C	-5.683477	4.905616	1.082119
C	-3.646502	0.544594	2.580982
H	-4.503663	3.114602	2.794350
S	-5.393759	1.265596	-2.384854
H	-5.993853	2.421034	-2.712678
C	-4.099263	-0.478090	-0.122904
O	-4.523575	-1.434030	0.500218
N	-3.231855	-0.626535	-1.160544
H	-2.836374	0.188180	-1.601051
C	-2.813980	-1.940673	-1.601375

C	-1.646052	-2.466452	-0.816771
N	-0.484991	-2.604359	-1.349561
N	0.415500	-3.074663	-0.445504
C	-0.006291	-3.294316	0.829556
S	-1.720412	-2.905162	0.871286
C	1.810425	-3.194476	-0.857893
S	0.888345	-3.836166	2.107720
H	-2.515193	-1.891545	-2.648772
H	-3.663813	-2.619254	-1.507169
H	-3.404960	1.138370	3.463251
H	-4.332953	-0.254818	2.866210
H	-2.732362	0.063262	2.224883
H	-5.460400	5.210728	2.105399
H	-6.759350	4.995156	0.913769
H	-5.186904	5.590593	0.391050
N	2.562298	-2.023067	-0.507400
H	1.785828	-3.395304	-1.936661
H	2.219017	-4.060352	-0.336883
C	2.499747	-0.964312	-1.500675
C	3.927094	-2.288075	-0.084476
C	4.547800	-1.013738	0.461134
C	3.125548	0.309582	-0.955235
N	4.492706	0.053019	-0.538223
C	5.308145	1.176009	-0.278040
C	4.799494	2.472615	-0.167709
C	6.693103	0.998387	-0.153863
C	7.537524	2.069050	0.084536
C	5.642615	3.558796	0.052563
C	6.996599	3.339938	0.181964
H	3.904160	-3.041777	0.706513
H	4.550383	-2.663485	-0.915807
H	4.006765	-0.715069	1.373974
H	5.585049	-1.207609	0.733328
H	3.023961	-1.256021	-2.428880
H	1.453398	-0.773310	-1.751553
H	2.505047	0.679964	-0.120588
H	3.130652	1.065670	-1.743681
H	7.116023	0.007732	-0.272281
H	3.735533	2.654537	-0.244950
H	5.248550	4.564286	0.134848
F	7.818447	4.386962	0.402321
H	8.607938	1.931001	0.174996

Compound 5

C	-4.634375	2.986548	1.793969
C	-4.376237	1.650947	1.505218
C	-4.611155	1.211668	0.196736
C	-5.125073	3.833825	0.807302
N	-5.356580	3.406846	-0.436723
C	-5.104226	2.138385	-0.730637
C	-5.425299	5.279251	1.078829
C	-3.860465	0.725254	2.571353
S	-5.462821	1.647511	-2.409215
H	-5.940476	2.858962	-2.736283
C	-4.382022	-0.231269	-0.143431
O	-4.913704	-1.141437	0.465616

N	-3.516344	-0.461966	-1.167353
H	-3.026524	0.310083	-1.589593
C	-3.218425	-1.809974	-1.603667
C	-2.115876	-2.442755	-0.803688
N	-0.969659	-2.700611	-1.323655
N	-0.127672	-3.247751	-0.406700
C	-0.579420	-3.409688	0.866655
S	-2.247899	-2.854790	0.887718
C	1.251536	-3.511933	-0.806220
S	0.246227	-4.020435	2.159979
H	-2.902387	-1.790214	-2.646987
H	-4.129995	-2.404634	-1.521238
H	-3.578705	1.285405	3.463617
H	-4.624371	-0.007943	2.836763
H	-2.989858	0.162800	2.225085
H	-5.192655	5.552284	2.109032
H	-6.482367	5.481132	0.890096
H	-4.846236	5.914004	0.404101
N	2.113269	-2.411717	-0.479678
H	1.212505	-3.736555	-1.880063
H	1.570226	-4.400331	-0.260932
C	2.156503	-1.377884	-1.501144
C	3.445504	-2.804313	-0.049128
C	4.200576	-1.585972	0.453483
C	2.918395	-0.164325	-0.992878
N	4.254229	-0.554571	-0.581206
C	5.180016	0.490008	-0.340725
C	4.810908	1.824164	-0.210992
C	6.546579	0.180753	-0.256241
C	7.491220	1.160400	-0.037157
C	5.763356	2.827109	-0.009983
C	7.107746	2.499180	0.086173
H	3.346424	-3.526878	0.764864
H	4.021802	-3.269149	-0.869066
H	3.700288	-1.202004	1.358281
H	5.213795	-1.879163	0.728875
H	2.640609	-1.748611	-2.422895
H	1.134194	-1.084841	-1.752950
H	2.347498	0.288336	-0.162762
H	2.996005	0.570469	-1.797731
H	6.867231	-0.846534	-0.388856
H	3.768636	2.113369	-0.260674
H	5.427720	3.852347	0.077924
O	8.115258	3.390825	0.287945
H	8.544957	0.914788	0.022239
C	7.775928	4.751406	0.420535
H	8.713103	5.283744	0.575352
H	7.119399	4.919422	1.282482
H	7.289553	5.134041	-0.484678
H	-4.455788	3.367668	2.792824

Compound 6

C	3.247382	1.753318	1.844912
C	2.040893	1.956696	1.186917
C	1.984780	1.662749	-0.179983
C	4.357970	1.293469	1.143946

N	4.289377	0.992154	-0.155141
C	3.138610	1.160926	-0.792694
C	5.694530	1.111930	1.797848
C	0.837195	2.459573	1.933781
S	3.146465	0.721778	-2.520905
H	4.416646	0.286883	-2.474413
C	0.729861	1.965596	-0.937662
O	0.241472	3.083127	-0.966695
N	0.145315	0.921698	-1.581079
H	0.495248	-0.016685	-1.445255
C	-1.066765	1.129705	-2.343813
C	-2.282022	1.137448	-1.459042
N	-3.136370	0.179883	-1.492543
N	-4.092226	0.329524	-0.536826
C	-4.010316	1.418106	0.276026
S	-2.610248	2.345498	-0.242247
C	-5.000658	-0.818768	-0.310577
S	-4.989816	1.827348	1.547765
H	-1.184121	0.318092	-3.062353
H	-0.982032	2.073805	-2.884655
H	1.025057	2.466549	3.008220
H	0.580696	3.470377	1.611443
H	-0.036788	1.831839	1.737930
H	6.423011	1.782883	1.335392
H	6.039997	0.087654	1.647503
H	5.650278	1.323466	2.867306
N	-4.410771	-1.976056	0.277094
H	-5.425995	-1.080735	-1.279517
H	-5.782335	-0.425783	0.339355
C	-3.723505	-2.952377	-0.541235
C	-3.731559	-1.826945	1.547686
C	-2.255893	-1.439174	1.419918
C	-2.235604	-2.683501	-0.772793
N	-1.533909	-2.246173	0.434090
C	-0.164782	-2.113286	0.358716
C	0.548786	-2.512661	-0.796865
C	0.593361	-1.582876	1.428816
C	1.972582	-1.571239	1.392533
C	1.930600	-2.474639	-0.839363
C	2.639290	-2.035211	0.266732
H	-4.238897	-1.073267	2.153449
H	-3.799022	-2.785443	2.075348
H	-2.152440	-0.370993	1.183869
H	-1.808072	-1.594443	2.402501
H	-3.832678	-3.921149	-0.039170
H	-4.215021	-3.033019	-1.513246
H	-2.117674	-1.943895	-1.571739
H	-1.789640	-3.623789	-1.107780
H	0.109139	-1.200056	2.314764
H	0.028483	-2.879556	-1.669016
H	2.465222	-2.791541	-1.724968
N	4.095985	-2.081130	0.251707
H	2.542429	-1.197480	2.232785
H	3.329395	1.966373	2.904748
O	4.649861	-2.305028	-0.807922
O	4.676975	-1.910152	1.309585

Compound 7

C	-5.199809	2.306350	1.809532
C	-4.654924	1.068637	1.485632
C	-4.734413	0.652197	0.151075
C	-5.813902	3.078669	0.830401
N	-5.896250	2.671723	-0.438862
C	-5.370878	1.498648	-0.765934
C	-6.423318	4.415197	1.139129
C	-4.004957	0.220699	2.543095
S	-5.548115	1.024071	-2.477897
H	-6.257301	2.122807	-2.782197
C	-4.194322	-0.694587	-0.229262
O	-4.544165	-1.721567	0.322947
N	-3.263323	-0.695483	-1.221490
H	-2.933342	0.178504	-1.597439
C	-2.683179	-1.932951	-1.699232
C	-1.503353	-2.371159	-0.879584
N	-0.312970	-2.375825	-1.362999
N	0.590703	-2.791689	-0.435544
C	0.139022	-3.100652	0.810883
S	-1.605803	-2.880372	0.787118
C	2.005353	-2.775927	-0.793191
S	1.026379	-3.604767	2.108458
H	-2.342378	-1.799120	-2.726239
H	-3.457213	-2.702415	-1.680995
H	-3.890219	0.783149	3.470343
H	-4.607673	-0.668488	2.736945
H	-3.019924	-0.129459	2.224749
H	-6.292166	4.685117	2.187905
H	-7.491532	4.398282	0.910081
H	-5.967782	5.186742	0.514148
N	2.642052	-1.563397	-0.361321
H	2.039558	-2.929688	-1.879596
H	2.467759	-3.626393	-0.292158
C	2.491303	-0.460425	-1.295552
C	4.024061	-1.736631	0.061900
C	4.529575	-0.446712	0.680648
C	3.003835	0.830988	-0.675634
N	4.390271	0.656261	-0.275086
C	5.156659	1.802440	-0.046096
C	4.586837	3.054445	0.207987
C	6.565612	1.729339	-0.110076
C	7.354539	2.858118	0.117170
C	5.379888	4.175216	0.411107
C	6.766254	4.084396	0.379307
H	4.058044	-2.529734	0.812730
H	4.687001	-2.010451	-0.776243
H	3.946563	-0.228962	1.589171
H	5.573247	-0.569163	0.963637
H	3.041859	-0.651197	-2.234213
H	1.433101	-0.345652	-1.543306
H	2.361327	1.095353	0.180820
H	2.944560	1.630960	-1.416705
C	7.209694	0.505432	-0.479744
H	3.510171	3.149926	0.265882
H	4.905183	5.129198	0.612142
H	8.432411	2.762890	0.061069

H	-5.149635	2.668418	2.829951
H	7.381568	4.959485	0.546934
N	7.741411	-0.470281	-0.785445

Compound 8

C	-6.278443	2.000736	1.657582
C	-5.891600	0.762983	1.156841
C	-5.142831	0.744565	-0.025428
C	-5.937842	3.167244	0.981423
N	-5.233707	3.143287	-0.152547
C	-4.849965	1.969085	-0.636318
C	-6.359984	4.520389	1.475909
C	-6.266496	-0.505063	1.871141
S	-3.935962	2.022694	-2.169534
H	-4.012603	3.360156	-2.255881
C	-4.735013	-0.573291	-0.623237
O	-5.547380	-1.433335	-0.927887
N	-3.387662	-0.744989	-0.759955
H	-2.775396	0.026451	-0.543118
C	-2.828488	-1.889761	-1.448617
C	-1.575342	-2.355827	-0.778458
N	-0.425851	-2.279129	-1.345913
N	0.555368	-2.755431	-0.535506
C	0.213153	-3.194444	0.707390
S	-1.533370	-3.017565	0.836885
C	1.939565	-2.644364	-0.991907
S	1.206605	-3.793373	1.879262
H	-2.590252	-1.659360	-2.491319
H	-3.589385	-2.672784	-1.436875
H	-6.704812	-0.284384	2.845059
H	-6.983295	-1.074966	1.276898
H	-5.394724	-1.147300	2.020172
H	-6.690616	4.483528	2.515040
H	-7.185452	4.896674	0.864831
H	-5.534294	5.227676	1.384101
N	2.554420	-1.439692	-0.514872
H	1.895649	-2.714426	-2.086705
H	2.470800	-3.509095	-0.594505
C	2.309929	-0.282025	-1.361727
C	3.964845	-1.581600	-0.184899
C	4.467501	-0.312403	0.479961
C	2.830865	0.976708	-0.689474
N	4.245353	0.840211	-0.383471
C	4.795980	2.059857	0.183754
H	4.075350	-2.420274	0.506924
H	4.577530	-1.780668	-1.082061
H	3.950243	-0.186248	1.448934
H	5.537796	-0.406092	0.677587
H	2.802581	-0.398050	-2.344283
H	1.235326	-0.186412	-1.535409
H	2.237299	1.167401	0.224327
H	2.695401	1.828058	-1.362819
H	-6.848605	2.057862	2.577779
C	6.306102	2.041942	0.253808
H	4.385871	2.259172	1.191395
H	4.480105	2.893337	-0.453675

C	6.972995	2.453889	1.403564
C	7.055285	1.626852	-0.847550
C	8.363775	2.462766	1.454376
C	9.101977	2.050662	0.352868
C	8.442461	1.630130	-0.798724
H	6.534667	1.286961	-1.736280
H	6.400072	2.766652	2.271319
H	8.868609	2.785119	2.358331
H	10.185561	2.052085	0.390917
H	9.013216	1.303532	-1.661207

Compound 9

C	-5.841142	2.625940	1.746706
C	-5.377300	1.346024	1.462826
C	-5.538686	0.871409	0.155739
C	-6.456837	3.382804	0.756662
N	-6.616313	2.921172	-0.486371
C	-6.168125	1.706932	-0.775697
C	-6.980605	4.764030	1.023231
C	-4.725807	0.514770	2.532550
S	-6.440533	1.161040	-2.453496
H	-7.104729	2.279934	-2.784436
C	-5.088133	-0.519649	-0.179660
O	-5.480236	-1.500221	0.425756
N	-4.188305	-0.614275	-1.195758
H	-3.815587	0.224324	-1.610599
C	-3.679726	-1.899636	-1.626427
C	-2.497620	-2.349724	-0.816577
N	-1.318151	-2.407768	-1.322731
N	-0.407831	-2.816054	-0.398883
C	-0.842295	-3.066829	0.866096
S	-2.579866	-2.794681	0.869325
C	1.001560	-2.839714	-0.781337
S	0.055554	-3.550857	2.164811
H	-3.362855	-1.831844	-2.667476
H	-4.487675	-2.629341	-1.548985
H	-4.524682	1.117578	3.418858
H	-5.373211	-0.319620	2.808948
H	-3.784221	0.082230	2.185468
H	-6.790059	5.075591	2.051162
H	-8.057112	4.795220	0.838798
H	-6.512050	5.478889	0.342920
N	1.664270	-1.620055	-0.419035
H	1.012517	-3.044133	-1.860044
H	1.457178	-3.674938	-0.249439
C	1.537622	-0.565774	-1.412596
C	3.041779	-1.794629	0.015496
C	3.580377	-0.481270	0.554573
C	2.094134	0.737477	-0.864922
N	3.477824	0.566102	-0.453438
C	4.065293	1.817714	-0.002711
H	3.060697	-2.545840	0.808907
H	3.692437	-2.135129	-0.809618
H	3.016405	-0.209894	1.465938
H	4.629965	-0.604662	0.831094
H	2.076361	-0.827723	-2.341638

H	0.481620	-0.436508	-1.662584
H	1.461444	1.070566	-0.020815
H	2.049741	1.505281	-1.642969
H	-5.725604	3.033648	2.744363
C	5.561849	1.722163	0.183386
H	3.602579	2.167349	0.938650
H	3.845641	2.576219	-0.762533
C	6.163608	2.170175	1.351825
C	6.343409	1.180825	-0.852845
C	7.552783	2.112404	1.542332
C	8.293006	1.578482	0.518806
C	7.698035	1.121704	-0.651061
H	5.881764	0.813635	-1.760830
H	5.542877	2.570537	2.146184
H	8.019098	2.466442	2.452637
O	9.649188	1.431868	0.423226
C	9.842874	0.592737	-0.707609
H	9.978233	-0.444061	-0.371161
H	10.698929	0.945732	-1.280587
O	8.669426	0.678156	-1.505190

Compound 10

C	-4.775316	2.421951	1.922596
C	-4.325533	1.158920	1.553469
C	-4.458919	0.788289	0.209984
C	-5.350402	3.263879	0.977851
N	-5.484277	2.901094	-0.300425
C	-5.049301	1.703952	-0.670280
C	-5.856359	4.631102	1.335460
C	-3.716815	0.236552	2.572405
S	-5.287762	1.293011	-2.391327
H	-5.919515	2.448631	-2.652346
C	-4.020900	-0.581159	-0.218346
O	-4.437436	-1.597136	0.307339
N	-3.103409	-0.615344	-1.222127
H	-2.716194	0.245802	-1.572636
C	-2.607107	-1.873451	-1.739099
C	-1.445336	-2.402047	-0.947528
N	-0.260195	-2.447297	-1.441433
N	0.629232	-2.938212	-0.537825
C	0.172356	-3.270638	0.700192
S	-1.559763	-2.964778	0.700919
C	2.042453	-2.966069	-0.904333
S	1.042364	-3.865127	1.971657
H	-2.271939	-1.733797	-2.767195
H	-3.427035	-2.593789	-1.728829
H	-3.545031	0.761457	3.512748
H	-4.376578	-0.614138	2.753046
H	-2.765039	-0.171330	2.223025
H	-5.694859	4.857093	2.390381
H	-6.925061	4.700356	1.119005
H	-5.350908	5.386558	0.729400
N	2.725570	-1.789174	-0.450411
H	2.064070	-3.096070	-1.994680
H	2.473645	-3.845191	-0.425172
C	2.627702	-0.663984	-1.363758

C	4.101905	-2.020113	-0.047409
C	4.676412	-0.755509	0.566110
C	3.225768	0.588432	-0.743780
N	4.615685	0.367492	-0.365140
C	5.302754	1.552000	0.173945
H	4.115802	-2.819080	0.698585
H	4.734717	-2.322516	-0.901073
H	4.119160	-0.528180	1.493668
H	5.712671	-0.952053	0.840038
H	3.151444	-0.878242	-2.313747
H	1.575068	-0.482990	-1.596152
H	2.610154	0.889063	0.125307
H	3.169056	1.381246	-1.489219
H	-4.680434	2.749936	2.951329
C	4.981339	2.832702	-0.606077
H	4.979389	1.718761	1.222056
C	5.757020	4.040326	-0.073879
H	5.225975	2.670213	-1.664082
H	3.914426	3.059412	-0.550648
C	7.264230	3.800336	-0.072449
H	5.508606	4.922698	-0.671189
H	5.426974	4.253813	0.951019
C	7.595703	2.531857	0.708952
H	7.616151	3.691252	-1.106265
H	7.787583	4.662568	0.351776
C	6.822831	1.328728	0.168004
H	8.669496	2.325965	0.670899
H	7.342854	2.682211	1.766531
H	7.123005	1.125742	-0.867123
H	7.089243	0.445744	0.752201

Compound 11

C	-5.153681	1.634190	2.026897
C	-4.452271	0.539634	1.533038
C	-4.426501	0.356250	0.145321
C	-5.814252	2.493021	1.156198
N	-5.794485	2.307484	-0.166225
C	-5.119973	1.274746	-0.653392
C	-6.590328	3.680100	1.647656
C	-3.750872	-0.402995	2.470714
S	-5.165862	1.089722	-2.428403
H	-5.983405	2.142735	-2.587795
C	-3.713766	-0.835260	-0.422340
O	-3.964391	-1.975519	-0.077311
N	-2.743204	-0.559107	-1.334924
H	-2.505620	0.398386	-1.537180
C	-1.996771	-1.621772	-1.975044
C	-0.815220	-2.066412	-1.161176
N	0.389759	-1.842049	-1.546541
N	1.288886	-2.315794	-0.643012
C	0.815789	-2.902179	0.490055
S	-0.938869	-2.885958	0.375457
C	2.707447	-2.067863	-0.882947
S	1.692641	-3.531023	1.740512
H	-1.623646	-1.271438	-2.937722
H	-2.673276	-2.462004	-2.142071

H	-3.719174	0.010974	3.479203
H	-4.268531	-1.363773	2.496723
H	-2.727701	-0.605044	2.144795
H	-6.542213	3.771285	2.733640
H	-7.636847	3.590276	1.346885
H	-6.198664	4.594947	1.196951
N	3.162958	-0.908092	-0.169533
H	2.814503	-1.986207	-1.971851
H	3.246620	-2.947291	-0.530339
C	2.944288	0.340404	-0.887990
C	4.518122	-1.021459	0.351757
C	4.800330	0.142618	1.291013
C	3.223358	1.520165	0.034919
N	4.555192	1.436033	0.642619
H	4.594614	-1.956552	0.912166
H	5.274904	-1.029741	-0.452164
H	4.124528	0.086363	2.149475
H	5.828517	0.112103	1.646300
H	3.589603	0.416440	-1.778688
H	1.903021	0.381221	-1.218321
H	2.499711	1.526050	0.855751
H	3.127352	2.457289	-0.514852
H	-5.188982	1.816188	3.094852
S	5.812743	2.159743	-0.184001
O	7.042513	1.537274	0.266881
O	5.507568	2.238301	-1.603116
C	5.767481	3.826513	0.451599
H	4.787697	4.254936	0.244259
H	6.542474	4.384047	-0.073828
H	5.965754	3.791073	1.520798

Compound 12

C	-5.325966	2.142135	1.979111
C	-4.718846	0.956000	1.581453
C	-4.803747	0.603682	0.229179
C	-6.003724	2.926324	1.052855
N	-6.091049	2.579527	-0.233882
C	-5.507126	1.456987	-0.630839
C	-6.678797	4.209484	1.440939
C	-3.997994	0.094965	2.580833
S	-5.692945	1.058826	-2.361105
H	-6.460684	2.134180	-2.599302
C	-4.194735	-0.687024	-0.231974
O	-4.476835	-1.761721	0.266041
N	-3.280477	-0.580002	-1.233571
H	-3.012082	0.330306	-1.570551
C	-2.648497	-1.755558	-1.794175
C	-1.442662	-2.192056	-1.011983
N	-0.256683	-2.098122	-1.497439
N	0.672444	-2.537101	-0.606727
C	0.245200	-2.963885	0.612186
S	-1.507468	-2.831990	0.611182
C	2.083001	-2.409904	-0.960155
S	1.168177	-3.519311	1.865351
H	-2.322324	-1.538447	-2.811585
H	-3.385655	-2.560253	-1.823038

H	-3.886049	0.619616	3.530339
H	-4.549867	-0.831808	2.748599
H	-3.005787	-0.189010	2.221612
H	-6.548398	4.428490	2.501600
H	-7.747361	4.148467	1.221454
H	-6.272003	5.037089	0.855222
N	2.650960	-1.209750	-0.414189
H	2.119670	-2.454950	-2.055729
H	2.592732	-3.279063	-0.544051
C	2.473397	-0.035126	-1.256934
C	4.017581	-1.351314	0.057566
C	4.435861	-0.105063	0.832160
C	2.877122	1.214374	-0.487687
N	4.237034	1.089774	0.019848
H	4.067326	-2.216455	0.723481
H	4.728866	-1.505774	-0.774707
H	3.825651	-0.022602	1.737830
H	5.480221	-0.192480	1.120548
H	3.077422	-0.105623	-2.179160
H	1.422785	0.037585	-1.548489
H	2.205648	1.344894	0.367708
H	2.828334	2.092965	-1.127544
H	-5.273425	2.455112	3.015515
C	5.227939	1.805156	-0.599025
O	5.033521	2.493639	-1.583977
C	6.570158	1.786793	0.041951
C	6.998744	1.822354	1.333696
C	8.415944	1.978070	1.280893
C	8.730185	2.022511	-0.038042
O	7.624173	1.916346	-0.796716
H	6.376765	1.769000	2.213277
H	9.102286	2.051110	2.109075
H	9.660986	2.117433	-0.572074

XYZ coordinates (with solvent reaction field)

Compound 2

C	-5.567825	-0.019129	0.084938
C	-4.377317	0.694157	0.183673
C	-3.184586	-0.033050	0.175103
C	-5.545868	-1.406365	-0.004851
N	-4.398124	-2.095959	-0.004578
C	-3.256319	-1.426040	0.079995
C	-6.807978	-2.211407	-0.111585
C	-4.377490	2.191834	0.301901
H	-6.515249	0.506692	0.078264
S	-1.755273	-2.391338	0.087297
H	-2.395517	-3.571077	0.072775
C	-1.869549	0.676290	0.324718
O	-1.511288	1.172247	1.385229
N	-1.113512	0.730640	-0.793010
H	-1.440531	0.279949	-1.633601
C	0.203370	1.335743	-0.795806
C	1.257810	0.401068	-0.274470
N	2.122169	-0.156773	-1.046284
N	2.936655	-0.966340	-0.328688
C	2.758240	-1.080335	1.000457
S	1.407200	-0.050438	1.409460
H	3.666053	-1.461655	-0.822532
S	3.651393	-2.020817	2.047552
H	0.179324	2.244916	-0.193869
H	0.466745	1.601530	-1.818252
H	-5.362970	2.598848	0.075757
H	-4.104709	2.489582	1.317396
H	-3.649762	2.642860	-0.377199
H	-7.691825	-1.573436	-0.086819
H	-6.867034	-2.927583	0.711502
H	-6.811705	-2.780683	-1.044472

Compound 3

C	2.356719	-3.209362	0.236115
C	2.274902	-1.825090	0.100862
C	3.473072	-1.112255	0.009529
C	3.594252	-3.837473	0.265550
N	4.739385	-3.144745	0.189920
C	4.676138	-1.826281	0.077144
C	3.723557	-5.327085	0.392420
C	0.927041	-1.156327	0.048504
H	1.450592	-3.797980	0.318475
S	6.226986	-0.950308	-0.004089
H	6.954675	-2.064498	-0.182126
C	3.487777	0.383483	-0.109015
O	3.938401	1.104116	0.772485
N	2.968833	0.875761	-1.254538
H	2.594176	0.239405	-1.940431
C	2.798012	2.299863	-1.469138
C	1.560827	2.807279	-0.779242
N	0.409721	2.790048	-1.350521
N	-0.565976	3.214387	-0.502198
C	-0.204017	3.581258	0.748057

S	1.523905	3.364845	0.870117
C	-1.951986	3.111778	-0.956566
S	-1.189579	4.145508	1.970396
H	2.696208	2.480590	-2.537868
H	3.680441	2.827726	-1.108723
H	0.182608	-1.764834	0.563104
H	0.937343	-0.164654	0.504157
H	0.600422	-1.045331	-0.989888
H	2.747666	-5.808638	0.459528
H	4.302819	-5.579217	1.283969
H	4.256542	-5.731344	-0.471764
N	-2.542867	1.885758	-0.487959
H	-1.915396	3.200372	-2.049636
H	-2.497160	3.959827	-0.543603
C	-2.115814	0.716619	-1.244414
C	-3.995834	1.941630	-0.410693
C	-4.523256	0.678178	0.247583
C	-2.628457	-0.553540	-0.588925
N	-4.078261	-0.515035	-0.475182
C	-4.728546	-1.718698	-0.139048
C	-4.027357	-2.882222	0.202452
C	-6.131333	-1.783632	-0.177964
C	-6.798936	-2.961418	0.117636
C	-4.706919	-4.064974	0.485308
C	-6.093071	-4.116241	0.450839
H	-4.287270	2.804328	0.193550
H	-4.452032	2.049758	-1.409635
H	-4.169675	0.642171	1.290993
H	-5.610813	0.720069	0.263565
H	-2.477575	0.766756	-2.286264
H	-1.025442	0.683555	-1.272270
H	-2.147980	-0.668334	0.397192
H	-2.337981	-1.402874	-1.210087
H	-6.703588	-0.910156	-0.467489
H	-2.946701	-2.878291	0.259656
H	-4.136181	-4.950087	0.745151
H	-6.617849	-5.037373	0.675733
H	-7.882528	-2.980770	0.073437

Compound 4

C	-2.084105	3.364075	0.228085
C	-2.219490	1.983268	0.101899
C	-3.514287	1.466834	0.005559
C	-3.208152	4.178747	0.243323
N	-4.447258	3.673830	0.162802
C	-4.590795	2.360792	0.059137
C	-3.102906	5.671083	0.360054
C	-0.994094	1.109146	0.064136
H	-1.097659	3.804235	0.314778
S	-6.258685	1.736903	-0.027767
H	-6.804143	2.949688	-0.212540
C	-3.758455	-0.009710	-0.102116
O	-4.308615	-0.648054	0.786313
N	-3.322618	-0.583134	-1.244538
H	-2.855968	-0.015142	-1.933950
C	-3.348324	-2.020290	-1.439059

C	-2.190659	-2.678641	-0.739226
N	-1.057426	-2.853053	-1.319948
N	-0.143220	-3.383842	-0.463205
C	-0.536929	-3.642727	0.804261
S	-2.212735	-3.169570	0.931827
C	1.236386	-3.507479	-0.932074
S	0.373733	-4.295450	2.040557
H	-3.270554	-2.228931	-2.504830
H	-4.294429	-2.417917	-1.072891
H	-0.160969	1.603231	0.565104
H	-1.158851	0.141738	0.542242
H	-0.692640	0.924467	-0.971248
H	-2.063859	5.994604	0.426467
H	-3.636998	6.016619	1.248529
H	-3.564824	6.147913	-0.507988
N	2.011549	-2.371946	-0.508081
H	1.172560	-3.624090	-2.021185
H	1.651833	-4.414966	-0.495124
C	1.764039	-1.175399	-1.300671
C	3.438785	-2.646434	-0.423512
C	4.152476	-1.458050	0.197878
C	2.464134	0.019891	-0.678663
N	3.892154	-0.235246	-0.565718
C	4.712448	0.866998	-0.245586
C	4.192082	2.113691	0.119006
C	6.107429	0.732262	-0.324360
C	6.950770	1.794464	-0.042333
C	5.031556	3.192552	0.387919
C	6.394766	3.012461	0.308368
H	3.595333	-3.524240	0.208292
H	3.872801	-2.853522	-1.416540
H	3.809546	-1.335820	1.238376
H	5.221907	-1.662174	0.218730
H	2.112780	-1.309307	-2.339429
H	0.690895	-0.979221	-1.332240
H	2.010431	0.227015	0.305179
H	2.303327	0.889815	-1.318585
H	6.541689	-0.210897	-0.633126
H	3.124280	2.264177	0.205111
H	4.623960	4.156501	0.667284
F	7.218083	4.057012	0.575933
H	8.026405	1.685960	-0.109555

Compound 5

C	-1.773379	3.482834	0.232054
C	-2.121647	2.138077	0.127846
C	-3.478902	1.829253	0.007610
C	-2.755564	4.463802	0.203509
N	-4.056498	4.157843	0.100207
C	-4.402254	2.881495	0.016320
C	-2.418649	5.923304	0.293436
C	-1.050457	1.080344	0.136497
S	-6.144646	2.523505	-0.102987
H	-6.490500	3.802222	-0.321676
C	-3.946464	0.405920	-0.080040
O	-4.588979	-0.128309	0.814988

N	-3.599814	-0.242657	-1.212819
H	-3.050622	0.239179	-1.907195
C	-3.844877	-1.661279	-1.390042
C	-2.794400	-2.478533	-0.688742
N	-1.702979	-2.824181	-1.272565
N	-0.870242	-3.472521	-0.413645
C	-1.289250	-3.655785	0.858926
S	-2.877217	-2.941052	0.988445
C	0.473272	-3.800795	-0.888767
S	-0.476667	-4.420451	2.099435
H	-3.808248	-1.891600	-2.453548
H	-4.837508	-1.905939	-1.013147
H	-0.159267	1.446820	0.646934
H	-1.377664	0.162888	0.629276
H	-0.762748	0.825706	-0.887721
H	-1.345085	6.079200	0.402623
H	-2.929573	6.375327	1.146902
H	-2.758868	6.441846	-0.606496
N	1.407010	-2.778323	-0.496667
H	0.382603	-3.929990	-1.974716
H	0.759605	-4.749020	-0.435058
C	1.315977	-1.574072	-1.311980
C	2.782424	-3.253249	-0.429647
C	3.674405	-2.163893	0.141539
C	2.198183	-0.481946	-0.733803
N	3.576202	-0.940089	-0.656336
C	4.553016	0.042817	-0.362485
C	4.235050	1.335555	0.046138
C	5.912494	-0.274384	-0.518684
C	6.899826	0.656424	-0.265683
C	5.227954	2.290107	0.285811
C	6.566492	1.953387	0.138462
H	2.824568	-4.128048	0.224088
H	3.159343	-3.546835	-1.424313
H	3.377696	-1.964857	1.185095
H	4.704687	-2.518278	0.147684
H	1.615694	-1.778781	-2.354528
H	0.281646	-1.226173	-1.325335
H	1.806456	-0.197562	0.257988
H	2.146075	0.394446	-1.383429
H	6.196656	-1.262426	-0.863027
H	3.203488	1.631533	0.188255
H	4.928667	3.283141	0.594915
O	7.610138	2.800995	0.353184
H	7.945063	0.398886	-0.394147
C	7.315562	4.122934	0.767965
H	8.274600	4.624011	0.884291
H	6.784530	4.129995	1.725447
H	6.719445	4.651624	0.016920
H	-0.732148	3.764747	0.335585

Compound 6

C	-1.942574	3.529695	0.371640
C	-2.314223	2.198192	0.199900
C	-3.673898	1.922758	0.033511
C	-2.904334	4.530985	0.363195

N	-4.207943	4.256642	0.215066
C	-4.576479	2.992641	0.066641
C	-2.541732	5.977801	0.527202
C	-1.263232	1.120037	0.190372
S	-6.322069	2.677343	-0.111169
H	-6.635838	3.972029	-0.277859
C	-4.168217	0.515227	-0.127987
O	-4.857918	-0.037711	0.719306
N	-3.791283	-0.097380	-1.271055
H	-3.204885	0.397793	-1.924228
C	-4.060178	-1.501718	-1.513624
C	-3.061419	-2.372089	-0.800520
N	-1.955721	-2.725806	-1.351772
N	-1.179587	-3.432297	-0.485587
C	-1.657795	-3.653870	0.760029
S	-3.229352	-2.898461	0.851620
C	0.166178	-3.796145	-0.921609
S	-0.919117	-4.491142	1.999330
H	-3.980973	-1.692216	-2.582688
H	-5.074102	-1.736361	-1.191077
H	-0.378592	1.450262	0.735663
H	-1.618431	0.190837	0.639749
H	-0.955936	0.899995	-0.836339
H	-1.467545	6.107476	0.661687
H	-3.058954	6.399029	1.392605
H	-2.856508	6.544567	-0.352627
N	1.125922	-2.831229	-0.448967
H	0.116667	-3.875251	-2.014728
H	0.396425	-4.773316	-0.498532
C	1.121730	-1.596336	-1.218765
C	2.477316	-3.364511	-0.371889
C	3.390818	-2.357618	0.305019
C	2.009657	-0.551734	-0.564905
N	3.362366	-1.062624	-0.377588
C	4.403326	-0.177283	-0.205095
C	4.194398	1.220504	-0.199484
C	5.733848	-0.636827	-0.059558
C	6.785221	0.242221	0.064611
C	5.248785	2.102092	-0.078369
C	6.543730	1.614426	0.052180
H	2.468781	-4.280048	0.224120
H	2.875930	-3.607450	-1.371562
H	3.071660	-2.224725	1.348560
H	4.398682	-2.762739	0.311088
H	1.464646	-1.775530	-2.252637
H	0.105683	-1.201785	-1.270131
H	1.574294	-0.243157	0.396901
H	2.035762	0.314175	-1.225228
H	5.956790	-1.693848	-0.074346
H	3.197712	1.630853	-0.266351
H	5.067699	3.168480	-0.067804
N	7.648277	2.537637	0.182604
H	7.796980	-0.128272	0.159309
H	-0.899001	3.785307	0.512720
O	7.415808	3.737694	0.133670
O	8.773595	2.083114	0.336193

Compound 7

C	-2.297554	3.024384	0.827442
C	-2.458439	1.656147	0.631400
C	-3.671196	1.218889	0.092227
C	-3.320877	3.902909	0.489224
N	-4.484980	3.472360	-0.014344
C	-4.651116	2.169592	-0.200271
C	-3.181053	5.385976	0.672532
C	-1.368942	0.685037	0.995982
S	-6.216208	1.630945	-0.859112
H	-6.666610	2.874272	-1.089405
C	-3.902127	-0.249806	-0.124965
O	-4.513017	-0.946326	0.673997
N	-3.348483	-0.740994	-1.256277
H	-2.812164	-0.118355	-1.841295
C	-3.266025	-2.165597	-1.517341
C	-2.085868	-2.754781	-0.793995
N	-0.910488	-2.764624	-1.313974
N	0.013779	-3.227748	-0.429613
C	-0.411749	-3.589213	0.801966
S	-2.139020	-3.333559	0.847461
C	1.420566	-3.172132	-0.826378
S	0.506380	-4.176490	2.064653
H	-3.134452	-2.321865	-2.586663
H	-4.193054	-2.643624	-1.204149
H	-0.535500	1.198036	1.475058
H	-1.742568	-0.080177	1.681204
H	-0.989079	0.170313	0.109425
H	-2.215506	5.645645	1.107438
H	-3.973610	5.760298	1.324858
H	-3.279900	5.893233	-0.290457
N	2.029643	-1.953073	-0.362433
H	1.428902	-3.286496	-1.917034
H	1.919750	-4.026219	-0.370777
C	1.733757	-0.802432	-1.207933
C	3.463564	-2.078393	-0.131999
C	3.991247	-0.806601	0.507638
C	2.254330	0.470636	-0.563052
N	3.690404	0.351024	-0.343281
C	4.394235	1.528652	-0.057637
C	3.769950	2.693802	0.398364
C	5.790007	1.578566	-0.267328
C	6.524696	2.733292	0.015863
C	4.503988	3.843658	0.657671
C	5.883690	3.868990	0.481631
H	3.640888	-2.917583	0.544840
H	4.014337	-2.265436	-1.069104
H	3.523367	-0.674432	1.495541
H	5.066241	-0.898939	0.653155
H	2.185492	-0.918667	-2.207868
H	0.653319	-0.718571	-1.340723
H	1.712922	0.640100	0.380849
H	2.061318	1.313757	-1.228510
C	6.469799	0.464334	-0.851170
H	2.701248	2.700519	0.567696
H	3.989213	4.727721	1.016375
H	7.594583	2.734256	-0.154624

H	-1.373292	3.406978	1.244065
H	6.451471	4.765935	0.694362
N	7.031424	-0.416926	-1.339150

Compound 8

C	-2.814476	3.227927	0.551477
C	-2.776403	1.853996	0.325412
C	-3.983594	1.213618	0.034267
C	-4.018089	3.916194	0.474350
N	-5.173255	3.291486	0.206152
C	-5.152898	1.982048	0.004159
C	-4.097237	5.398532	0.694042
C	-1.466869	1.113788	0.389160
S	-6.714710	1.191354	-0.332391
H	-7.375165	2.348209	-0.498894
C	-4.035170	-0.269126	-0.190301
O	-4.572867	-1.033010	0.601224
N	-3.433092	-0.697708	-1.321099
H	-2.979918	-0.025737	-1.920543
C	-3.237827	-2.108085	-1.598046
C	-2.041729	-2.635152	-0.853742
N	-0.864053	-2.623411	-1.367848
N	0.066951	-3.053545	-0.473612
C	-0.356590	-3.415880	0.758193
S	-2.088456	-3.193231	0.795957
C	1.473417	-2.974100	-0.866192
S	0.565954	-3.981963	2.027762
H	-3.064059	-2.235181	-2.665135
H	-4.137014	-2.657392	-1.321644
H	-0.749693	1.660994	1.001442
H	-1.579478	0.110243	0.804049
H	-1.038364	1.014737	-0.612324
H	-3.119685	5.820226	0.929311
H	-4.783986	5.620596	1.514349
H	-4.484901	5.889489	-0.202130
N	2.064212	-1.751527	-0.389523
H	1.484212	-3.077389	-1.958791
H	1.985890	-3.825021	-0.418640
C	1.691136	-0.587956	-1.186378
C	3.512627	-1.842861	-0.247135
C	4.051858	-0.568590	0.379806
C	2.239781	0.677162	-0.552339
N	3.687612	0.594756	-0.419702
C	4.239530	1.826437	0.129877
H	3.750713	-2.692346	0.398111
H	4.005511	-2.002937	-1.221505
H	3.658825	-0.476847	1.409356
H	5.140558	-0.632321	0.442514
H	2.072989	-0.679036	-2.218366
H	0.602738	-0.523630	-1.239768
H	1.755928	0.827044	0.430540
H	1.988108	1.534816	-1.181881
H	-1.901832	3.761959	0.788469
C	5.748497	1.867811	0.060513
H	3.917150	1.978545	1.175372
H	3.830285	2.658730	-0.452148

C	6.513503	2.086827	1.203161
C	6.400097	1.691001	-1.161726
C	7.903986	2.134208	1.131083
C	8.543733	1.956858	-0.089666
C	7.786301	1.733885	-1.237959
H	5.810229	1.510700	-2.054360
H	6.018863	2.216631	2.160879
H	8.485260	2.304061	2.030702
H	9.625943	1.989997	-0.148609
H	8.279082	1.594785	-2.194058

Compound 9

C	-5.756261	3.695833	0.045722
C	-5.879681	2.392288	-0.424852
C	-4.819841	1.514409	-0.184578
C	-4.615587	4.083595	0.739654
N	-3.607348	3.233617	0.972183
C	-3.709668	1.991146	0.519991
C	-4.447371	5.478202	1.267983
C	-7.111435	1.946747	-1.160569
H	-3.544049	5.930754	0.851894
H	-4.333936	5.455824	2.354704
H	-5.302769	6.106291	1.017862
H	-7.723792	2.802203	-1.445657
H	-6.852906	1.387001	-2.062663
H	-7.710559	1.287314	-0.527865
H	-6.551128	4.411585	-0.127589
S	-2.343230	0.895154	0.869728
H	-1.678709	1.796049	1.610684
C	-4.908139	0.079431	-0.618056
O	-5.667744	-0.717666	-0.084621
N	-4.084387	-0.263095	-1.634022
H	-3.448030	0.427972	-2.001272
C	-3.915928	-1.640735	-2.055454
C	-2.949485	-2.358718	-1.154491
N	-1.697333	-2.436934	-1.426019
N	-1.014480	-3.053534	-0.423322
C	-1.716100	-3.482008	0.650735
S	-3.388025	-3.058851	0.379973
S	-1.152609	-4.269268	2.013163
H	-3.516203	-1.652129	-3.068129
H	-4.885539	-2.137559	-2.054808
C	0.435848	-3.145085	-0.585717
N	1.036188	-1.839147	-0.491119
C	1.039462	-1.311888	0.869779
C	2.381998	-1.820838	-1.054953
C	1.599541	0.099057	0.880728
N	2.946988	0.119143	0.328584
C	2.938639	-0.408065	-1.030049
H	0.624403	-3.558623	-1.576771
H	0.788329	-3.853044	0.174153
H	2.338151	-2.174007	-2.088331
H	3.061257	-2.486918	-0.495163
H	0.015783	-1.296013	1.251901
H	1.638446	-1.952459	1.539680
H	0.923340	0.759872	0.307304

H	1.631389	0.466000	1.909918
H	3.960649	-0.418401	-1.415615
H	2.333050	0.233048	-1.697113
C	3.522387	1.457340	0.389104
C	4.992237	1.471132	0.041124
C	5.473418	2.267633	-0.990478
C	5.874986	0.660512	0.778609
C	7.200716	0.694417	0.429272
C	6.835214	2.301417	-1.332319
C	7.673616	1.497143	-0.602441
H	2.983870	2.155600	-0.275702
H	3.388679	1.825102	1.411799
H	5.513579	0.031601	1.582979
H	4.777486	2.877141	-1.556641
H	7.204133	2.928107	-2.134277
O	8.252869	0.039148	1.007167
O	9.031706	1.361859	-0.693241
C	9.336650	0.214555	0.096671
H	9.408132	-0.664524	-0.554137
H	10.255427	0.387467	0.652097

Compound 10

C	-2.213289	3.251149	0.455049
C	-2.266220	1.873816	0.251488
C	-3.522010	1.299546	0.040001
C	-3.377635	4.006646	0.432857
N	-4.580042	3.445514	0.241577
C	-4.645904	2.134489	0.062920
C	-3.360130	5.494145	0.629940
C	-0.996342	1.065261	0.253493
S	-6.267852	1.432421	-0.170199
H	-6.866929	2.624044	-0.323765
C	-3.676935	-0.180044	-0.156216
O	-4.233822	-0.893976	0.668155
N	-3.151605	-0.666511	-1.301501
H	-2.682245	-0.037968	-1.934616
C	-3.085310	-2.090719	-1.568442
C	-1.921876	-2.718193	-0.850007
N	-0.753544	-2.785801	-1.381031
N	0.153275	-3.305056	-0.509555
C	-0.281812	-3.663408	0.719444
S	-1.989874	-3.307640	0.787517
C	1.557877	-3.313204	-0.916684
S	0.610029	-4.328499	1.963430
H	-2.950815	-2.239192	-2.638426
H	-4.021344	-2.558893	-1.265527
H	-0.233728	1.557202	0.858050
H	-1.146017	0.056070	0.641310
H	-0.603521	0.978827	-0.763851
H	-2.346808	5.864382	0.787654
H	-3.972863	5.766015	1.492870
H	-3.784275	5.992596	-0.245244
N	2.233886	-2.145580	-0.416612
H	1.550712	-3.389207	-2.011749
H	2.016226	-4.207544	-0.495656
C	1.926428	-0.938003	-1.170151

C	3.675599	-2.323135	-0.319962
C	4.305423	-1.099350	0.323008
C	2.565951	0.277409	-0.523122
N	4.014371	0.121021	-0.428026
C	4.704956	1.285531	0.157946
H	3.885144	-3.199939	0.298590
H	4.130020	-2.487444	-1.312441
H	3.936663	-1.013612	1.362011
H	5.381964	-1.259599	0.367441
H	2.279719	-1.025728	-2.213142
H	0.844485	-0.796841	-1.198635
H	2.112348	0.438420	0.472686
H	2.325729	1.140388	-1.143008
H	-1.259558	3.734600	0.631123
C	4.195730	2.618777	-0.403605
H	4.524312	1.300204	1.251585
C	4.964248	3.813443	0.167051
H	4.294184	2.601849	-1.497182
H	3.136631	2.754110	-0.175640
C	6.468080	3.693282	-0.063406
H	4.579068	4.735981	-0.277274
H	4.771002	3.879915	1.245473
C	6.983884	2.373103	0.502715
H	6.675284	3.731228	-1.140572
H	6.992571	4.538573	0.392425
C	6.219258	1.182731	-0.077605
H	8.052170	2.256722	0.297457
H	6.871740	2.379402	1.594561
H	6.387539	1.128940	-1.160626
H	6.620127	0.263064	0.352688

Compound 11

C	-2.593079	2.874388	0.599645
C	-2.406890	1.510472	0.389688
C	-3.531555	0.746950	0.066954
C	-3.859143	3.433360	0.478348
N	-4.934153	2.690524	0.181055
C	-4.771727	1.388779	-0.008254
C	-4.098821	4.900900	0.680677
C	-1.035085	0.901207	0.505320
S	-6.229194	0.434876	-0.384647
H	-7.009448	1.512277	-0.565232
C	-3.411993	-0.734240	-0.144986
O	-3.834067	-1.549578	0.664749
N	-2.783827	-1.095694	-1.285095
H	-2.428788	-0.376791	-1.896405
C	-2.411488	-2.471806	-1.553098
C	-1.138045	-2.822052	-0.833636
N	0.014745	-2.669643	-1.380811
N	1.015613	-2.952566	-0.503380
C	0.674070	-3.330139	0.749304
S	-1.070496	-3.337066	0.829474
C	2.389570	-2.693896	-0.932470
S	1.695933	-3.726638	2.006728
H	-2.247919	-2.589915	-2.622838
H	-3.222657	-3.131646	-1.247077

H	-0.377042	1.551006	1.082588
H	-1.063282	-0.077528	0.988899
H	-0.590901	0.769086	-0.485217
H	-3.177519	5.425409	0.935350
H	-4.825531	5.055657	1.481824
H	-4.513391	5.341261	-0.229582
N	2.814471	-1.390064	-0.494969
H	2.392419	-2.821367	-2.021229
H	3.022033	-3.455739	-0.478914
C	2.333346	-0.309162	-1.345516
C	4.249042	-1.286230	-0.268171
C	4.566490	0.038268	0.407584
C	2.617469	1.030908	-0.691101
N	4.060045	1.133000	-0.432826
H	4.564250	-2.096903	0.392118
H	4.821067	-1.360049	-1.207367
H	4.094935	0.059302	1.399445
H	5.643956	0.147132	0.519046
H	2.806435	-0.342103	-2.341009
H	1.255247	-0.409437	-1.483230
H	2.049928	1.102578	0.246531
H	2.316454	1.839364	-1.355694
H	-1.748512	3.501910	0.858906
S	4.656533	2.650704	-0.134816
O	6.098557	2.517545	0.010597
O	4.127873	3.523599	-1.172731
C	3.986832	3.185239	1.428944
H	2.900836	3.218458	1.350710
H	4.384980	4.180268	1.623798
H	4.306903	2.488637	2.202636

Compound 12

C	-2.887944	3.152602	0.718926
C	-2.783466	1.795799	0.421326
C	-3.953922	1.119940	0.067562
C	-4.119115	3.790906	0.649191
N	-5.238961	3.131692	0.320373
C	-5.155693	1.837314	0.049820
C	-4.269708	5.254442	0.944592
C	-1.443179	1.111963	0.477998
S	-6.672964	0.999428	-0.366404
H	-7.379508	2.134303	-0.489089
C	-3.934956	-0.349159	-0.236646
O	-4.458974	-1.177070	0.497698
N	-3.286277	-0.690717	-1.371085
H	-2.846954	0.030168	-1.921920
C	-3.031293	-2.075379	-1.719181
C	-1.844956	-2.605893	-0.961888
N	-0.652068	-2.534337	-1.434254
N	0.260698	-2.990063	-0.533677
C	-0.192639	-3.433198	0.661119
S	-1.929894	-3.255378	0.652612
C	1.675075	-2.854519	-0.873537
S	0.704628	-4.048272	1.925381
H	-2.814617	-2.134634	-2.784406
H	-3.919546	-2.670048	-1.508718

H	-0.772179	1.647691	1.149969
H	-1.520581	0.078026	0.819556
H	-0.981001	1.103158	-0.513395
H	-3.318712	5.704278	1.231013
H	-4.988340	5.402284	1.754212
H	-4.653565	5.776078	0.064218
N	2.220837	-1.653176	-0.294649
H	1.723889	-2.881582	-1.969103
H	2.195132	-3.721514	-0.467739
C	1.839804	-0.444598	-1.018019
C	3.664670	-1.720699	-0.114920
C	4.163577	-0.505170	0.658894
C	2.301373	0.781736	-0.248860
N	3.737551	0.724684	-0.001933
H	3.911340	-2.620725	0.453295
H	4.192964	-1.769345	-1.082660
H	3.759546	-0.521780	1.676441
H	5.247519	-0.544999	0.713081
H	2.275837	-0.434481	-2.031963
H	0.753840	-0.412714	-1.121585
H	1.789976	0.817758	0.719270
H	2.077226	1.689306	-0.803790
H	-2.005472	3.712133	1.005987
C	4.544636	1.684413	-0.515753
O	4.146408	2.544583	-1.298758
C	5.955170	1.724981	-0.053391
C	6.567933	1.540008	1.147934
C	7.930816	1.922653	0.966771
C	8.037721	2.308734	-0.328920
O	6.848161	2.205294	-0.956402
H	6.104471	1.192547	2.057714
H	8.719395	1.911218	1.701936
H	8.860383	2.660143	-0.929573
